# The causal impact of genetically predicted inflammatory bowel disease on extraintestinal manifestations: a mendelian randomization study

**DOI:** 10.1186/s12876-024-03566-4

**Published:** 2025-03-04

**Authors:** Xingcan Fan, Anqi He, Kaiyu Li, Maorun Zhang, Qi Zhang, Wanyi Xiao, Gang Liu

**Affiliations:** Department of General Surgery, Tianjin Medical University General Hospital, Tianjin, 300052 China

**Keywords:** Inflammatory bowel disease (IBD), Extraintestinal manifestations (EIMs), Mendelian randomization (MR)

## Abstract

**Background:**

Extraintestinal manifestations (EIMs) significantly affect the life quality of people with inflammatory bowel disease (IBD) and are crucial factors impacting occurrence rates and mortality among IBD patients. This study performed a Mendelian randomization (MR) analysis to investigate the causal relationships between genetically predicted IBD and the development of EIMs, including erythema nodosum (EN), episcleritis, scleritis, uveitis, primary sclerosing cholangitis (PSC), and spondyloarthritis. To further investigate differences between subtypes, separate analyses were conducted for ulcerative colitis (UC) and Crohn’s disease (CD).

**Methods:**

The study was conducted based on genome-wide association studies (GWAS) data. We carefully selected SNPs associated with both exposure and outcome by comparing and integrating data from GWAS and relevant literature, and prioritizing studies with large sample sizes, high quality, and as much population homogeneity as possible. The SNPs associated with IBD, UC and CD were extracted from the International Inflammatory Bowel Disease Genetics Consortium. And the SNPs associated with EIMs were extracted from the UK Biobank, the International PSC Study Group and the FinnGen study. A series of quality control steps were taken in our analysis to select eligible instrumental SNPs which were strongly associated with exposure. The causal effects were estimated using a primary analysis that employed inverse-variance weighting (IVW) and complementary analysis that utilized MR-Egger weighted by the median. A sensitivity analysis was conducted using the Cochran Q statistic, a funnel plot, the MR-Egger intercept, and a leave-one-out approach. Reverse causality analysis was also performed to ensure the robustness of the findings. Furthermore, a fixed-effects meta-analysis was employed to combine MR outcomes from various data origins, bolstering the strength and dependability of our findings.

**Results:**

Our findings indicated that genetically predicted IBD had a robust causal relationship with an increased risk of specific conditions, including EN (OR, 1.20; 95% CI, 1.09–1.32; *p* < 0.01), uveitis (OR, 1.15; 95% CI, 1.11–1.20; *p* < 0.01), PSC (OR, 1.21; 95% CI, 1.13–1.28; *p* < 0.01), and spondyloarthritis (OR, 1.19; 95% CI, 1.14–1.23; *p* < 0.01). In subgroup analyses, the causal effects of both UC and CD on EN, uveitis, PSC, and spondyloarthritis were also significant and robust. Additionally, no significant evidence of causality was observed between genetically predicted IBD, UC, and CD, and the occurrence of both episcleritis and scleritis. The results of reverse causality analysis indicated a robust causal association between genetically predicted PSC and the elevated risk of IBD (OR, 1.21; 95% CI, 1.15–1.29; *p* < 0.01), UC (OR, 1.27; 95% CI, 1.17–1.37; *p* < 0.01), and CD (OR, 1.10; 95% CI, 1.02–1.20; *p* < 0.01). Additionally, spondyloarthritis had a causal relationship with an increased risk of both IBD (OR, 1.03; 95% CI, 1.01–1.06; *p* < 0.01) and UC (OR, 1.05; 95% CI, 1.02–1.08; *p* < 0.01).

**Graphical abstract:**

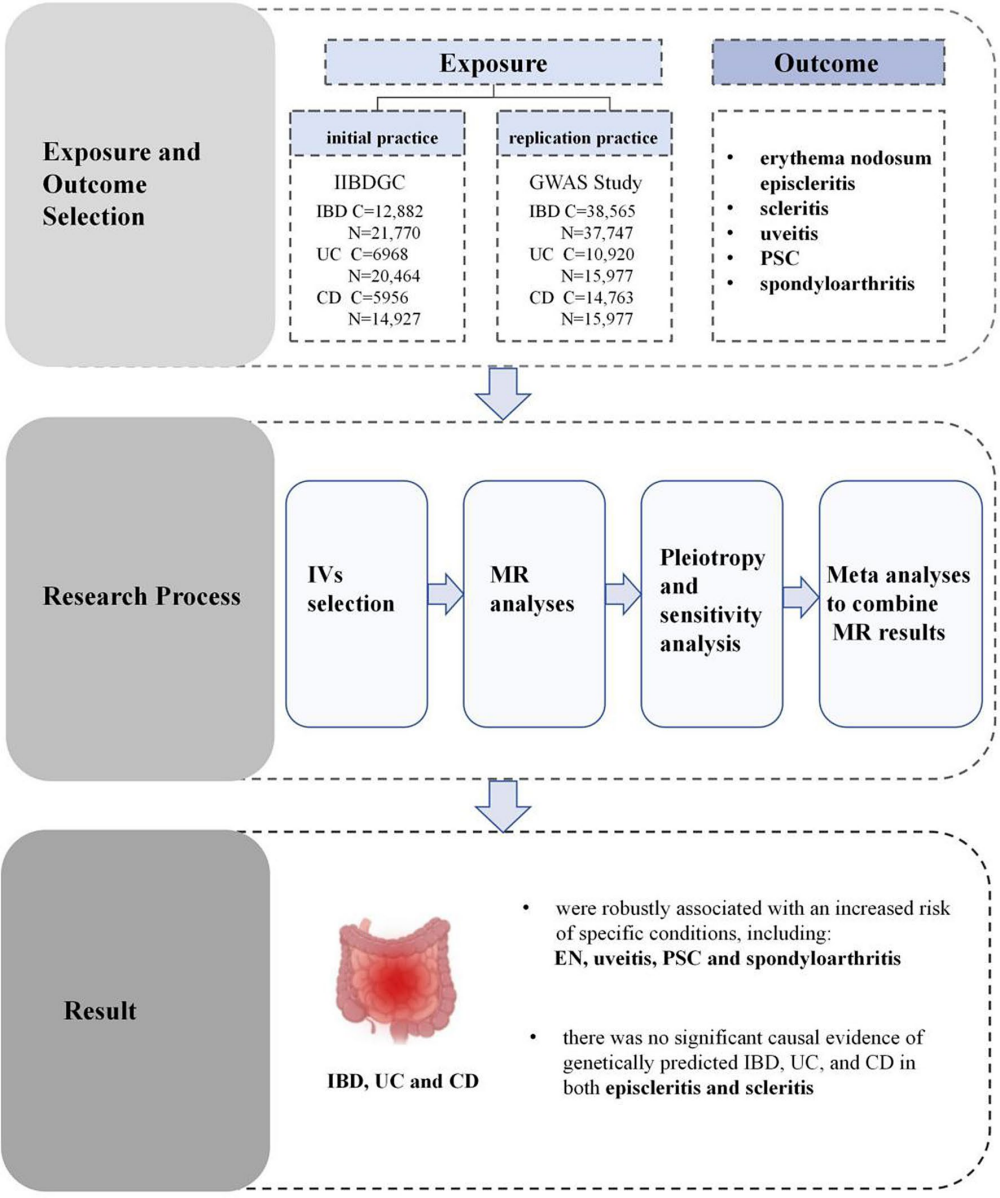

**Supplementary Information:**

The online version contains supplementary material available at 10.1186/s12876-024-03566-4.

## Introduction

Inflammatory bowel disease (IBD), which comprises Crohn’s disease (CD) and ulcerative colitis (UC), is a chronic, systemic, autoimmune disease that can not only affect the alimentary canal but also could impact the function of other organs. Typically, when organs outside the alimentary tract are affected, we refer to this as extraintestinal manifestations (EIMs) of IBD [1]. EIMs need to be distinguished from extra-intestinal complications of IBD, which are consequences of intestinal inflammation either directly or indirectly [2]. The European Crohn’s and Colitis Organization (ECCO) offers a comprehensive description of EIMs. It is an inflammatory pathology affecting extraintestinal sites in patients with IBD and the pathogenesis of this condition can either depend on the expansion or translocation of an immune response from the gastrointestinal tract, or it can be a separate inflammatory event. Alternatively, it may share a common environment or genetic susceptibility with IBD [3]. 

For individuals with IBD, EIMs can affect multiple systems such as the skin, the musculoskeletal system, the hepatic system, and the eyes. The prevalence of EIMs in IBD patients ranges from 38 to 41%, with a higher susceptibility observed in individuals with CD than those with UC [4, 5]. According to the Swiss Inflammatory Bowel Disease Cohort Study (SIBDCS), an IBD cohort study focusing on EIMs, it is accepted that approximately 25% of individuals with IBD could experience multiple EIMs, possibly up to 5 types [6]. EIMs greatly affect the life quality of individuals with IBD, adding to the disease’s overall burden and significantly influencing the mortality rates among patients. Therefore, a comprehensive understanding of the pathogenic factors of EIMs is crucial with the aim of effectively tailoring therapeutic strategies that address all facets of IBD with EIMs.

Recently, the pathophysiology of EIMs in IBD has gained great interest. Some studies have proposed that the pathogenesis of the EIMs may originate in either translocation or extension of the immune response in the intestine, a relatively independent inflammatory process, or a shared environment or genetic susceptibility with IBD [7]. Certain EIMs, like oral aphthous ulcers, erythema nodosum (EN) and episcleritis, may be directly associated with bowel diseases activity, while associations with primary sclerosing cholangitis (PSC) and pyoderma gangrenosum remain uncertain [8]. Furthermore, according to recent genome-wide association studies (GWAS), it is recognized that there is a significant genetic overlap between EIMs and IBD [9]. For instance, there is a significant genetic association between EN and susceptibility variants of IBD, such as ITGAL, PTGER4, CD207, SOCS5, and ITGB3 [10]. Additionally, IBD risk variants, such as BCL2L11, UBASH3A, FOXO1, IRF8, JAK2, STAT3, SOCS1, and TYK2, have been identified in patients with PSC [11]. Moreover, environment, immune system status, microbiota, and microbial products are also believed to contribute to the pathogenic process. Despite extensive research conducted in this field, establishing causal inferences based on these studies is difficult to ascribe to the potential influence of reverse causality, confounding, or measurement error [12]. Furthermore, the implementation of randomized clinical trials (RCTs) presents numerous challenges, including high costs, the need for substantial human resources, time-intensive procedures, and ethical limitations, which hinder their execution. As a substitute, Mendelian Randomization (MR) provides a more feasible approach to testing causality between exposure and outcome, mimicking the design of RCTs.

Given the fixed and randomly assigned nature of genetic variants at conception, MR analyses offer a feasible approach for testing causality between exposure and outcome, resembling RCTs by utilizing genetic variants as instrumental variables (IVs) to evaluate causal relationships while minimizing confounding and reverse causation [13]. Additionally, this study’s exposure variables were not affected by traditional factors, for instance, environmental exposures and behaviors, while satisfying the criterion of temporal precedence, indicating causality precedes outcome occurrence. Conducting a two-sample MR analysis involves obtaining IVs associated with the exposure and outcome from separate population datasets, thereby enhancing statistical power. Hence, to evaluate the likely causal impacts of genetically anticipated IBD, UC and CD on EIMs risk, we implemented a two-sample MR analysis using recently published GWAS data.

## Methods

### Study overview

**Fig. 1:**
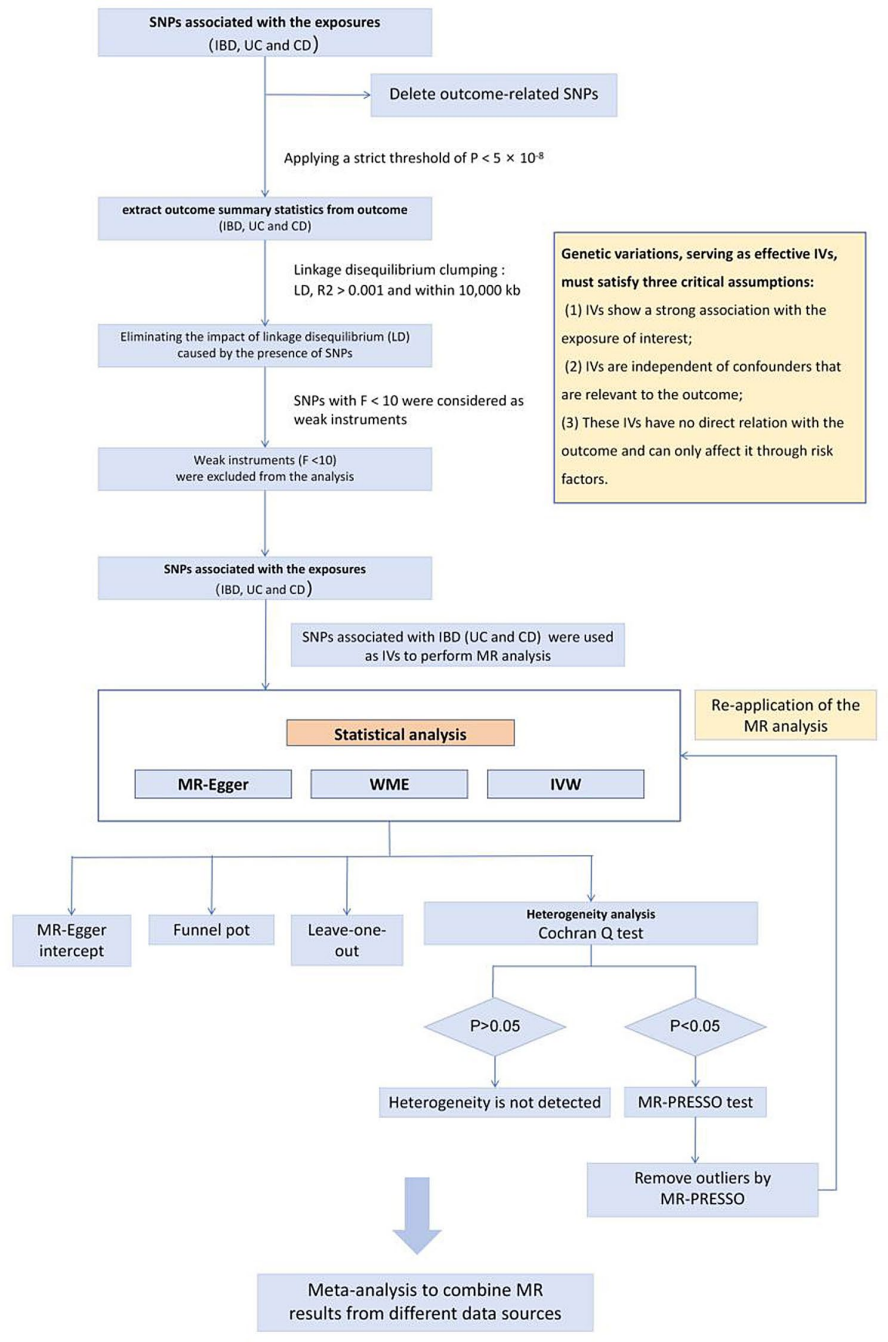
Flowchart of the Mendelian randomization (MR) analysis for inflammatory bowel disease (IBD), ulcerative colitis (UC), Crohn’s disease (CD), and six extraintestinal manifestations (EIMs). This figure outlines the step-by-step workflow of the MR analysis. Key stages, such as instrument selection, causality testing, and sensitivity analysis, are illustrated to provide a clear overview of the analytical process

We executed a two-sample MR analysis using GWAS summary data to assess the causal impacts of genetically anticipated IBD on EIMs. In addition, to further investigate differences between subtypes, we conducted separate analyses for UC and CD. This approach allowed us to explore potential heterogeneity in the causal relationships between each IBD subtype and specific EIMs. EIMs primarily affect the skin (e.g., EN and pyoderma gangrenosum), eyes (e.g., scleritis, episcleritis, and uveitis), the hepatobiliary system (e.g., PSC), and the musculoskeletal system (e.g., spondyloarthritis and enthesitis) [1, 14]. Integrating GWAS results and literature reports, we selected six kinds of EIMs as our outcomes, including EN, episcleritis, scleritis, uveitis, PSC, and spondyloarthritis. To mitigate the risk of sample overlap between exposure and outcome, and to minimize potential bias arising from racial differences, both exposure and outcome GWAS data were obtained from distinct cohorts within the European population [15]. Figure 1 presents a flowchart illustrating the analytical methods employed and outlines the process of conducting the MR analysis.

### Exposure and outcome data sources

The diagnosis of IBD, UC and CD is multifaceted, involving clinical, biochemical, endoscopic, radiological, and histological investigations. Histological evaluation further aids in the diagnosis, with distinct features for UC and CD. Laboratory investigations play a crucial role in the diagnostic workup, with a full blood count, inflammatory markers (e.g. C-reactive protein), electrolytes, liver enzymes, and stool samples for microbiological analysis being standard assessments. For UC, the presence of mucosal inflammation and architectural distortion is typical, while CD may show transmural inflammation, granulomas, and focal inflammation. The SNPs linked to IBD, encompassing both UC and CD, were obtained from the comprehensive summary data of the largest GWAS carried out so far, which were sourced from the International IBD Genetics Consortium (IIBDGC), which encompassed 12,882 cases of IBD and 21,770 individuals without the condition, 6,968 cases of UC and 20,464 controls, as well as 5,956 cases of CD and 14,927 controls [16]. To increase the credibility of this research, we included summary statistics from the extended cohort for IBD (38,565 cases and 37,747 controls), along with 10,920 UC cases and 15,977 controls, and 14,763 CD cases and 15,977 controls for replication [17]. 

Erythema nodosum typically manifests as tender, raised subcutaneous nodules on the lower limbs, with a predilection for females over males. These nodules, which are red or purple in color, range from 1 to 5 centimeters in diameter. Episcleritis is characterized by symptoms such as ocular burning, irritation, pain, and redness. It is crucial to clinically distinguish episcleritis from scleritis, as the latter represents a more serious condition. Scleritis is marked by severe ocular pain and tenderness, which can be indicative of a more profound inflammatory process affecting the sclera. Uveitis can present with a range of symptoms including pain, redness, photophobia, and changes in vision. The nature of the inflammation (e.g., anterior, intermediate, posterior, or panuveitis) can guide the diagnostic process. Spondyloarthritis often presents with inflammatory back pain (IBP), which can be assessed using criteria such as insidious onset, age at onset under 40 years, duration of back pain over 3 months, morning stiffness, and improvement with exercise. Following the integration of GWAS results and literature reports, SNPs associated with EIMs were identified. GWAS data for four common types of EIMs—erythema nodosum (433 cases), episcleritis (660 cases), scleritis (121 cases), and spondyloarthritis (3,037 cases)—were retrieved from the Finngen database (https://www.finngen.fi/en/access_results). Additionally, the uveitis-associated SNPs were accessed from the GWAS study with the data from UK Biobank and FinnGen [18]. In addition, the summary statistics for the phenotypic exposures and outcomes and proportions, are provided in detail in the supplementary materials.

PSC is characterized by inflammation and fibrosis affecting both the intra- and extrahepatic biliary tracts. It often presents with symptoms such as right upper quadrant (RUQ) pain, fever, fatigue, jaundice, pruritus, and weight loss. Hepatic function tests exhibit a cholestatic pattern, indicative of impaired bile flow. Magnetic resonance cholangiography (MRC) is a diagnostic imaging technique that reveals the signature ‘beads-on-a-string’ appearance in PSC, showcasing multiple segmental bile duct strictures and dilatations, which are pathognomonic for the disease. SNPs related to PSC were obtained from the International PSC Study Group (IPSCSG), encompassing a cohort of 4,796 cases and 19,955 controls [19]. 

As our study solely relies on publicly accessible GWAS summary statistics and does not involve the identification of individual-level data, ethical approval was not pursued. According to relevant GWAS databases and reports, researchers typically implemented a series of rigorous quality control steps to ensure data reliability and validity. First, the completeness of sample data was assessed by calculating the amount and distribution of missing values. Samples with a missing rate exceeding 5% were flagged and excluded to minimize data noise and analytical bias. Additionally, genetic correlations between samples were computed to identify and remove highly correlated individuals. In terms of genotype quality control, researchers commonly used multiple methods to evaluate data reliability. For instance, SNPs with high missing rates were excluded, and Hardy-Weinberg Equilibrium (HWE) tests were conducted to detect deviations from genetic equilibrium within populations, ensuring the reasonableness of genotype distributions. These measured effectively mitigate potential impacts from technical errors and biological anomalies on data analysis. Table 1 demonstrates the comprehensive information regarding the GWAS data for both the exposure and outcome.

**Table 1:**
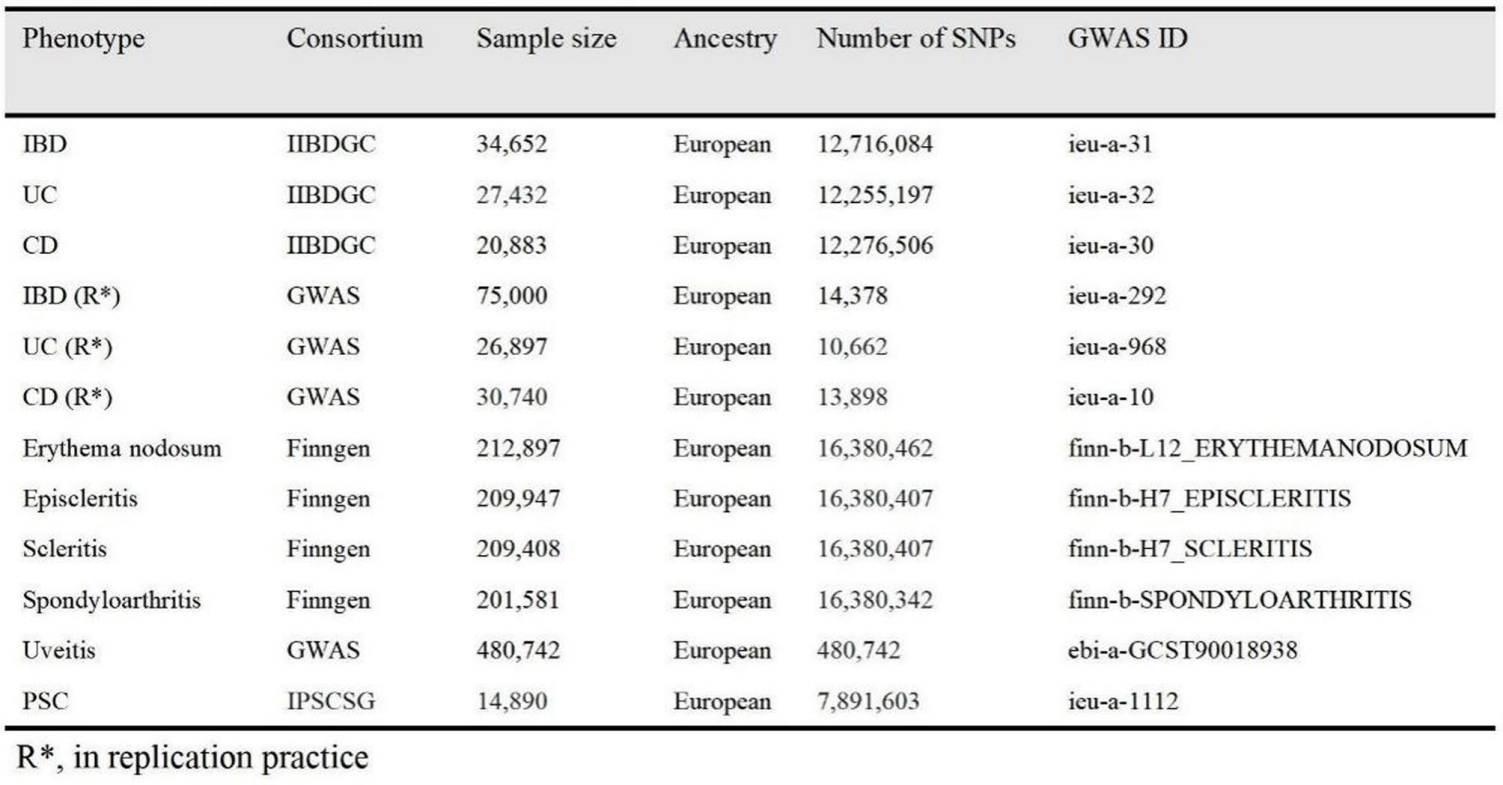
Details of the genome-wide association studies (GWAS) datasets used in this study. This table summarizes the key characteristics of all GWAS datasets included in the analysis. Detailed information is provided to ensure transparency and reproducibility of the study

### Instrumental variable selection

SNPs serving as effective instrumental variables should satisfy 3 critical assumptions: (1) Strong association need to exist between SNPs and the exposure; (2) SNPs should be independent of confounders that are relevant to the outcome; (3) These SNPs should have no direct relation with the outcome and can only affect the outcome through exposure factors [20]. To meet these assumptions, we applied a sequence of quality control measures. Firstly, to extract the SNPs closely related to exposures, we applied a strict threshold of *P* < 5 × 10^− 8^ to determine the significance of these SNPs [21]. Secondly, to avoid linkage disequilibrium (LD) between genetic variants, we employed linkage disequilibrium clumping to confirm the independence of SNPs used in this analysis. Specifically, we set criteria of R^2 < 0.001 and a minimum LD distance of 10,000 kb to determine the independence of SNPs. Thirdly, the application of high-intensity IVs can significantly enhance the accuracy and efficiency of the MR model in estimating causal effects. To reduce the potential bias caused by weak IVs, we assessed the strength of IVs using the F-statistic. If the F-statistic is less than 10, the estimates of causal effects may be severely biased, and IVs should be excluded from the analysis [21]. Additionally, to mitigate potential interference from confounding factors and horizontal pleiotropy, we utilized the PhenoScanner database (http://www.phenoscanner.medschl.cam.ac.uk/phenoscanner) to investigate the associations between the selected IVs and other phenotypes that may pose a risk of influencing the outcome.

### Statistical analyses

To evaluate the likely causal relationship between IBD, including UC and CD with six EIMs, we employed random-effects inverse variance weighting (IVW), MR-Egger, and weight median estimator (WME) for MR analysis. The results conducted by utilizing the IVW method stand as the main estimates for MR analysis due to its ability to provide reliable causal estimates, particularly when there is no presence of directional pleiotropy [22]. The results of the WME and MR-Egger tests can be complementary with the IVW method for more comprehensive assessments. Additionally, a serious of sensitivity analyses, including heterogeneity and pleiotropy, were performed to improve the reliability and stability of the results. We utilized Cochran’s Q test to quantify the heterogeneity of IVs, signifying its presence when the p-value (P) was less than 0.05. Upon detecting heterogeneity, MR-PRESSO (NbDistribution = 10000) was utilized to identify specific SNPs contributing to the observed heterogeneity [23]. Subsequently, after removing outliers, reapplication of the MR analysis was necessary. The MR-Egger intercept test was employed to evaluate the potential pleiotropy for the selected IVs. A leave-one-out sensitivity test was performed to examine the significant effect of individual SNPs on the causal effect. Additionally, we employed visualization, including scatter plots and funnel plots, to combine with statistical tests to discern pleiotropic instrumental variables.

Further, the sample size of exposures in the replication practice was significantly larger compared to the initial practice, and this difference may impact the accuracy and efficiency of the MR Model. To mitigate bias from sample differences and enhance the quality and reliability of research outcomes, we employed the fixed-effects meta-analysis to consolidate MR results from both the IVW approach of the initial practice and the replication practice. To assess reverse causality, we conducted additional MR analyses, considering EIMs as the exposure and IBD as the outcome, to evaluate the potential causal effects of EIMs on IBD. It helped us to identify and exclude possible reverse causal relationships, thereby enhancing the credibility of our findings.

The MR analyses were conducted using the TwoSampleMR and MRPRESSO packages within R version 4.1.2 software. The meta-analyses were performed using the “meta” R package. The Forestploter R package was used to draw forest plots.

### Ethical consideration

The data utilized in this study is sourced exclusively from publicly available databases. Hence, there was no need for additional ethical approval.

## Results

After applying stringent selection criteria as previously described, exposure-associated SNPs were identified as IVs for further MR analyses. Comprehensive information regarding the exposure-associated SNPs (IBD, UC, and CD) can be found in the Supplementary Tables 3–8. These SNPs were chosen based on their robust association with the exposure, thereby satisfying the relevance assumption. The F-values of IVs used in the study were significantly greater than 10, which indicated a low possibility of bias due to weak IVs. This threshold helps to mitigate the potential for weak instrument bias and ensures that our IVs are sufficiently predictive of the exposure. The IVW method was used to obtain the primary results from the MR analysis. Supplementary Tables 1 and Supplementary Table 2 contain the results obtained from other MR analysis methods used in the initial and replication practice, respectively. Additionally, the summary statistics for the phenotypic exposures and outcomes and proportions, are provided in detail in the supplementary materials.

### MR analysis for causal impacts of genetically anticipated IBD on EIMs

**Fig. 2:**
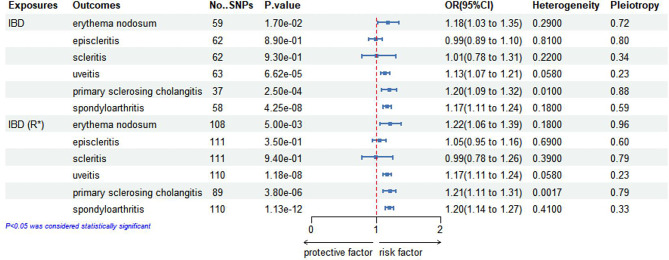
Inverse-variance weighted (IVW) analysis of the causal relationship between IBD and six EIMs. This figure presents the results of the IVW analysis, demonstrating the causal associations of IBD with six EIMs. Effect estimates are shown as beta coefficients with 95% confidence intervals (CIs). Significant associations are highlighted to illustrate the strength and direction of the causal relationships

**Fig. 3:**
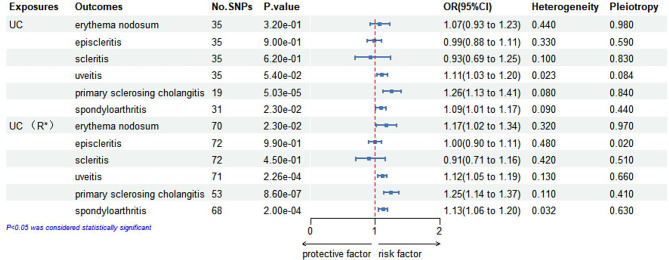
Inverse-variance weighted (IVW) analysis of the causal relationship between UC and six EIMs. This figure visualizes the outcomes of the inverse-variance weighted (IVW) analysis, illustrating the causal connections between UC and six EIMs. The effect estimates, expressed as beta coefficients with 95% CIs, are displayed, and significant causal relationships are prominently marked to reflect their strength and directionality

Figure 2 shows a comparison between the results of the initial practice and repeated practice based on the IVW analysis. As can be seen in Fig. 3, in the initial practice, it appears that genetically anticipated IBD contributes to a causal relationship with increased EN (OR, 1.18; 95%Cl, 1.03–1.35; *p* = 0.017), uveitis (OR, 1.13; 95%Cl, 1.07–1.21; *p* = 6.62*10^− 5^), PSC (OR, 1.20; 95%Cl, 1.09–1.32; *p* = 2.5*10^− 4^) and spondyloarthritis (OR, 1.17; 95%Cl, 1.11–1.24; *p* = 4.25*10^− 8^) susceptibility using IVW method. Furthermore, there is no evidence to indicate a causal relationship between IBD and episcleritis or scleritis. In the replication practice, it provides evidence supporting a causal relationship between IBD and an increased risk of EN, uveitis, PSC, and spondyloarthritis, which aligns with the findings of the initial practice. The results suggested that there is no causal relationship between IBD and episcleritis or scleritis. Additionally, the results of both scatter plots and symmetric funnel plots indicated that no pleiotropy was identified. In addition, the leave-one-out analysis further verified the robustness of our results. The supplementary Figs. 1–6 provide the funnel plots, scatter plots, and leave-one-out analysis for each outcome respectively.

No horizontal pleiotropy effect was detected, which indicated that the results were credible in this study. In addition, heterogeneity was detected in certain MR analyses. We performed the MR-PRESSO outliner test to confirm the specific SNPs led to the heterogeneous results and the information on these outliers is shown in Table 2. After removing these outliers and reapplying the MR analyses, the level of heterogeneity decreased.

**Table 2:**
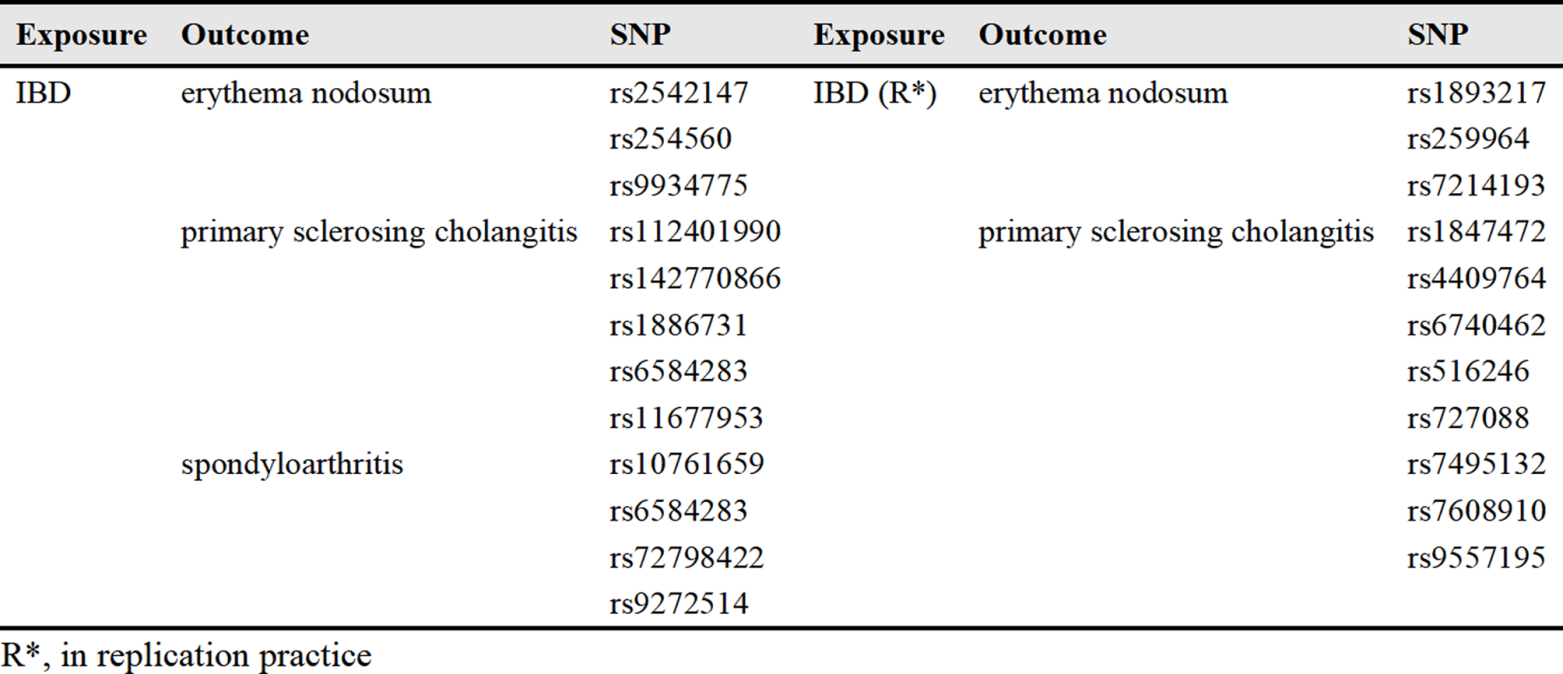
The information of outliers for the causal link of IBD with EIMs

### MR analysis for causal impacts of genetically anticipated UC on EIMs

The results of the IVW analysis for UC and EIMs in the initial and replication practices are presented in Fig. 3. We found evidence of a causal relationship between UC and increased susceptibility to PSC (OR, 1.26; 95% CI, 1.13–1.41; *p* = 0.00005) and spondyloarthritis (OR, 1.08; 95% CI, 1.01–1.17; *p* = 0.011) in the initial practice. Additionally, no causal relationship between UC and EN, uveitis, episcleritis or scleritis was identified. In the replication practice, some of these results were slightly different from those observed in the initial practice. The results from the replication provided evidence of a causal relationship between UC and an increased risk of entities, including EN, uveitis, PSC, and spondyloarthritis. This discrepancy between the initial practice and the replication practice may be attributed to variations in data collected from different sources. In addition, the absence of pleiotropic bias is suggested based on the analysis of scatter plots and symmetric funnel plots, while the robustness of our estimation results is further demonstrated through leave-one-out analysis. Funnel plots, scatter plots, and leave-one-out analysis plots for each outcome in the replication practice were presented in supplementary Figs. 7–12.

Furthermore, heterogeneity was observed in certain analyses, and the information on the outliers was presented in Table 3. Upon removing these specific SNPs and repeating the MR analysis, the heterogeneity became insignificant. (*P* > 0.05). No evidence of horizontal pleiotropy was found in any MR analysis results, indicating the credibility of the study findings.

**Table 3:**
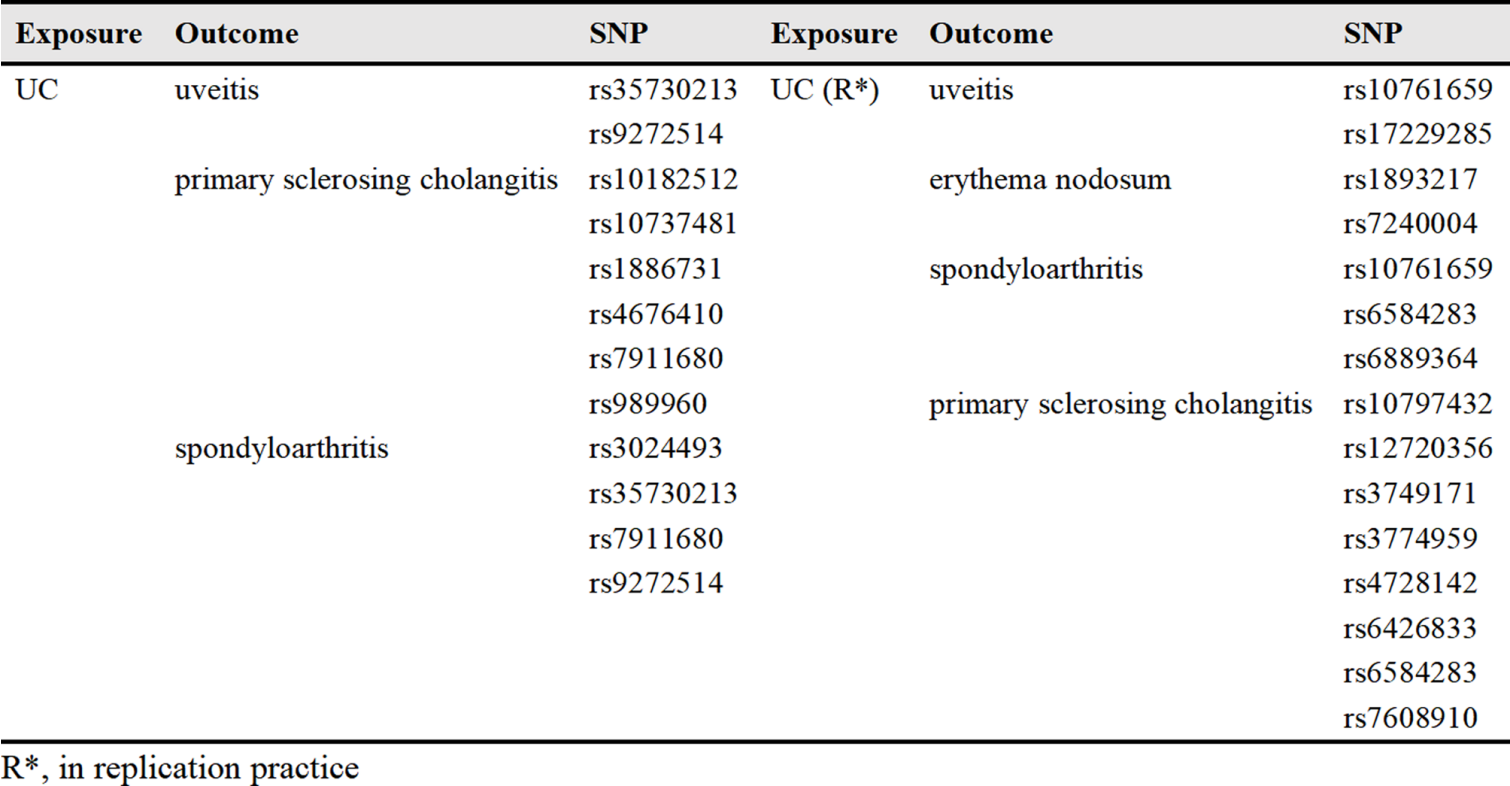
The information of outliers for the causal link of UC with ElMs

### MR analysis for causal impacts of genetically anticipated CD with EIMs

**Fig. 4:**
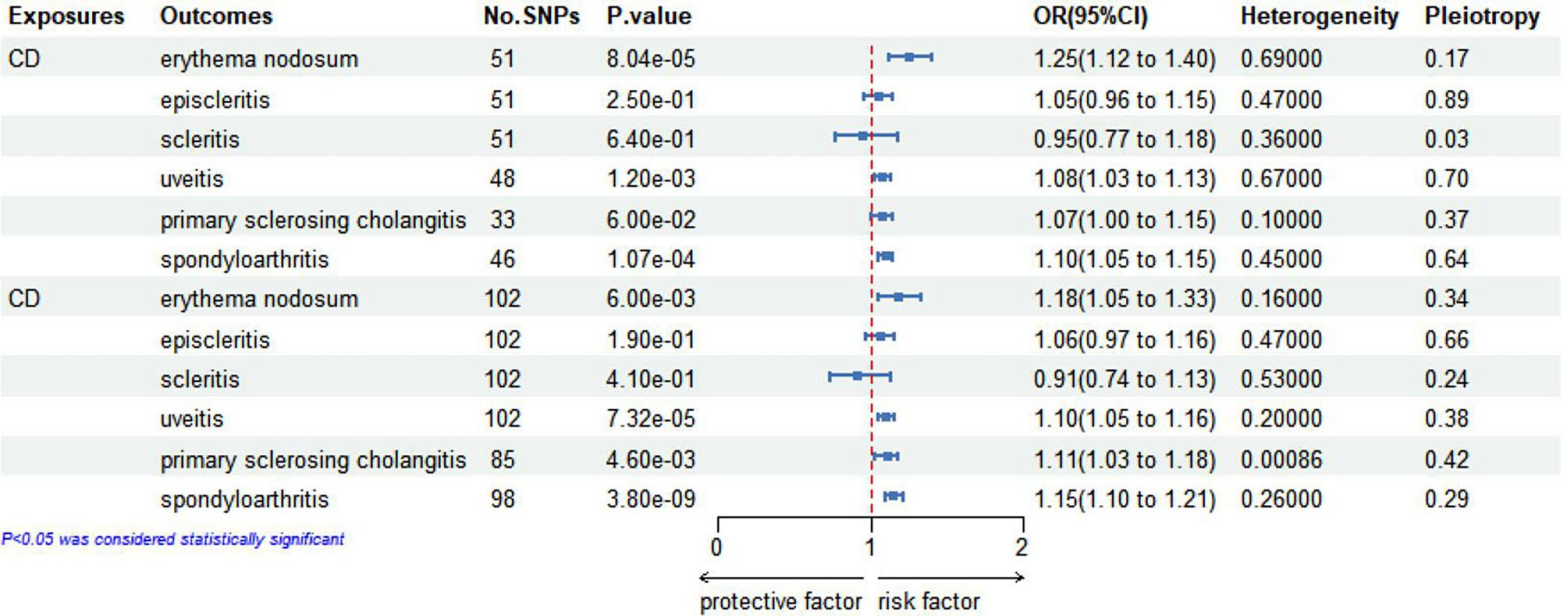
Causal associations between Crohn’s disease (CD) and six EIMs assessed by IVW analysis. This figure displays the results of the IVW analysis, illustrating the causal relationships between CD and six EIMs. Effect estimates are reported as beta coefficients with 95% confidence intervals. Statistically significant associations are highlighted to indicate the strength and direction of the causal effects

The results of the IVW analysis focusing on CD and EIMs in the initial and replication practices are presented in Fig. 4. In the initial practice, we observed the causal impacts of genetically predicted CD on the increased risk of EN (OR, 1.25; 95%Cl, 1.12–1.40; *p* < 0.01), uveitis (OR, 1.08; 95%Cl, 1.03–1.13; *p* < 0.01) and spondyloarthritis (OR, 1.10; 95%Cl, 1.05–1.15; *p* < 0.01) after correcting for multiple testing, and no causal relationships between CD and episcleritis, scleritis or PSC were identified. In the replication practice, comparable research findings were observed. The results showed a causal link between CD and an increase in the risk of PSC, which was not consistent with initial practice. Both scatter and symmetric funnel plots confirmed that there was no pleiotropic bias present. Additionally, it is believed that the results are stable according to the leave-one-out analysis plots. The supplementary Figs. 13–18 provide the funnel plots, scatter plots, and leave-one-out analysis plots for each outcome respectively.

In addition, certain MR analyses showed heterogeneity and the information on the outliers was presented in Table 4. After removing these outliers, the MR analysis was repeated and the results showed that heterogeneity was no longer significant. The results of all analyses did not show pleiotropy, suggesting that the results of this analysis were reliable.

**Table 4:**
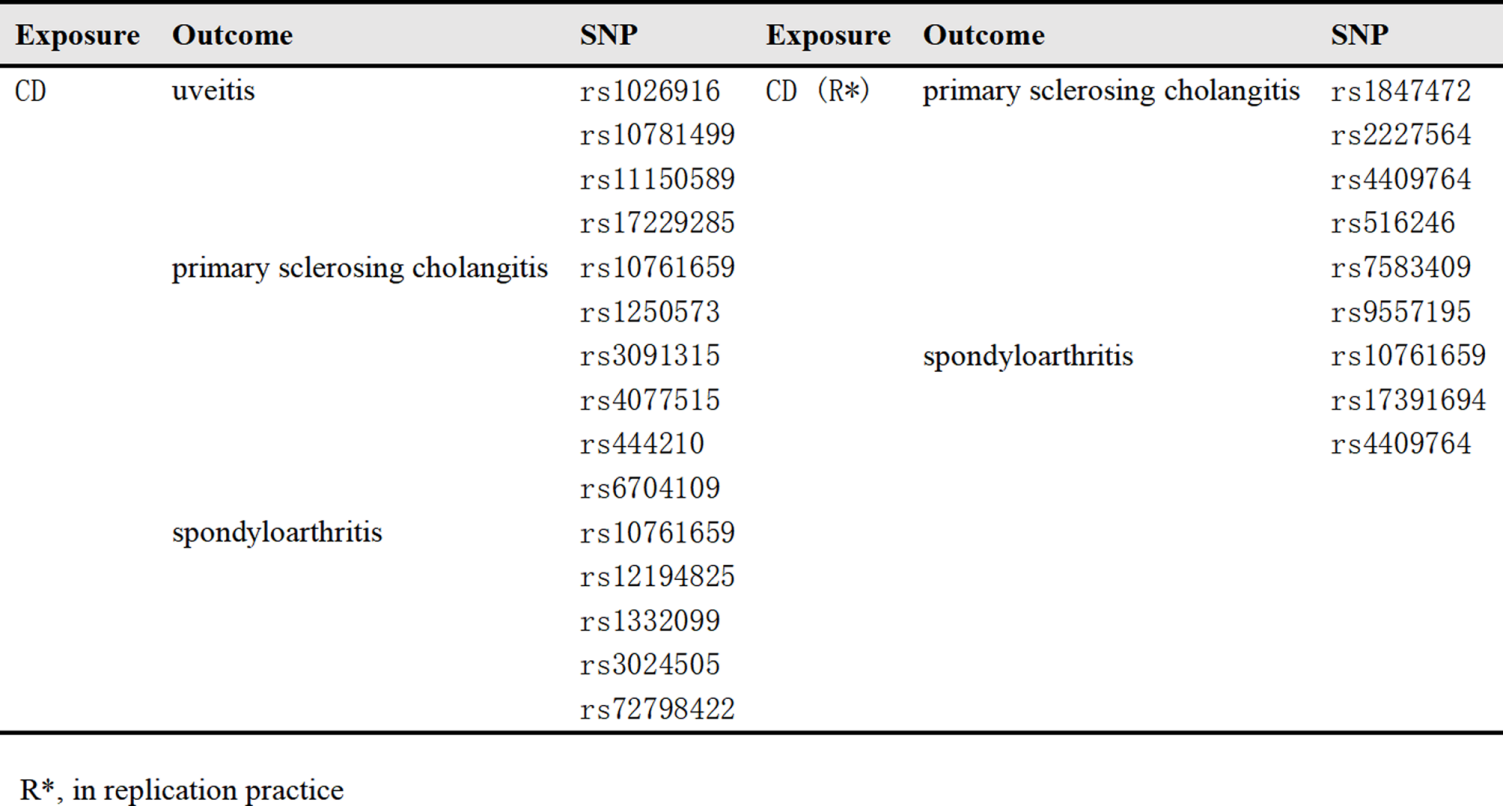
The information of outliers for the causal link of CD with ElMs

### Meta-analysis to combine MR results obtained from different data sources

**Fig. 5:**
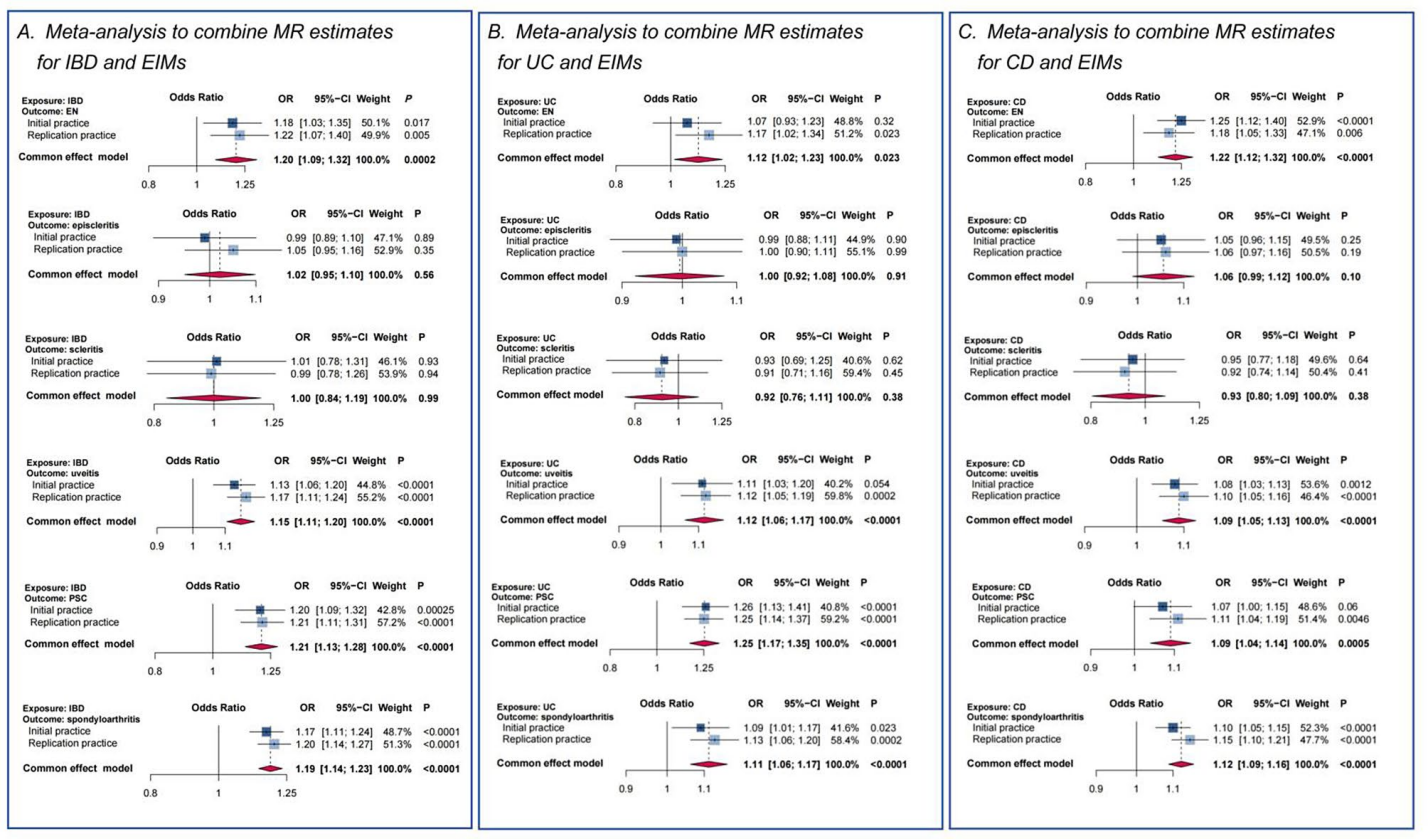
Meta-analysis of Mendelian randomization (MR) results across multiple data sources

The meta-analysis results are presented in Fig. 5. The combined results provided evidence of a causal relationship between genetically anticipated IBD and increased risks of entities, including EN (OR, 1.20; 95% CI, 1.09–1.32; *p* < 0.01), uveitis (OR, 1.15; 95% CI, 1.11–1.20; *p* < 0.01), PSC (OR, 1.21; 95% CI, 1.13–1.28; *p* < 0.01), and spondyloarthritis (OR, 1.19; 95% CI, 1.14–1.23; *p* < 0.0001).

In subgroup analyses, the results indicated an causal relationship between UC and increased risks of entities, including EN (OR, 1.12; 95%Cl, 1.02–1.23; *p* = 0.0228), uveitis (OR, 1.11; 95%Cl, 1.06–1.17; *p* < 0.0001), PSC (OR, 1.25; 95%Cl, 1.17–1.35; *p* < 0.0001), and spondyloarthritis (OR, 1.11; 95%Cl, 1.06–1.17; *p* < 0.0001). In addition, genetically predicted CD showed a causal relationship with increased risks of EN (OR, 1.22; 95% CI, 1.12–1.32; *p* < 0.0001), uveitis (OR, 1.09; 95% CI, 1.05–1.13; *p* < 0.0001), PSC (OR, 1.12; 95% CI, 1.04–1.14; *p* < 0.0001), and spondyloarthritis (OR, 1.11; 95% CI, 1.09–1.16; *p* < 0.0001).

The most surprising aspect of the results was that no causal effect of IBD on episcleritis or scleritis was found. In the subgroup analyses, no causal relationship could be established between UC and CD and the occurrence of episcleritis or scleritis.

### MR analysis for causal impacts of genetically anticipated EIMs with IBD, UC and CD

**Fig. 6:**
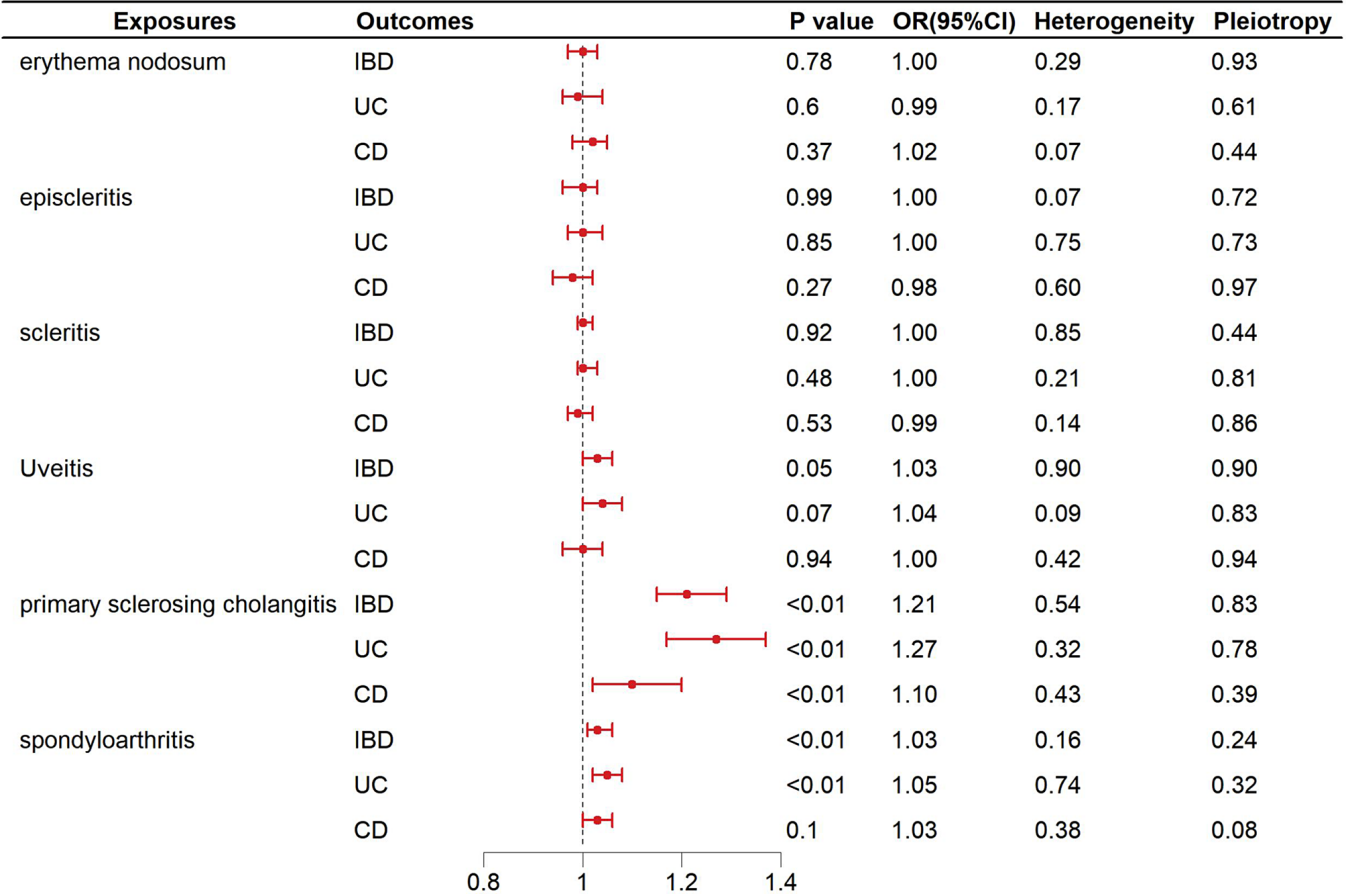
Associations between EIMs and IBD, including UC and CD, assessed by IVW analysis. The results display the outcomes of the IVW analysis which indicate the association between EIMs and IBD, comprising UC and CD. Effect estimates are reported as beta coefficients with 95% CIs. Statistically significant associations are highlighted to illustrate the strength and direction of the causal effects

The results of the IVW analysis focusing on EIMs and IBD, comprising UC and CD, are presented in Fig. 6. The results of MR studies provided evidence of a causal relationship between genetically predicted PSC and an elevated risk of IBD (OR, 1.21; 95%Cl, 1.15–1.29; *p* < 0.01), UC (OR, 1.27; 95%Cl, 1.17–1.37; *p* < 0.01) and CD (OR, 1.10; 95%Cl, 1.02–1.20; *p* < 0.01). In addition, the results demonstrated a causal relationship between spondyloarthritis and an increased risk of both IBD (OR, 1.03; 95% CI, 1.01–1.06; *p* < 0.01) and UC (OR, 1.05; 95% CI, 1.02–1.08; *p* < 0.01).

The study employed several sensitivity analyses to assess the robustness of the findings. MR-Egger regression was used to detect horizontal pleiotropy, and no significant pleiotropy was observed in this study. Additionally, heterogeneity tests were performed. After removing these outliers, the MR analysis was repeated and the results showed that heterogeneity was no longer significant. The supplementary Figs. 19–24 provide the funnel plots, scatter plots, and leave-one-out analysis MR analysis for causal impacts of genetically anticipated EIMs with IBD, UC and CD.

## Discussion

Some epidemiological and genetic studies have demonstrated the connection between IBD and EIMs. Nevertheless, the vast majority of disease variants occur in regulatory areas that are out of protein-coding regions, interpreting their functional implications may be a challenge. Additionally, the complexities of reverse causation and confounding factors make it inherently arduous to establish causal inferences and may introduce potential biases [24]. The MR approach offers a solution by employing SNPs which exhibit a robust link to the exposure as IVs, allowing for causal inference while mitigating confounding from various sources.

**Fig. 7:**
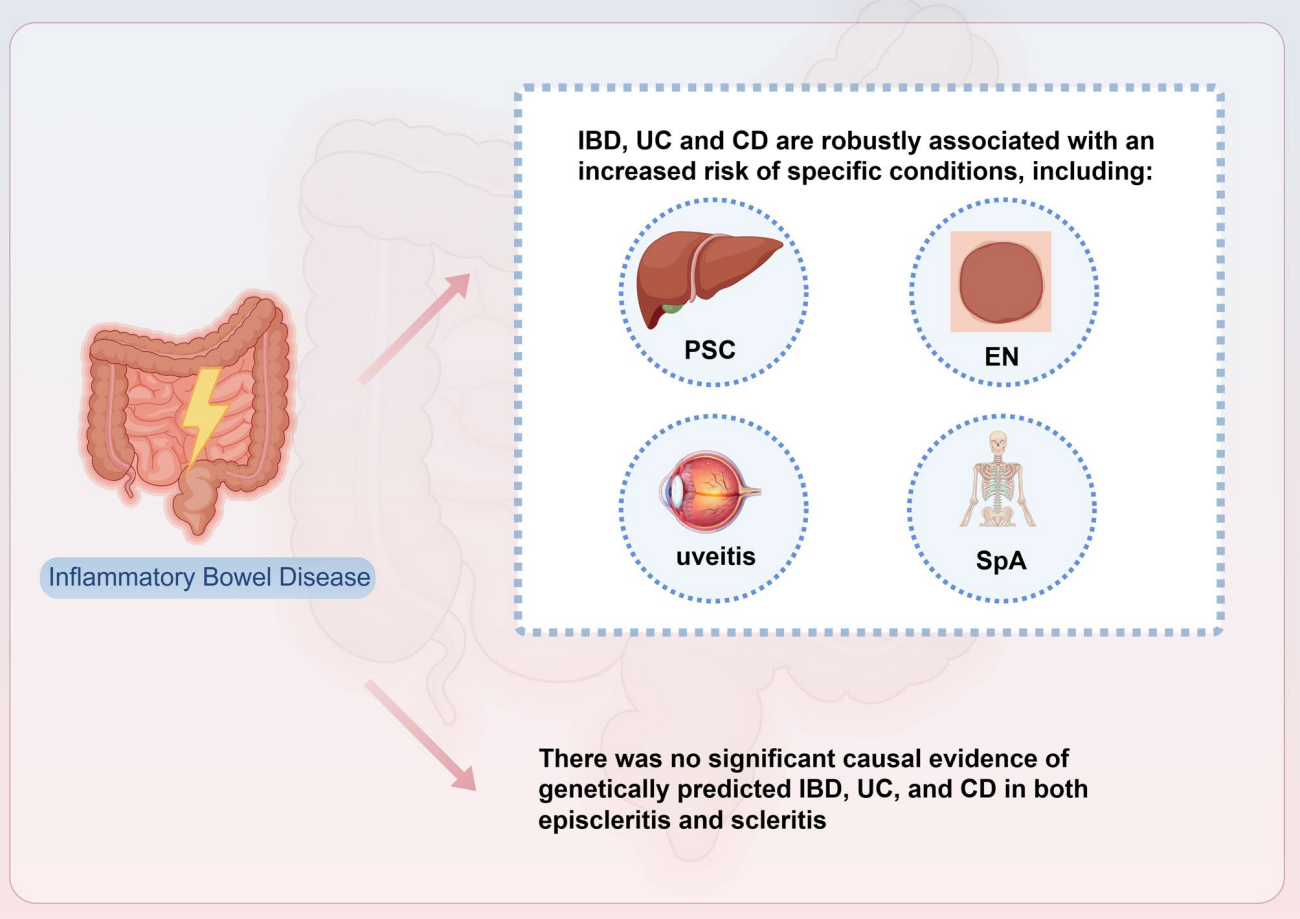
The visual representation of the key findings in this study. This figure provides a comprehensive overview of the main results, highlighting the relationships and significant outcomes identified in the analysis

This study provides compelling evidence of a causal relationship between genetically predicted IBD, encompassing UC and CD, and an increased risk of developing specific EIMs, including EN, uveitis, PSC, and spondyloarthritis. Additionally, no significant causal relationship was observed between IBD, UC, and CD, and the incidence of either episcleritis or scleritis (Fig. 7). Moreover, reverse causality analyses revealed that certain EIMs, particularly PSC and spondyloarthritis, have a causal relationship with an elevated risk of IBD, UC, and CD. These findings highlight a bidirectional causal relationship between IBD and certain EIMs, suggesting that monitoring these conditions may play a crucial role in early detection and management strategies for IBD patients. Further exploration of these causal pathways could contribute to better clinical outcomes by providing a deeper understanding of the interconnection between IBD and its extraintestinal effects.

Numerous recent studies have indicated that individuals with IBD carrying specific HLA alleles are at an elevated risk of developing EIMs. For CD patients, the presence of HLA-DR1, HLA-A2, and HLA-DQw5 alleles is linked to EIMs, while in UC, this heightened risk is related to HLA-B27, HLA-B58, and HLA-DR103 [8]. Among these, HLA-B27 is recognized as a well-established risk factor for the progression of EIMs. Its correlation has been observed in the context of joint, skin, and ophthalmologic manifestations [25]. Recently, some GWAS studies have found that some EIMs are associated with risk loci for IBD, highlighting the genetic overlap between these conditions. However, this overlap was not observed between IBD and PSC, illustrating immunogenetic distinct features of PSC compared to IBD [26]. 

Musculoskeletal EIMs confirmed by the skeletal radiological criteria are one of the commonly observed manifestations, occurring in approximately 46% of IBD patients, and spondyloarthritis can occur in up to 25%.^1^ While the pathogenesis of spondyloarthritis has been extensively studied, the exact etiology and the mechanism remain under investigation. Although it has been suggested that the correlation between HLA and IBD-associated SpA is comparatively less obvious than other kinds of SpA, it is still evident that genetics could play a significant role in this condition, particularly the HLA-B27 gene [27, 28]. Several studies have shown that the presence of certain HLA alleles, such as HLA-B27 and HLA-DR1, contributes to the increased risk of developing musculoskeletal manifestations in IBD [29], and the interaction between bacteria and HLA-B27 alleles is considered significant in the pathogenesis of spondyloarthritis [30]. In addition, the “gut-synovial axis” hypothesis suggests that activated Th1 and Th17 cells in the intestine of IBD patients can migrate to synovial tissue, causing joint inflammation [31]. 

PSC, the most common hepato-biliary EIMs in IBD, may affect 60–80% of IBD patients [32] The prevalence of PSC can reach up to 5% in UC patients and lower in CD patients [33, 34]. Colonic autoantibodies from PSC patients show cross-reactivity with biliary epithelium, highlighting the gut-liver axis’s role in PSC pathogenesis [35]. The gut microbiota, essential for nutrient absorption and immune regulation, is implicated in PSC development via the gut-liver axis [36]. Individuals with IBD exhibit reduced diversity in their gut microbiota, which increases relative abundances of potential enteric pathogenic bacteria, which will promote the migration of intestinal lymphocytes and facilitate bacteria and their products to move into the portal venous circulation through compromised mucosa [37, 38]. Another hypothesis suggests that anti-neutrophil cytoplasmic antibodies (P-ANCAs) may cross-react with colonic antigens in susceptible individuals, leading to abnormal immunoreactions and biliary inflammation [39]. Furthermore, the GPBAR1 gene, responsible for encoding a G-protein involved in the absorption of bile salts, demonstrates elevated expression levels in both the ileum and colon, demonstrating a significant association with PSC [40]. 

Studies have reported the incidence of EN ranging from 5 to 15% in individuals with CD, and from 2 to 10% in individuals with UC [14]. Some known IBD susceptibility genes, such as ITGAL, CD207, and ITGB3, are associated with EN [10]. Previous studies have indicated an overexpression of genes encoding Th1 cytokines in the skin lesions of EN patients. Furthermore, the content of Th1 cytokines, including IFN-γ and IL-12, is significantly increased in the skin lesions as well as in the serum. These findings suggest that the Th1 cellular immune response may contribute to the pathogenesis of skin lesions of patients with EN [41]. Besides, adhesion molecules and inflammatory mediators, like E-selectin, P-selectin, and platelet endothelial cell adhesion molecule-1, may account for the pathogenesis of EN [42]. Additionally, deposition of immunocomplex has been observed around veins in the subcutaneous adipose connective tissue, suggesting the role of immune-complex reactions in the pathophysiology of EN [43]. Anti-tumor necrosis factor (anti-TNF) medications have shown significant effectiveness in treating cutaneous EIMs, suggesting a potential shared pathogenic link involving TNF between EIM and IBD. Additionally, a previous study has discovered that the TNF-NFκB pathway was upregulated in samples from patients with EN and pyoderma gangrenosum [44]. 

Between 4% and 12% of patients with IBD experience ocular manifestations, with these ocular EIMs being more prevalent in CD compared with UC [45, 46]. The primary ocular manifestation observed in the majority of IBD patients is episcleritis [47]. Moreover, some exceptionally severe types, such as scleritis, are relatively uncommon. Research indicated that there exists a robust association between ocular inflammation and HLA alleles, including HLA-B27, B58, and HLA-DRB1 [47]. The unanticipated outcomes reported by this study indicate no evidence supporting a causal connection between IBD, including UC and CD, and the occurrence of episcleritis or scleritis. However, a strong causal relationship was observed with uveitis. This result has further strengthened our confidence that pathogenic mechanisms of episcleritis, scleritis and uveitis are different, although they are common ocular EIMs.

The immune system is believed to play a crucial role in the pathogenesis of scleritis and episcleritis [48]. It is characterized by the infiltration of immune cells such as B cells and macrophages, which suggests a role for cell-mediated immunity in the disease process [49]. In addition, in tissues of certain individuals with scleritis, the presence of immune complexes, such as those formed by protease 3 (PR3) antibodies, can trigger complement responses and lead to vasculitis [49]. Moreover, matrix metalloproteinases (MMPs) contributes to scleral destruction, and their production can be induced by TNF-alpha, which is found in the tissues of some scleritis patients [50]. A previous study demonstrated that certain genes, such as cytotoxic T lymphocyte-associated antigen-4 (CTLA4) and protein tyrosine phosphatase non-receptor type 22 (PTPN22), have the ability to regulate T lymphocyte activity. It is accepted that polymorphisms in these genes could contribute to the development of autoimmune responses [51]. For uveitis, the pathogenesis is associated with the activation of Th1 and Th17 cells, which secrete inflammatory cytokines such as TNF-alpha, IL-17, IL-23, and IL-6, driving the inflammatory response [52]. The specific bacterial species can influence the development of distinct T cell populations, including Th17 cells and IL-17, which contribute to inflammation [53]. Certain research has indicated that the composition of the microbiome in the gut differs in the absence of disease susceptibility alleles associated with HLA [54]. Moreover, research has confirmed that labeled leukocytes are capable of migrating from the intestine to the eye, substantiating a direct correlation between the gut and the eye [55, 56]. In summary, while both scleritis and uveitis involve immune-mediated mechanisms, scleritis is characterized by cells, antibody and complement-mediated processes, and matrix metalloproteinase activity. And uveitis is driven by Th1 and Th17 cell activation, gut microbiota interactions, and the gut-eye axis.

When diagnosing and managing patients with EIMs, it is of great significance for the medical staff to pay attention to the causal relationship between IBD and EIMs. For certain EIMs which are the consequence of IBD, such as EN, PSC, uveitis, and spondyloarthritis, the proper management of IBD can potentially alleviate the burden of EIMs, positively impact the management and treatment of EIMs, and improve the life quality of EIMs patients.

However, several limitations of this MR investigation should be taken into account. First, the SNPs statistics about the IBD, UC and CD we used were from a population of Europeans. The majority of SNPs of EIMs were derived from FinnGen databases. Although numerous published MR studies typically treat Finnish data as part of a broader European cohort, the genetic distinctions of the Finnish population do not fully address when interpreting results. Given the unique genetic profile of the Finnish population—marked by historical bottlenecks and relative genetic isolation—the inclusion of Finnish data among “European” samples may introduce population-specific biases that could impact generalizability. This is particularly relevant as the genetic diversity within Europe is substantial, and specific regional groups, like the Finnish, may differ genetically from other European populations. With the conduction of future GWAS studies on a larger scale, researchers will gain access to a greater multitude of IVs that fulfill the significance threshold required for conducting MR analysis with SNPs. This will enhance the reliability of the results obtained. Secondly, this study was conducted based on GWAS data from European populations. Due to differences in genetic structures between different ancestry, results based on specific populations may not be directly applicable to other populations. To confirm the generalizability of these genetic variants, future studies should be conducted in different racial groups. Thirdly, although multiple sensitivity analyses were performed, further research is needed to investigate whether each SNP locus serving as IVs satisfies three critical assumptions of MR analysis. Additionally, although we employed the MR-PRESSO outlier test and removed outliers upon detecting heterogeneity, statistical heterogeneity persists among certain instrumentally determined IV estimates, necessitating further discussion. Lastly, MR analyses are often limited to cross-sectional data, which restricts our ability to capture dynamic, time-dependent relationships between exposures and outcomes. Future longitudinal studies may provide insights into the temporal aspects of these associations, which would strengthen the causal interpretations derived from MR.

## Electronic Supplementary Material

Below is the link to the electronic supplementary material.

Supplementary Material 1

Supplementary Material 2

Supplementary Material 3

Supplementary Material 4

Supplementary Material 5

Supplementary Material 6

Supplementary Material 7

Supplementary Material 8

Supplementary Material 9

Supplementary Material 10

Supplementary Material 11

## Data Availability

The study’s datasets are publicly available. The research paper includes the original contributions, which can be found in both the article and supplementary materials. For any further inquiries please contact the corresponding authors.

## Abbreviations

IBD: Inflammatory bowel disease
UC: Ulcerative colitis
CD: Crohn’s disease
EIMs: Extraintestinal manifestations
EN: Erythema nodosum
PSC: Primary sclerosing cholangitis
SNPs: Single nucleotide polymorphisms
GWAS: Genome-wide association studies
MR: Mendelian randomization
IVs: Instrumental variables
LD: Linkage disequilibrium
IVW: Inverse variance weighting
WME: Weight median estimator
anti-TNF: Anti-tumor necrosis factor
PR3: Protease 3
MMPs: Matrix metalloproteinases
CTLA4: Cytotoxic T lymphocyte-associated antigen-4
PTPN22: Protein tyrosine phosphatase non-receptor type 22
IIBDGC: International IBD Genetics Consortium
IPSCSG: International PSC Study Group
HWE: Hardy-Weinberg Equilibrium
