# Supplementary material for: The causal impact of genetically predicted inflammatory bowel disease on extraintestinal manifestations: a mendelian randomization study
Source: BMC Gastroenterol. 2025 Mar 4;25:135. doi: 10.1186/s12876-024-03566-4 (PMC11881308; doi:10.1186/s12876-024-03566-4)
Supplement: Supplementary file 1 — Supplementary Material 1 [file 12876_2024_3566_MOESM1_ESM.pdf]

STROBE-MR checklist of recommended items to address in reports of Mendelian randomization studies[1 2]

Item No.	Section	Checklist item	Page No.	Relevant text from manuscript
1	**TITLE and ABSTRACT**	Indicate Mendelian randomization (MR) as the study's design in the title and/or the abstract if that is a main purpose of the study	1-4	**Title:** The Causal Impact of Genetically Predicted Inflammatory Bowel Disease on Extraintestinal Manifestations: a Mendelian Randomization Study<br>**Abstract:**<br>**Background:** Extraintestinal manifestations (EIMs) significantly affect the life quality of people with inflammatory bowel disease (IBD) and are crucial factors impacting occurrence rates and mortality among IBD patients. This study performed a Mendelian randomization (MR) analysis to investigate the causal relationships between genetically predicted IBD and the development of EIMs, including erythema nodosum (EN), episcleritis, scleritis, uveitis, primary sclerosing cholangitis (PSC), and spondyloarthritis. To further investigate differences between subtypes, separate analyses were conducted for ulcerative colitis (UC) and Crohn's disease (CD).<br>**Methods:** The study was conducted based on genome-wide association studies (GWAS) data. We carefully selected SNPs associated with both exposure and outcome by comparing and integrating data from GWAS and relevant literature, and prioritizing studies with large sample sizes, high quality, and as much population homogeneity as possible. The SNPs associated with IBD,UC and CD were extracted from the International Inflammatory Bowel Disease Genetics Consortium. And the SNPs associated with EIMs were extracted from the UK Biobank, the International PSC Study Group and the FinnGen study. A series of quality control steps were taken in our analysis to select eligible instrumental SNPs which were strongly associated with exposure. The causal effects were estimated using a primary analysis that employed inverse-variance weighting (IVW) and complementary analysis that utilized MR-Egger weighted by the median. A sensitivity analysis was conducted using the Cochran Q statistic, a funnel plot, the MR-Egger intercept, and a leave-one-out approach. Reverse causality analysis was also performed to ensure the robustness of the findings. Furthermore, a fixed-effects meta-analysis was employed to combine MR outcomes from various data origins, bolstering the strength and dependability of our findings.<br>**Results:** Our findings indicated that genetically predicted IBD had a robust causal relationship with an increased risk of specific conditions, including EN (OR, 1.20; 95% CI, 1.09-1.32; p<0.01), uveitis (OR, 1.15; 95% CI, 1.11-1.20; p<0.01), PSC (OR, 1.21; 95% CI, 1.13-1.28; p<0.01), and spondyloarthritis (OR, 1.19; 95% CI, 1.14-1.23; p<0.01). In subgroup analyses, the causal effects of both UC and CD on EN, uveitis, PSC, and spondyloarthritis were also significant and robust. Additionally, no significant evidence of causality was observed between genetically

				predicted IBD, UC, and CD, and the occurrence of both episcleritis and scleritis. The results of reverse causality analysis indicated a robust causal association between genetically predicted PSC and the elevated risk of IBD (OR, 1.21; 95% CI, 1.15-1.29; p<0.01), UC (OR, 1.27; 95% CI, 1.17-1.37; p<0.01), and CD (OR, 1.10; 95% CI, 1.02-1.20; p<0.01). Additionally, spondyloarthritis had a causal relationship with an increased risk of both IBD (OR, 1.03; 95% CI, 1.01-1.06; p<0.01) and UC (OR, 1.05; 95% CI, 1.02-1.08; p<0.01).
	**INTRODUCTION**		6-8	
2	**Background**	Explain the scientific background and rationale for the reported study. What is the exposure? Is a potential causal relationship between exposure and outcome plausible? Justify why MR is a helpful method to address the study question	6-8	Inflammatory bowel disease (IBD), which comprises Crohn's disease (CD) and ulcerative colitis (UC), is a chronic, systemic, autoimmune disease that can not only affect the alimentary canal but also could impact the function of other organs. Typically, when organs outside the alimentary tract are affected, we refer to this as extraintestinal manifestations (EIMs) of IBD. EIMs need to be distinguished from extra-intestinal complications of IBD, which are consequences of intestinal inflammation either directly or indirectly. The European Crohn's and Colitis Organization (ECCO) offers a comprehensive description of EIMs. It is an inflammatory pathology affecting extraintestinal sites in patients with IBD and the pathogenesis of this condition can either depend on the expansion or translocation of an immune response from the gastrointestinal tract, or it can be a separate inflammatory event. Alternatively, it may share a common environment or genetic susceptibility with IBD.<br><br>For individuals with IBD, EIMs can affect multiple systems such as the skin, the musculoskeletal system, the hepatic system, and the eyes. The prevalence of EIMs in IBD patients ranges from 38% to 41%, with a higher susceptibility observed in individuals with CD than those with UC. According to the Swiss Inflammatory Bowel Disease Cohort Study (SIBDCS), an IBD cohort study focusing on EIMs, it is accepted that approximately 25% of individuals with IBD could experience multiple EIMs, possibly up to 5 types. EIMs greatly affect the life quality of individuals with IBD, adding to the disease's overall burden and significantly influencing the mortality rates among patients. Therefore, a comprehensive understanding of the pathogenic factors of EIMs is crucial with the aim of effectively tailoring therapeutic strategies that address all facets of IBD with EIMs.<br><br>Recently, the pathophysiology of EIMs in IBD has gained great interest. Some studies have proposed that the pathogenesis of the EIMs may originate in either translocation or extension of the immune response in the intestine, a relatively independent inflammatory process, or a shared environment or genetic susceptibility with IBD. Certain EIMs, like oral aphthous ulcers, erythema nodosum (EN) and episcleritis, may be directly associated with bowel diseases activity, while associations with primary sclerosing cholangitis (PSC) and pyoderma gangrenosum remain uncertain. Furthermore, according to recent genome-wide association studies (GWAS), it is recognized that there is a significant genetic overlap

				between EIMs and IBD. For instance, there is a significant genetic association between EN and susceptibility variants of IBD, such as ITGAL, PTGER4, CD207, SOCS5, and ITGB3, . Additionally, IBD risk variants, such as BCL2L11, UBASH3A, FOXO1, IRF8, JAK2, STAT3, SOCS1, and TYK2, have been identified in patients with PSC. Moreover, environment, immune system status, microbiota, and microbial products are also believed to contribute to the pathogenic process.<br><br>Despite extensive research conducted in this field, establishing causal inferences based on these studies is difficult to ascribe to the potential influence of reverse causality, confounding, or measurement error. Furthermore, the implementation of randomized clinical trials (RCTs) presents numerous challenges, including high costs, the need for substantial human resources, time-intensive procedures, and ethical limitations, which hinder their execution. As a substitute, Mendelian Randomization (MR) provides a more feasible approach to testing causality between exposure and outcome, mimicking the design of RCTs.
3	**Objectives**	State specific objectives clearly, including pre-specified causal hypotheses (if any). State that MR is a method that, under specific assumptions, intends to estimate causal effects	8	Given the fixed and randomly assigned nature of genetic variants at conception, MR analyses offer a feasible approach for testing causality between exposure and outcome, resembling RCTs by utilizing genetic variants as instrumental variables (IVs) to evaluate causal relationships while minimizing confounding and reverse causation. Additionally, this study's exposure variables were not affected by traditional factors, for instance, environmental exposures and behaviors, while satisfying the criterion of temporal precedence, indicating causality precedes outcome occurrence. Conducting a two-sample MR analysis involves obtaining IVs associated with the exposure and outcome from separate population datasets, thereby enhancing statistical power. Hence, to evaluate the likely causal impacts of genetically anticipated IBD, UC and CD on EIMs risk, we implemented a two-sample MR analysis using recently published GWAS data.
	**METHODS**		9-16	
4	**Study design and data sources**	Present key elements of the study design early in the article. Consider including a table listing sources of data for all phases of the study. For each data source contributing to the analysis, describe the following:	9-14	● Figure 1 presents a flowchart illustrating the analytical methods employed and outlines the process of conducting the MR analysis. (Page 10)<br>● Table 1 demonstrates the comprehensive information regarding the GWAS data for both the exposure and outcome. (Page 14)
	a)	Setting: Describe the study design and the underlying population, if possible. Describe the setting, locations, and relevant dates, including periods of recruitment, exposure, follow-up, and data collection, when available.	9-10	We executed a two-sample MR analysis using GWAS summary data to assess the causal impacts of genetically anticipated IBD on EIMs. In addition, to further investigate differences between subtypes, we conducted separate analyses for UC and CD. This approach allowed us to explore potential heterogeneity in the causal relationships between each IBD subtype and specific EIMs. EIMs primarily affect the skin (e.g., EN and pyoderma gangrenosum), eyes (e.g., scleritis, episcleritis, and uveitis), the

			hepatobiliary system (e.g., PSC), and the musculoskeletal system (e.g., spondyloarthritis and enthesitis). Integrating GWAS results and literature reports, we selected six kinds of EIMs as our outcomes, including EN, episcleritis, scleritis, uveitis, PSC, and spondyloarthritis. To mitigate the risk of sample overlap between exposure and outcome, and to minimize potential bias arising from racial differences, both exposure and outcome GWAS data were obtained from distinct cohorts within the European population. Figure 1 presents a flowchart illustrating the analytical methods employed and outlines the process of conducting the MR analysis.
b)	Participants: Give the eligibility criteria, and the sources and methods of selection of participants. Report the sample size, and whether any power or sample size calculations were carried out prior to the main analysis	10-13	The SNPs linked to IBD, encompassing both UC and CD, were obtained from the comprehensive summary data of the largest GWAS carried out so far, which were sourced from the International IBD Genetics Consortium (IIBDGC), which encompassed 12,882 cases of IBD and 21,770 individuals without the condition, 6,968 cases of UC and 20,464 controls, as well as 5,956 cases of CD and 14,927 controls. To increase the credibility of this research, we included summary statistics from the extended cohort for IBD (38,565 cases and 37,747 controls), along with 10,920 UC cases and 15,977 controls, and 14,763 CD cases and 15,977 controls for replication. Following the integration of GWAS results and literature reports, SNPs associated with EIMs were identified.<br><br>GWAS data for four common types of EIMs — erythema nodosum (433 cases), episcleritis (660 cases), scleritis (121 cases), and spondyloarthritis (3,037 cases) — were retrieved from the Finngen database (https://www.finngen.fi/en/access_results). SNPs related to PSC were obtained from the International PSC Study Group (IPSCSG), encompassing a cohort of 4,796 cases and 19,955 controls. Additionally, the uveitis-associated SNPs were accessed from the GWAS study with the data from UK Biobank and FinnGen. As our study solely relies on publicly accessible GWAS summary statistics and does not involve the identification of individual-level data, ethical approval was not pursued. Table 1 demonstrates the comprehensive information regarding the GWAS data for both the exposure and outcome.
c)	Describe measurement, quality control and selection of genetic variants	14-15	SNPs serving as effective instrumental variables should satisfy 3 critical assumptions: (1) Strong association need to exist between SNPs and the exposure; (2) SNPs should be independent of confounders that are relevant to the outcome; (3) These SNPs should have no direct relation with the outcome and can only affect the outcome through exposure factors.20 To meet these assumptions, we applied a sequence of quality control measures. Firstly, to extract the SNPs closely related to exposures, we applied a strict threshold of P < 5 × 10-8 to determine the significance of these SNPs.21 Secondly, to avoid linkage disequilibrium (LD) between genetic variants, we employed linkage disequilibrium clumping to confirm the independence of SNPs used in this analysis. Specifically, we set criteria of R^2 <0.001 and a minimum LD distance of 10,000 kb to determine the independence of SNPs. Thirdly, the application of high-intensity IVs can significantly enhance the accuracy and efficiency of the MR model in

					estimating causal effects. To reduce the potential bias caused by weak IVs, we assessed the strength of IVs using the F-statistic. If the F-statistic is less than 10, the estimates of causal effects may be severely biased, and IVs should be excluded from the analysis.21 Additionally, to mitigate potential interference from confounding factors and horizontal pleiotropy, we utilized the PhenoScanner database (http://www.phenoscanner.medschl.cam.ac.uk/phenoscanner) to investigate the associations between the selected IVs and other phenotypes that may pose a risk of influencing the outcome.
		d)	For each exposure, outcome, and other relevant variables, describe methods of assessment and diagnostic criteria for diseases	11-13	The diagnosis of IBD, UC and CD is multifaceted, involving clinical, biochemical, endoscopic, radiological, and histological investigations. Histological evaluation further aids in the diagnosis, with distinct features for UC and CD. Laboratory investigations play a crucial role in the diagnostic workup, with a full blood count, inflammatory markers (e.g. C-reactive protein), electrolytes, liver enzymes, and stool samples for microbiological analysis being standard assessments. For UC, the presence of mucosal inflammation and architectural distortion is typical, while CD may show transmural inflammation, granulomas, and focal inflammation.<br><br>Erythema nodosum typically manifests as tender, raised subcutaneous nodules on the lower limbs, with a predilection for females over males. These nodules, which are red or purple in color, range from 1 to 5 centimeters in diameter. Episcleritis is characterized by symptoms such as ocular burning, irritation, pain, and redness. It is crucial to clinically distinguish episcleritis from scleritis, as the latter represents a more serious condition. Scleritis is marked by severe ocular pain and tenderness, which can be indicative of a more profound inflammatory process affecting the sclera. Uveitis can present with a range of symptoms including pain, redness, photophobia, and changes in vision. The nature of the inflammation (e.g., anterior, intermediate, posterior, or panuveitis) can guide the diagnostic process. SpA often presents with inflammatory back pain (IBP), which can be assessed using criteria such as insidious onset, age at onset under 40 years, duration of back pain over 3 months, morning stiffness, and improvement with exercise.<br><br>PSC is characterized by inflammation and fibrosis affecting both the intra- and extrahepatic biliary tracts. It often presents with symptoms such as right upper quadrant (RUQ) pain, fever, fatigue, jaundice, pruritus, and weight loss. Hepatic function tests exhibit a cholestatic pattern, indicative of impaired bile flow. Magnetic resonance cholangiography (MRC) is a diagnostic imaging technique that reveals the signature 'beads-on-a-string' appearance in PSC, showcasing multiple segmental bile duct strictures and dilatations, which are pathognomonic for the disease.
		e)	Provide details of ethics committee approval and participant informed consent, if relevant	Not applicable.	Not applicable.
5	**Assumptions**		Explicitly state the three core IV assumptions for the main	14-15	SNPs serving as effective instrumental variables should satisfy 3 critical

			analysis (relevance, independence and exclusion restriction) as well assumptions for any additional or sensitivity analysis		assumptions: (1) Strong association need to exist between SNPs and the exposure; (2) SNPs should be independent of confounders that are relevant to the outcome; (3) These SNPs should have no direct relation with the outcome and can only affect the outcome through exposure factors. To meet these assumptions, we applied a sequence of quality control measures. Firstly, to extract the SNPs closely related to exposures, we applied a strict threshold of P < 5 × 10-8 to determine the significance of these SNPs.21 Secondly, to avoid linkage disequilibrium (LD) between genetic variants, we employed linkage disequilibrium clumping to confirm the independence of SNPs used in this analysis. Specifically, we set criteria of R^2 <0.001 and a minimum LD distance of 10,000 kb to determine the independence of SNPs. Thirdly, the application of high-intensity IVs can significantly enhance the accuracy and efficiency of the MR model in estimating causal effects. To reduce the potential bias caused by weak IVs, we assessed the strength of IVs using the F-statistic. If the F-statistic is less than 10, the estimates of causal effects may be severely biased, and IVs should be excluded from the analysis. Additionally, to mitigate potential interference from confounding factors and horizontal pleiotropy, we utilized the PhenoScanner database (http://www.phenoscanner.medschl.cam.ac.uk/phenoscanner) to investigate the associations between the selected IVs and other phenotypes that may pose a risk of influencing the outcome.
6	**Statistical methods: main analysis**		Describe statistical methods and statistics used	14-16	
		a)	Describe how quantitative variables were handled in the analyses (i.e., scale, units, model)	Not applicable.	Not applicable.
		b)	Describe how genetic variants were handled in the analyses and, if applicable, how their weights were selected	14 - 16	Firstly, to extract the SNPs closely related to exposures, we applied a strict threshold of P < 5 × 10-8 to determine the significance of these SNPs. Secondly, to avoid linkage disequilibrium (LD) between genetic variants, we employed linkage disequilibrium clumping to confirm the independence of SNPs used in this analysis. Specifically, we set criteria of R^2 <0.001 and a minimum LD distance of 10,000 kb to determine the independence of SNPs. Thirdly, the application of high-intensity IVs can significantly enhance the accuracy and efficiency of the MR model in estimating causal effects. To reduce the potential bias caused by weak IVs, we assessed the strength of IVs using the F-statistic. If the F-statistic is less than 10, the estimates of causal effects may be severely biased, and IVs should be excluded from the analysis. Additionally, to mitigate potential interference from confounding factors and horizontal pleiotropy, we utilized the PhenoScanner database (http://www.phenoscanner.medschl.cam.ac.uk/phenoscanner) to investigate the associations between the selected IVs and other phenotypes that may pose a risk of influencing the outcome.<br><br>To evaluate the likely causal relationship between IBD, including UC and

			CD with six EIMs, we employed random-effects inverse variance weighting (IVW), MR-Egger, and weight median estimator (WME) for MR analysis. The results conducted by utilizing the IVW method stand as the main estimates for MR analysis due to its ability to provide reliable causal estimates, particularly when there is no presence of directional pleiotropy.22 The results of the WME and MR-Egger tests can be complementary with the IVW method for more comprehensive assessments. Additionally, a serious of sensitivity analyses, including heterogeneity and pleiotropy, were performed to improve the reliability and stability of the results. We utilized Cochran's Q test to quantify the heterogeneity of IVs, signifying its presence when the p-value (P) was less than 0.05. Upon detecting heterogeneity, MR-PRESSO (NbDistribution = 10000) was utilized to identify specific SNPs contributing to the observed heterogeneity.23 Subsequently, after removing outliers, reapplication of the MR analysis was necessary. The MR-Egger intercept test was employed to evaluate the potential pleiotropy for the selected IVs. A leave-one-out sensitivity test was performed to examine the significant effect of individual SNPs on the causal effect. Additionally, we employed visualization, including scatter plots and funnel plots, to combine with statistical tests to discern pleiotropic instrumental variables.<br><br>Further, the sample size of exposures in the replication practice was significantly larger compared to the initial practice, and this difference may impact the accuracy and efficiency of the MR Model. To mitigate bias from sample differences and enhance the quality and reliability of research outcomes, we employed the fixed-effects meta-analysis to consolidate MR results from both the IVW approach of the initial practice and the replication practice. To assess reverse causality, we conducted additional MR analyses, considering EIMs as the exposure and IBD as the outcome, to evaluate the potential causal effects of EIMs on IBD. It helped us to identify and exclude possible reverse causal relationships, thereby enhancing the credibility of our findings.
c)	Describe the MR estimator (e.g. two-stage least squares, Wald ratio) and related statistics. Detail the included covariates and, in case of two-sample MR, whether the same covariate set was used for adjustment in the two samples	15	To evaluate the likely causal relationship between IBD, including UC and CD with six EIMs, we employed random-effects inverse variance weighting (IVW), MR-Egger, and weight median estimator (WME) for MR analysis. The results conducted by utilizing the IVW method stand as the main estimates for MR analysis due to its ability to provide reliable causal estimates, particularly when there is no presence of directional pleiotropy. The results of the WME and MR-Egger tests can be complementary with the IVW method for more comprehensive assessments.
d)	Explain how missing data were addressed	13	According to relevant GWAS databases and reports, researchers typically implemented a series of rigorous quality control steps to ensure data reliability and validity. First, the completeness of sample data was assessed by calculating the amount and distribution of missing values. Samples with a missing rate exceeding 5% were flagged and excluded to minimize data noise and analytical bias. Additionally, genetic correlations between samples were computed to identify and remove highly correlated

					individuals. In terms of genotype quality control, researchers commonly used multiple methods to evaluate data reliability. For instance, SNPs with high missing rates were excluded, and Hardy-Weinberg Equilibrium (HWE) tests were conducted to detect deviations from genetic equilibrium within populations, ensuring the reasonableness of genotype distributions. These measured effectively mitigate potential impacts from technical errors and biological anomalies on data analysis.
		e)	If applicable, indicate how multiple testing was addressed	Not applicable.	Not applicable.
7	**Assessment of assumptions**		Describe any methods or prior knowledge used to assess the assumptions or justify their validity	14-15	SNPs serving as effective instrumental variables should satisfy 3 critical assumptions: (1) Strong association need to exist between SNPs and the exposure; (2) SNPs should be independent of confounders that are relevant to the outcome; (3) These SNPs should have no direct relation with the outcome and can only affect the outcome through exposure factors.20 To meet these assumptions, we applied a sequence of quality control measures. Firstly, to extract the SNPs closely related to exposures, we applied a strict threshold of $P < 5 \times 10\text{-}8$ to determine the significance of these SNPs.21 Secondly, to avoid linkage disequilibrium (LD) between genetic variants, we employed linkage disequilibrium clumping to confirm the independence of SNPs used in this analysis. Specifically, we set criteria of R^2 <0.001 and a minimum LD distance of 10,000 kb to determine the independence of SNPs. Thirdly, the application of high-intensity IVs can significantly enhance the accuracy and efficiency of the MR model in estimating causal effects. To reduce the potential bias caused by weak IVs, we assessed the strength of IVs using the F-statistic. If the F-statistic is less than 10, the estimates of causal effects may be severely biased, and IVs should be excluded from the analysis.21 Additionally, to mitigate potential interference from confounding factors and horizontal pleiotropy, we utilized the PhenoScanner database (http://www.phenoscanner.medschl.cam.ac.uk/phenoscanner) to investigate the associations between the selected IVs and other phenotypes that may pose a risk of influencing the outcome.
8	**Sensitivity analyses and additional analyses**		Describe any sensitivity analyses or additional analyses performed (e.g. comparison of effect estimates from different approaches, independent replication, bias analytic techniques, validation of instruments, simulations)	15-16	Additionally, a serious of sensitivity analyses, including heterogeneity and pleiotropy, were performed to improve the reliability and stability of the results. We utilized Cochran's Q test to quantify the heterogeneity of IVs, signifying its presence when the p-value (P) was less than 0.05. Upon detecting heterogeneity, MR-PRESSO (NbDistribution = 10000) was utilized to identify specific SNPs contributing to the observed heterogeneity. Subsequently, after removing outliers, reapplication of the MR analysis was necessary. The MR-Egger intercept test was employed to evaluate the potential pleiotropy for the selected IVs. A leave-one-out sensitivity test was performed to examine the significant effect of individual SNPs on the causal effect. Additionally, we employed visualization, including scatter plots and funnel plots, to combine with statistical tests to discern pleiotropic instrumental variables.

				Further, the sample size of exposures in the replication practice was significantly larger compared to the initial practice, and this difference may impact the accuracy and efficiency of the MR Model. To mitigate bias from sample differences and enhance the quality and reliability of research outcomes, we employed the fixed-effects meta-analysis to consolidate MR results from both the IVW approach of the initial practice and the replication practice. To assess reverse causality, we conducted additional MR analyses, considering EIMs as the exposure and IBD as the outcome, to evaluate the potential causal effects of EIMs on IBD. It helped us to identify and exclude possible reverse causal relationships, thereby enhancing the credibility of our findings.
9	**Software and pre-registration**		16	
		a) Name statistical software and package(s), including version and settings used	16	The MR analyses were conducted using the TwoSampleMR and MRPRESSO packages within R version 4.1.2 software. The meta-analyses were performed using the “meta” R package. The Forestploter R package was used to draw forest plots.
		b) State whether the study protocol and details were pre-registered (as well as when and where)	Not applicable.	Not applicable.
	**RESULTS**		17-24	
10	**Descriptive data**			
		a) Report the numbers of individuals at each stage of included studies and reasons for exclusion. Consider use of a flow diagram	Not applicable.	Due to the large volume of data processing involved, we are unable to present the specific results of each data processing stage in detail within the main text. However, all the data used for the study, as well as the processed data, are thoroughly listed and summarized in the supplementary materials. In the supplementary materials, you will find the number of samples at each stage and the reasons for exclusion. For clarity, we have also provided a flow diagram outlining the sample selection and exclusion process at each stage of the study. This will help readers clearly understand the flow of samples throughout the study. (Figure 1)
		b) Report summary statistics for phenotypic exposure(s), outcome(s), and other relevant variables (e.g. means, SDs, proportions)	17	Additionally, the summary statistics for the phenotypic exposures and outcomes are provided in detail in the **supplementary materials**.
		c) If the data sources include meta-analyses of previous studies, provide the assessments of heterogeneity across these studies	Not applicable.	Not applicable.
		d) For two-sample MR:<br>i. Provide justification of the similarity of the genetic variant-exposure associations between the exposure and	Not applicable.	Not applicable.

			outcome samples<br>ii. Provide information on the number of individuals who overlap between the exposure and outcome studies		
11	**Main results**				
		a)	Report the associations between genetic variant and exposure, and between genetic variant and outcome, preferably on an interpretable scale	17-24	**3.1 MR analysis for causal impacts of genetically anticipated IBD on EIMs**<br>Figure 2 shows a comparison between the results of the initial practice and repeated practice based on the IVW analysis. As can be seen in Figure 3, in the initial practice, it appears that genetically anticipated IBD contributes to a causal relationship with increased EN (OR, 1.18; 95%Cl, 1.03-1.35; p=0.017), uveitis (OR, 1.13; 95%Cl, 1.07-1.21; p =6.62*10-5), PSC (OR, 1.20; 95%Cl, 1.09-1.32; p = 2.5*10-4) and spondyloarthritis (OR, 1.17; 95%Cl, 1.11-1.24; p=4.25*10-8) susceptibility using IVW method. Furthermore, there is no evidence to indicate a causal relationship between IBD and episcleritis or scleritis. In the replication practice, it provides evidence supporting a causal relationship between IBD and an increased risk of EN, uveitis, PSC, and spondyloarthritis, which aligns with the findings of the initial practice. The results suggested that there is no causal relationship between IBD and episcleritis or scleritis. Additionally, the results of both scatter plots and symmetric funnel plots indicated that no pleiotropy was identified. In addition, the leave-one-out analysis further verified the robustness of our results. The supplementary figures 1-6 provide the funnel plots, scatter plots, and leave-one-out analysis for each outcome respectively.<br>No horizontal pleiotropy effect was detected, which indicated that the results were credible in this study. In addition, heterogeneity was detected in certain MR analyses. We performed the MR-PRESSO outliner test to confirm the specific SNPs led to the heterogeneous results and the information on these outliers is shown in **Table 2**. After removing these outliers and reapplying the MR analyses, the level of heterogeneity decreased.<br>**3.2 MR analysis for causal impacts of genetically anticipated UC on EIMs**<br>The results of the IVW analysis for UC and EIMs in the initial and replication practices are presented in Figure 3. We found evidence of a causal relationship between UC and increased susceptibility to PSC (OR, 1.26; 95% CI, 1.13-1.41; p = 0.00005) and spondyloarthritis (OR, 1.08; 95% CI, 1.01-1.17; p = 0.011) in the initial practice. Additionally, no causal relationship between UC and EN, uveitis, episcleritis or scleritis was identified. In the replication practice, some of these results were slightly different from those observed in the initial practice. The results from the replication provided evidence of a causal relationship between UC and an

			increased risk of entities, including EN, uveitis, PSC, and spondyloarthritis. This discrepancy between the initial practice and the replication practice may be attributed to variations in data collected from different sources. In addition, the absence of pleiotropic bias is suggested based on the analysis of scatter plots and symmetric funnel plots, while the robustness of our estimation results is further demonstrated through leave-one-out analysis. Funnel plots, scatter plots, and leave-one-out analysis plots for each outcome in the replication practice were presented **in supplementary figures 7-12.**<br><br>Furthermore, heterogeneity was observed in certain analyses, and the information on the outliers was presented in Table 3. Upon removing these specific SNPs and repeating the MR analysis, the heterogeneity became insignificant. (P>0.05). No evidence of horizontal pleiotropy was found in any MR analysis results, indicating the credibility of the study findings.<br><br>**3.3 MR analysis for causal impacts of genetically anticipated CD with EIMs**<br><br>The results of the IVW analysis focusing on CD and EIMs in the initial and replication practices are presented in Figure 4. In the initial practice, we observed the causal impacts of genetically predicted CD on the increased risk of EN (OR, 1.25; 95%Cl, 1.12-1.40; p<0.01), uveitis (OR, 1.08; 95%Cl, 1.03-1.13; p<0.01) and spondyloarthritis (OR, 1.10; 95%Cl, 1.05-1.15; p<0.01) after correcting for multiple testing, and no causal relationships between CD and episcleritis, scleritis or PSC were identified. In the replication practice, comparable research findings were observed. The results showed a causal link between CD and an increase in the risk of PSC, which was not consistent with initial practice. Both scatter and symmetric funnel plots confirmed that there was no pleiotropic bias present. Additionally, it is believed that the results are stable according to the leave-one-out analysis plots. The supplementary figures 13-18 provide the funnel plots, scatter plots, and leave-one-out analysis plots for each outcome respectively.<br><br>In addition, certain MR analyses showed heterogeneity and the information on the outliers was presented in **Table 4.** After removing these outliers, the MR analysis was repeated and the results showed that heterogeneity was no longer significant. The results of all analyses did not show pleiotropy, suggesting that the results of this analysis were reliable.
	b) Report MR estimates of the relationship between exposure and outcome, and the measures of uncertainty from the MR analysis, on an interpretable scale, such as odds ratio or relative risk per SD difference	17-24	**3.1 MR analysis for causal impacts of genetically anticipated IBD on EIMs**<br><br>As can be seen in Figure 3, in the initial practice, it appears that genetically anticipated IBD contributes to a causal relationship with increased EN (OR,

			1.18; 95%CI, 1.03-1.35; p=0.017), uveitis (OR, 1.13; 95%CI, 1.07-1.21; p =6.62*10-5), PSC (OR, 1.20; 95%CI, 1.09-1.32; p = 2.5*10-4) and spondyloarthritis (OR, 1.17; 95%CI, 1.11-1.24; p=4.25*10-8) susceptibility using IVW method. Furthermore, there is no evidence to indicate a causal relationship between IBD and episcleritis or scleritis. In the replication practice, it provides evidence supporting a causal relationship between IBD and an increased risk of EN, uveitis, PSC, and spondyloarthritis, which aligns with the findings of the initial practice. The results suggested that there is no causal relationship between IBD and episcleritis or scleritis.<br>**3.2 MR analysis for causal impacts of genetically anticipated UC on EIMs**<br>We found evidence of a causal relationship between UC and increased susceptibility to PSC (OR, 1.26; 95% CI, 1.13-1.41; p = 0.00005) and spondyloarthritis (OR, 1.08; 95% CI, 1.01-1.17; p = 0.011) in the initial practice. Additionally, no causal relationship between UC and EN, uveitis, episcleritis or scleritis was identified. In the replication practice, some of these results were slightly different from those observed in the initial practice. The results from the replication provided evidence of a causal relationship between UC and an increased risk of entities, including EN, uveitis, PSC, and spondyloarthritis.<br>**3.3 MR analysis for causal impacts of genetically anticipated CD with EIMs**<br>In the initial practice, we observed the causal impacts of genetically predicted CD on the increased risk of EN (OR, 1.25; 95%CI, 1.12-1.40; p<0.01), uveitis (OR, 1.08; 95%CI, 1.03-1.13; p<0.01) and spondyloarthritis (OR, 1.10; 95%CI, 1.05-1.15; p<0.01) after correcting for multiple testing, and no causal relationships between CD and episcleritis, scleritis or PSC were identified. In the replication practice, comparable research findings were observed. The results showed a causal link between CD and an increase in the risk of PSC, which was not consistent with initial practice.
c)	If relevant, consider translating estimates of relative risk into absolute risk for a meaningful time period	Not applicable.	Not applicable.
d)	Consider plots to visualize results (e.g. forest plot, scatterplot of associations between genetic variants and outcome versus between genetic variants and exposure)	17-24	● Figure 2 shows a comparison between the results of the initial practice and repeated practice based on the IVW analysis.<br>● The results of the IVW analysis for UC and EIMs in the initial and replication practices are presented in Figure 3.<br>● The results of the IVW analysis focusing on CD and EIMs in the initial and replication practices are presented in Figure 4.<br>● The meta-analysis results are presented in Figure 5.<br>● The results of the IVW analysis focusing on EIMs and IBD, comprising UC and CD, are presented in Figure 6.

12	**Assessment of assumptions**				
		a)	Report the assessment of the validity of the assumptions	16	After applying stringent selection criteria as previously described, exposure-associated SNPs were identified as IVs for further MR analyses. Comprehensive information regarding the exposure-associated SNPs (IBD, UC, and CD) can be found in the Supplementary table 3-8. These SNPs were chosen based on their robust association with the exposure, thereby satisfying the relevance assumption. The F-values of IVs used in the study were significantly greater than 10, which indicated a low possibility of bias due to weak IVs. This threshold helps to mitigate the potential for weak instrument bias and ensures that our IVs are sufficiently predictive of the exposure. The IVW method was used to obtain the primary results from the MR analysis. Supplementary Table 1 and Supplementary Table 2 contain the results obtained from other MR analysis methods used in the initial and replication practice, respectively. Additionally, the summary statistics for the phenotypic exposures and outcomes and proportions, are provided in detail in the supplementary materials.
		b)	Report any additional statistics (e.g., assessments of heterogeneity across genetic variants, such as I^2, Q statistic or E-value)	22-24	**3.4 Meta-analysis to combine MR results obtained from different data sources**<br><br>The meta-analysis results are presented in Figure 5. The combined results provided evidence of a causal relationship between genetically anticipated IBD and increased risks of entities, including EN (OR, 1.20; 95% CI, 1.09-1.32; p < 0.01), uveitis (OR, 1.15; 95% CI, 1.11-1.20; p < 0.01), PSC (OR, 1.21; 95% CI, 1.13-1.28; p < 0.01), and spondyloarthritis (OR, 1.19; 95% CI, 1.14-1.23; p < 0.0001).<br><br>In subgroup analyses, the results indicated an causal relationship between UC and increased risks of entities, including EN (OR, 1.12; 95%CI, 1.02-1.23; p=0.0228), uveitis (OR, 1.11; 95%CI, 1.06-1.17; p<0.0001), PSC (OR, 1.25; 95%CI, 1.17-1.35; p<0.0001), and spondyloarthritis (OR, 1.11; 95%CI, 1.06-1.17; p<0.0001). In addition, genetically predicted CD showed a causal relationship with increased risks of EN (OR, 1.22; 95% CI, 1.12-1.32; p < 0.0001), uveitis (OR, 1.09; 95% CI, 1.05-1.13; p < 0.0001), PSC (OR, 1.12; 95% CI, 1.04-1.14; p < 0.0001), and spondyloarthritis (OR, 1.11; 95% CI, 1.09-1.16; p < 0.0001).<br><br>The most surprising aspect of the results was that no causal effect of IBD on episcleritis or scleritis was found. In the subgroup analyses, no causal relationship could be established between UC and CD and the occurrence of episcleritis or scleritis.<br><br>**3.5 MR analysis for causal impacts of genetically anticipated EIMs with IBD, UC and CD**<br><br>The results of the IVW analysis focusing on EIMs and IBD, comprising UC and CD, are presented in Figure 6. The results of MR studies provided

					evidence of a causal relationship between genetically predicted PSC and an elevated risk of IBD (OR, 1.21; 95%CI, 1.15-1.29; p<0.01), UC (OR, 1.27; 95%CI, 1.17-1.37; p<0.01) and CD (OR, 1.10; 95%CI, 1.02-1.20; p<0.01). In addition, the results demonstrated a causal relationship between spondyloarthritis and an increased risk of both IBD (OR, 1.03; 95% CI, 1.01-1.06; p < 0.01) and UC (OR, 1.05; 95% CI, 1.02-1.08; p < 0.01).<br><br>The study employed several sensitivity analyses to assess the robustness of the findings. MR-Egger regression was used to detect horizontal pleiotropy, and no significant pleiotropy was observed in this study. Additionally, heterogeneity tests were performed. After removing these outliers, the MR analysis was repeated and the results showed that heterogeneity was no longer significant. The supplementary figures 19-24 provide the funnel plots, scatter plots, and leave-one-out analysis MR analysis for causal impacts of genetically anticipated EIMs with IBD, UC and CD.
13	**Sensitivity analyses and additional analyses**				
		a)	Report any sensitivity analyses to assess the robustness of the main results to violations of the assumptions	17-24	**3.1 MR analysis for causal impacts of genetically anticipated IBD on EIMs**<br><br>Additionally, the results of both scatter plots and symmetric funnel plots indicated that no pleiotropy was identified. In addition, the leave-one-out analysis further verified the robustness of our results. The supplementary figures 1-6 provide the funnel plots, scatter plots, and leave-one-out analysis for each outcome respectively. No horizontal pleiotropy effect was detected, which indicated that the results were credible in this study. In addition, heterogeneity was detected in certain MR analyses. We performed the MR-PRESSO outliner test to confirm the specific SNPs led to the heterogeneous results and the information on these outliers is shown in Table 2. After removing these outliers and reapplying the MR analyses, the level of heterogeneity decreased.<br><br>**3.2 MR analysis for causal impacts of genetically anticipated UC on EIMs**<br><br>In addition, the absence of pleiotropic bias is suggested based on the analysis of scatter plots and symmetric funnel plots, while the robustness of our estimation results is further demonstrated through leave-one-out analysis. Funnel plots, scatter plots, and leave-one-out analysis plots for each outcome in the replication practice were presented in supplementary figures 7-12. Furthermore, heterogeneity was observed in certain analyses, and the information on the outliers was presented in Table 3. Upon removing these specific SNPs and repeating the MR analysis, the

			heterogeneity became insignificant. (P>0.05). No evidence of horizontal pleiotropy was found in any MR analysis results, indicating the credibility of the study findings.<br><br>**3.3 MR analysis for causal impacts of genetically anticipated CD with EIMs**<br><br>The results showed a causal link between CD and an increase in the risk of PSC, which was not consistent with initial practice. Both scatter and symmetric funnel plots confirmed that there was no pleiotropic bias present. Additionally, it is believed that the results are stable according to the leave-one-out analysis plots. The supplementary figures 13-18 provide the funnel plots, scatter plots, and leave-one-out analysis plots for each outcome respectively. In addition, certain MR analyses showed heterogeneity and the information on the outliers was presented in Table 4. After removing these outliers, the MR analysis was repeated and the results showed that heterogeneity was no longer significant. The results of all analyses did not show pleiotropy, suggesting that the results of this analysis were reliable.
b)	Report results from other sensitivity analyses or additional analyses	17-24	**3.4 Meta-analysis to combine MR results obtained from different data sources**<br><br>The meta-analysis results are presented in Figure 5. The combined results provided evidence of a causal relationship between genetically anticipated IBD and increased risks of entities, including EN (OR, 1.20; 95% CI, 1.09-1.32; p < 0.01), uveitis (OR, 1.15; 95% CI, 1.11-1.20; p < 0.01), PSC (OR, 1.21; 95% CI, 1.13-1.28; p < 0.01), and spondyloarthritis (OR, 1.19; 95% CI, 1.14-1.23; p < 0.0001).<br><br>In subgroup analyses, the results indicated an causal relationship between UC and increased risks of entities, including EN (OR, 1.12; 95%CI, 1.02-1.23; p=0.0228), uveitis (OR, 1.11; 95%CI, 1.06-1.17; p<0.0001), PSC (OR, 1.25; 95%CI, 1.17-1.35; p<0.0001), and spondyloarthritis (OR, 1.11; 95%CI, 1.06-1.17; p<0.0001). In addition, genetically predicted CD showed a causal relationship with increased risks of EN (OR, 1.22; 95% CI, 1.12-1.32; p < 0.0001), uveitis (OR, 1.09; 95% CI, 1.05-1.13; p < 0.0001), PSC (OR, 1.12; 95% CI, 1.04-1.14; p < 0.0001), and spondyloarthritis (OR, 1.11; 95% CI, 1.09-1.16; p < 0.0001).<br><br>The most surprising aspect of the results was that no causal effect of IBD on episcleritis or scleritis was found. In the subgroup analyses, no causal relationship could be established between UC and CD and the occurrence of episcleritis or scleritis.<br><br>**3.5 MR analysis for causal impacts of genetically anticipated EIMs with IBD, UC and CD**<br><br>The results of the IVW analysis focusing on EIMs and IBD, comprising UC and CD, are presented in Figure 6. The results of MR studies provided

			evidence of a causal relationship between genetically predicted PSC and an elevated risk of IBD (OR, 1.21; 95%CI, 1.15-1.29; p<0.01), UC (OR, 1.27; 95%CI, 1.17-1.37; p<0.01) and CD (OR, 1.10; 95%CI, 1.02-1.20; p<0.01). In addition, the results demonstrated a causal relationship between spondyloarthritis and an increased risk of both IBD (OR, 1.03; 95% CI, 1.01-1.06; p < 0.01) and UC (OR, 1.05; 95% CI, 1.02-1.08; p < 0.01).<br><br>The study employed several sensitivity analyses to assess the robustness of the findings. MR-Egger regression was used to detect horizontal pleiotropy, and no significant pleiotropy was observed in this study. Additionally, heterogeneity tests were performed. After removing these outliers, the MR analysis was repeated and the results showed that heterogeneity was no longer significant. The supplementary figures 19-24 provide the funnel plots, scatter plots, and leave-one-out analysis MR analysis for causal impacts of genetically anticipated EIMs with IBD, UC and CD.
c)	Report any assessment of direction of causal relationship (e.g., bidirectional MR)	23-24	**3.5 MR analysis for causal impacts of genetically anticipated EIMs with IBD, UC and CD**<br><br>The results of the IVW analysis focusing on EIMs and IBD, comprising UC and CD, are presented in Figure 6. The results of MR studies provided evidence of a causal relationship between genetically predicted PSC and an elevated risk of IBD (OR, 1.21; 95%CI, 1.15-1.29; p<0.01), UC (OR, 1.27; 95%CI, 1.17-1.37; p<0.01) and CD (OR, 1.10; 95%CI, 1.02-1.20; p<0.01). In addition, the results demonstrated a causal relationship between spondyloarthritis and an increased risk of both IBD (OR, 1.03; 95% CI, 1.01-1.06; p < 0.01) and UC (OR, 1.05; 95% CI, 1.02-1.08; p < 0.01).<br><br>The study employed several sensitivity analyses to assess the robustness of the findings. MR-Egger regression was used to detect horizontal pleiotropy, and no significant pleiotropy was observed in this study. Additionally, heterogeneity tests were performed. After removing these outliers, the MR analysis was repeated and the results showed that heterogeneity was no longer significant. The supplementary figures 19-24 provide the funnel plots, scatter plots, and leave-one-out analysis MR analysis for causal impacts of genetically anticipated EIMs with IBD, UC and CD.
d)	When relevant, report and compare with estimates from non-MR analyses	Not applicable.	Not applicable.
e)	Consider additional plots to visualize results (e.g., leave-one-out analyses)	17-24	• The leave-one-out analysis further verified the robustness of our results. The **supplementary figures 1-6** provide the funnel plots, scatter plots, and leave-one-out analysis for each outcome respectively.<br>• We performed the MR-PRESSO outliner test to confirm the specific

				SNPs led to the heterogeneous results and the information on these outliers is shown in **Table 2**.<br>● Funnel plots, scatter plots, and leave-one-out analysis plots for each outcome in the replication practice were presented in **supplementary figures 7-12**.<br>● Furthermore, heterogeneity was observed in certain analyses, and the information on the outliers was presented in **Table 3**.<br>● Additionally, it is believed that the results are stable according to the leave-one-out analysis plots. The **supplementary figures 13-18** provide the funnel plots, scatter plots, and leave-one-out analysis plots for each outcome respectively.<br>● In addition, certain MR analyses showed heterogeneity and the information on the outliers was presented in **Table 4**.
	**DISCUSSION**		25-32	
14	**Key results**	Summarize key results with reference to study objectives	25	This study provides compelling evidence of a causal relationship between genetically predicted IBD, encompassing UC and CD, and an increased risk of developing specific EIMs, including EN, uveitis, PSC, and spondyloarthritis. Additionally, no significant causal relationship was observed between IBD, UC, and CD, and the incidence of either episcleritis or scleritis (Figure 7). Moreover, reverse causality analyses revealed that certain EIMs, particularly PSC and spondyloarthritis, have a causal relationship with an elevated risk of IBD, UC, and CD. These findings highlight a bidirectional causal relationship between IBD and certain EIMs, suggesting that monitoring these conditions may play a crucial role in early detection and management strategies for IBD patients. Further exploration of these causal pathways could contribute to better clinical outcomes by providing a deeper understanding of the interconnection between IBD and its extraintestinal effects.
15	**Limitations**	Discuss limitations of the study, taking into account the validity of the IV assumptions, other sources of potential bias, and imprecision. Discuss both direction and magnitude of any potential bias and any efforts to address them	30-32	However, several limitations of this MR investigation should be taken into account. First, the SNPs statistics about the IBD, UC and CD we used were from a population of Europeans. The majority of SNPs of EIMs were derived from FinnGen databases. Although numerous published MR studies typically treat Finnish data as part of a broader European cohort, the genetic distinctions of the Finnish population do not fully address when interpreting results. Given the unique genetic profile of the Finnish population — marked by historical bottlenecks and relative genetic isolation — the inclusion of Finnish data among "European" samples may introduce population-specific biases that could impact generalizability. This is particularly relevant as the genetic diversity within Europe is substantial, and specific regional groups, like the Finnish, may differ genetically from other European populations. With the conduction of future GWAS studies on a larger scale, researchers will gain access to a greater multitude of IVs that fulfill the significance threshold required for conducting MR analysis

			with SNPs. This will enhance the reliability of the results obtained. Secondly, this study was conducted based on GWAS data from European populations. Due to differences in genetic structures between different ancestry, results based on specific populations may not be directly applicable to other populations. To confirm the generalizability of these genetic variants, future studies should be conducted in different racial groups. Thirdly, although multiple sensitivity analyses were performed, further research is needed to investigate whether each SNP locus serving as IVs satisfies three critical assumptions of MR analysis. Additionally, although we employed the MR-PRESSO outlier test and removed outliers upon detecting heterogeneity, statistical heterogeneity persists among certain instrumentally determined IV estimates, necessitating further discussion. Lastly, MR analyses are often limited to cross-sectional data, which restricts our ability to capture dynamic, time-dependent relationships between exposures and outcomes. Future longitudinal studies may provide insights into the temporal aspects of these associations, which would strengthen the causal interpretations derived from MR.
16 **Interpretation**			
	a) Meaning: Give a cautious overall interpretation of results in the context of their limitations and in comparison with other studies	25	This study provides compelling evidence of a causal relationship between genetically predicted IBD, encompassing UC and CD, and an increased risk of developing specific EIMs, including EN, uveitis, PSC, and spondyloarthritis. Additionally, no significant causal relationship was observed between IBD, UC, and CD, and the incidence of either episcleritis or scleritis (Figure 7). Moreover, reverse causality analyses revealed that certain EIMs, particularly PSC and spondyloarthritis, have a causal relationship with an elevated risk of IBD, UC, and CD. These findings highlight a bidirectional causal relationship between IBD and certain EIMs, suggesting that monitoring these conditions may play a crucial role in early detection and management strategies for IBD patients. Further exploration of these causal pathways could contribute to better clinical outcomes by providing a deeper understanding of the interconnection between IBD and its extraintestinal effects.
	b) Mechanism: Discuss underlying biological mechanisms that could drive a potential causal relationship between the investigated exposure and the outcome, and whether the gene-environment equivalence assumption is reasonable. Use causal language carefully, clarifying that IV estimates may provide causal effects only under certain assumptions	26-30	Numerous recent studies have indicated that individuals with IBD carrying specific HLA alleles are at an elevated risk of developing EIMs. For CD patients, the presence of HLA-DR1, HLA-A2, and HLA-DQw5 alleles is linked to EIMs, while in UC, this heightened risk is related to HLA-B27, HLA-B58, and HLA-DR103. Among these, HLA-B27 is recognized as a well-established risk factor for the progression of EIMs. Its correlation has been observed in the context of joint, skin, and ophthalmologic manifestations. Recently, some GWAS studies have found that some EIMs are associated with risk loci for IBD, highlighting the genetic overlap between these conditions. However, this overlap was not observed between IBD and PSC, illustrating immunogenetic distinct features of PSC compared to IBD.<br><br>Musculoskeletal EIMs confirmed by the skeletal radiological criteria are one

of the commonly observed manifestations, occurring in approximately 46% of IBD patients, and spondyloarthritis can occur in up to 25%.1 While the pathogenesis of spondyloarthritis has been extensively studied, the exact etiology and the mechanism remain under investigation. Although it has been suggested that the correlation between HLA and IBD-associated SpA is comparatively less obvious than other kinds of SpA, it is still evident that genetics could play a significant role in this condition, particularly the HLA-B27 gene. Several studies have shown that the presence of certain HLA alleles, such as HLA-B27 and HLA-DR1, contributes to the increased risk of developing musculoskeletal manifestations in IBD, and the interaction between bacteria and HLA-B27 alleles is considered significant in the pathogenesis of spondyloarthritis. In addition, the "gut-synovial axis" hypothesis suggests that activated Th1 and Th17 cells in the intestine of IBD patients can migrate to synovial tissue, causing joint inflammation.

PSC, the most common hepato-biliary EIMs in IBD, may affect 60-80% of IBD patients The prevalence of PSC can reach up to 5% in UC patients and lower in CD patients. Colonic autoantibodies from PSC patients show cross-reactivity with biliary epithelium, highlighting the gut-liver axis's role in PSC pathogenesis. The gut microbiota, essential for nutrient absorption and immune regulation, is implicated in PSC development via the gut-liver axis.36 Individuals with IBD exhibit reduced diversity in their gut microbiota, which increases relative abundances of potential enteric pathogenic bacteria, which will promote the migration of intestinal lymphocytes and facilitate bacteria and their products to move into the portal venous circulation through compromised mucosa. Another hypothesis suggests that anti-neutrophil cytoplasmic antibodies (P-ANCAs) may cross-react with colonic antigens in susceptible individuals, leading to abnormal immunoreactions and biliary inflammation. Furthermore, the GPBAR1 gene, responsible for encoding a G-protein involved in the absorption of bile salts, demonstrates elevated expression levels in both the ileum and colon, demonstrating a significant association with PSC.

Studies have reported the incidence of EN ranging from 5% to 15% in individuals with CD, and from 2-10% in individuals with UC. Some known IBD susceptibility genes, such as ITGAL, CD207, and ITGB3, are associated with EN. Previous studies have indicated an overexpression of genes encoding Th1 cytokines in the skin lesions of EN patients. Furthermore, the content of Th1 cytokines, including IFN-γ and IL-12, is significantly increased in the skin lesions as well as in the serum. These findings suggest that the Th1 cellular immune response may contribute to the pathogenesis of skin lesions of patients with EN. Besides, adhesion molecules and inflammatory mediators, like E-selectin, P-selectin, and platelet endothelial cell adhesion molecule-1, may account for the pathogenesis of EN. Additionally, deposition of immunocomplex has been observed around veins in the subcutaneous adipose connective tissue, suggesting the role of immune-complex reactions in the pathophysiology of EN. Anti-tumor necrosis factor (anti-TNF) medications have shown significant effectiveness in treating cutaneous EIMs, suggesting a potential

		shared pathogenic link involving TNF between EIM and IBD. Additionally, a previous study has discovered that the TNF-NF κ B pathway was upregulated in samples from patients with EN and pyoderma gangrenosum.<br><br>Between 4% and 12% of patients with IBD experience ocular manifestations, with these ocular EIMs being more prevalent in CD compared with UC. The primary ocular manifestation observed in the majority of IBD patients is episcleritis. Moreover, some exceptionally severe types, such as scleritis, are relatively uncommon. Research indicated that there exists a robust association between ocular inflammation and HLA alleles, including HLA-B27, B58, and HLA-DRB1. The unanticipated outcomes reported by this study indicate no evidence supporting a causal connection between IBD, including UC and CD, and the occurrence of episcleritis or scleritis. However, a strong causal relationship was observed with uveitis. This result has further strengthened our confidence that pathogenic mechanisms of episcleritis, scleritis and uveitis are different, although they are common ocular EIMs.<br><br>The immune system is believed to play a crucial role in the pathogenesis of scleritis and episcleritis. It is characterized by the infiltration of immune cells such as B cells and macrophages, which suggests a role for cell-mediated immunity in the disease process. In addition, in tissues of certain individuals with scleritis, the presence of immune complexes, such as those formed by protease 3 (PR3) antibodies, can trigger complement responses and lead to vasculitis. Moreover, matrix metalloproteinases (MMPs) contributes to scleral destruction, and their production can be induced by TNF-alpha, which is found in the tissues of some scleritis patients. A previous study demonstrated that certain genes, such as cytotoxic T lymphocyte-associated antigen-4 (CTLA4) and protein tyrosine phosphatase non-receptor type 22 (PTPN22), have the ability to regulate T lymphocyte activity. It is accepted that polymorphisms in these genes could contribute to the development of autoimmune responses. For uveitis, the pathogenesis is associated with the activation of Th1 and Th17 cells, which secrete inflammatory cytokines such as TNF-alpha, IL-17, IL-23, and IL-6, driving the inflammatory response. The specific bacterial species can influence the development of distinct T cell populations, including Th17 cells and IL-17, which contribute to inflammation. Certain research has indicated that the composition of the microbiome in the gut differs in the absence of disease susceptibility alleles associated with HLA. Moreover, research has confirmed that labeled leukocytes are capable of migrating from the intestine to the eye, substantiating a direct correlation between the gut and the eye. In summary, while both scleritis and uveitis involve immune-mediated mechanisms, scleritis is characterized by cells, antibody and complement-mediated processes, and matrix metalloproteinase activity. And uveitis is driven by Th1 and Th17 cell activation, gut microbiota interactions, and the gut-eye axis.
c) Clinical relevance: Discuss whether the results have clinical or public policy relevance, and to what extent they inform	30	When diagnosing and managing patients with EIMs, it is of great significance for the medical staff to pay attention to the causal relationship

		effect sizes of possible interventions		between IBD and EIMs. For certain EIMs which are the consequence of IBD, such as EN, PSC, uveitis, and spondyloarthritis, the proper management of IBD can potentially alleviate the burden of EIMs, positively impact the management and treatment of EIMs, and improve the life quality of EIMs patients.
17	**Generalizability**	Discuss the generalizability of the study results (a) to other populations, (b) across other exposure periods/timings, and (c) across other levels of exposure	31	The SNPs statistics about the IBD, UC and CD we used were from a population of Europeans. The majority of SNPs of EIMs were derived from FinnGen databases. Although numerous published MR studies typically treat Finnish data as part of a broader European cohort, the genetic distinctions of the Finnish population do not fully address when interpreting results. Given the unique genetic profile of the Finnish population—marked by historical bottlenecks and relative genetic isolation—the inclusion of Finnish data among "European" samples may introduce population-specific biases that could impact generalizability. This is particularly relevant as the genetic diversity within Europe is substantial, and specific regional groups, like the Finnish, may differ genetically from other European populations. With the conduction of future GWAS studies on a larger scale, researchers will gain access to a greater multitude of IVs that fulfill the significance threshold required for conducting MR analysis with SNPs. This will enhance the reliability of the results obtained. Secondly, this study was conducted based on GWAS data from European populations. Due to differences in genetic structures between different ancestry, results based on specific populations may not be directly applicable to other populations. To confirm the generalizability of these genetic variants, future studies should be conducted in different racial groups.
	**OTHER INFORMATION**		32-34	
18	**Funding**	Describe sources of funding and the role of funders in the present study and, if applicable, sources of funding for the databases and original study or studies on which the present study is based	33	This work received funding from Tianjin Health Research Project Nos. TJSJMYXYC-D2-027.
19	**Data and data sharing**	Provide the data used to perform all analyses or report where and how the data can be accessed, and reference these sources in the article. Provide the statistical code needed to reproduce the results in the article, or report whether the code is publicly accessible and if so, where	33	The study’s datasets are publicly available. The research paper includes the original contributions, which can be found in both the article and supplementary materials. For any further inquiries please contact the corresponding authors.
20	**Conflicts of Interest**	All authors should declare all potential conflicts of interest	33	The authors assert that they do not have any known competing financial interests or personal relationships that could be perceived as influencing the work presented in this paper.

This checklist is copyrighted by the Equator Network under the Creative Commons Attribution 3.0 Unported (CC BY 3.0) license.

1. Skrivankova VW, Richmond RC, Woolf BAR, Yarmolinsky J, Davies NM, Swanson SA, et al. Strengthening the Reporting of Observational Studies in Epidemiology using Mendelian Randomization (STROBE-MR) Statement. JAMA. 2021;under review.

2. Skrivankova VW, Richmond RC, Woolf BAR, Davies NM, Swanson SA, VanderWeele TJ, et al. Strengthening the Reporting of Observational Studies in Epidemiology using Mendelian Randomisation (STROBE-MR): Explanation and Elaboration. BMJ. 2021;375:n2233.
