# Supplementary material for: The causal impact of genetically predicted inflammatory bowel disease on extraintestinal manifestations: a mendelian randomization study
Source: BMC Gastroenterol. 2025 Mar 4;25:135. doi: 10.1186/s12876-024-03566-4 (PMC11881308; doi:10.1186/s12876-024-03566-4)

**Supplement Figure 1**. Scatter plots for genetically predicted IBD on EN (A), episcleritis (B), scleritis (C), uveitis (D), PSC (E),

and spondyloarthritis (F) in the initial practice.


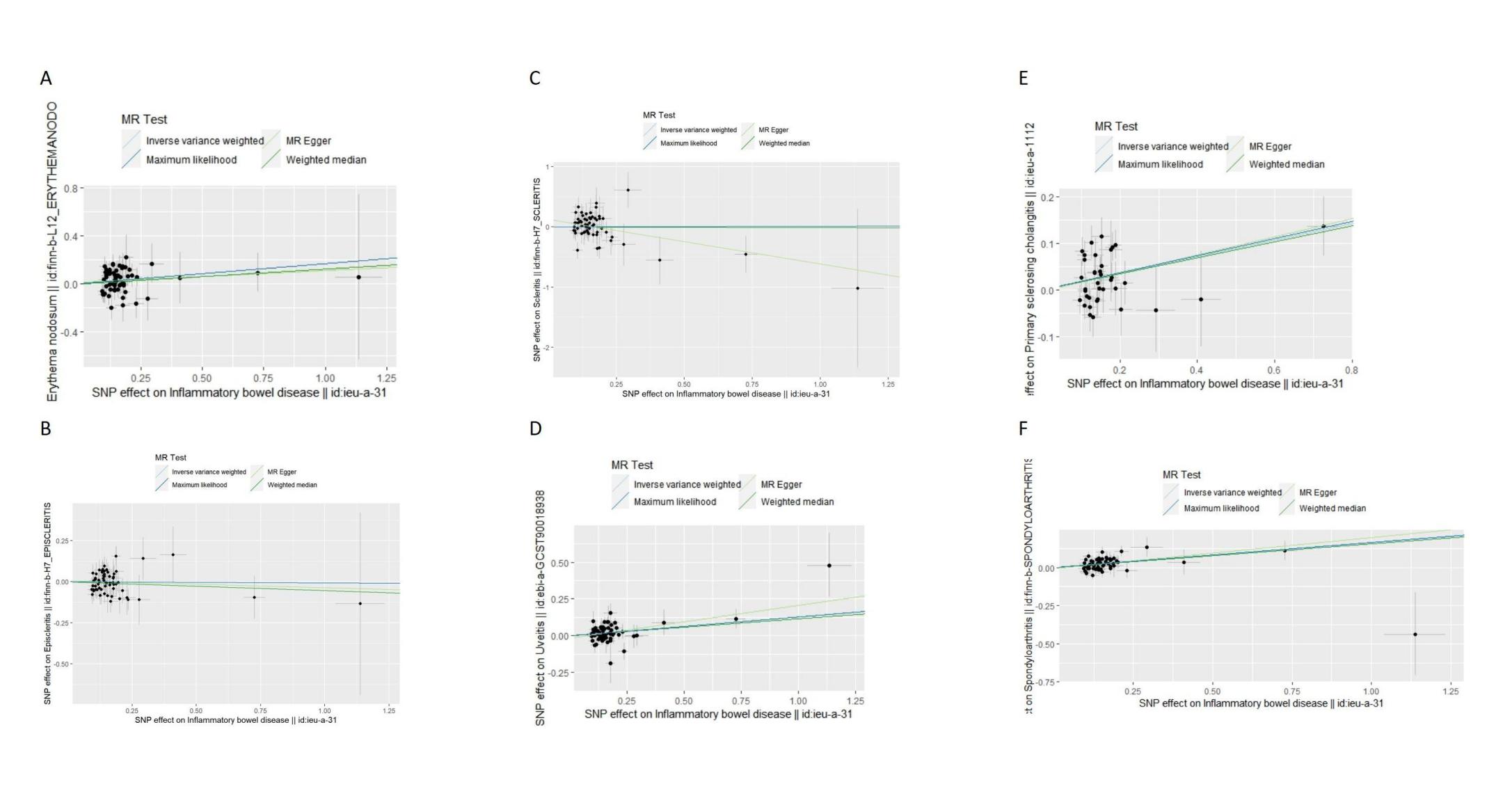


**Supplement Figure 2.** Funnel plots for genetically predicted IBD on EN (A), episcleritis (B), scleritis (C), uveitis (D), PSC (E),

and spondyloarthritis (F) in the initial practice.


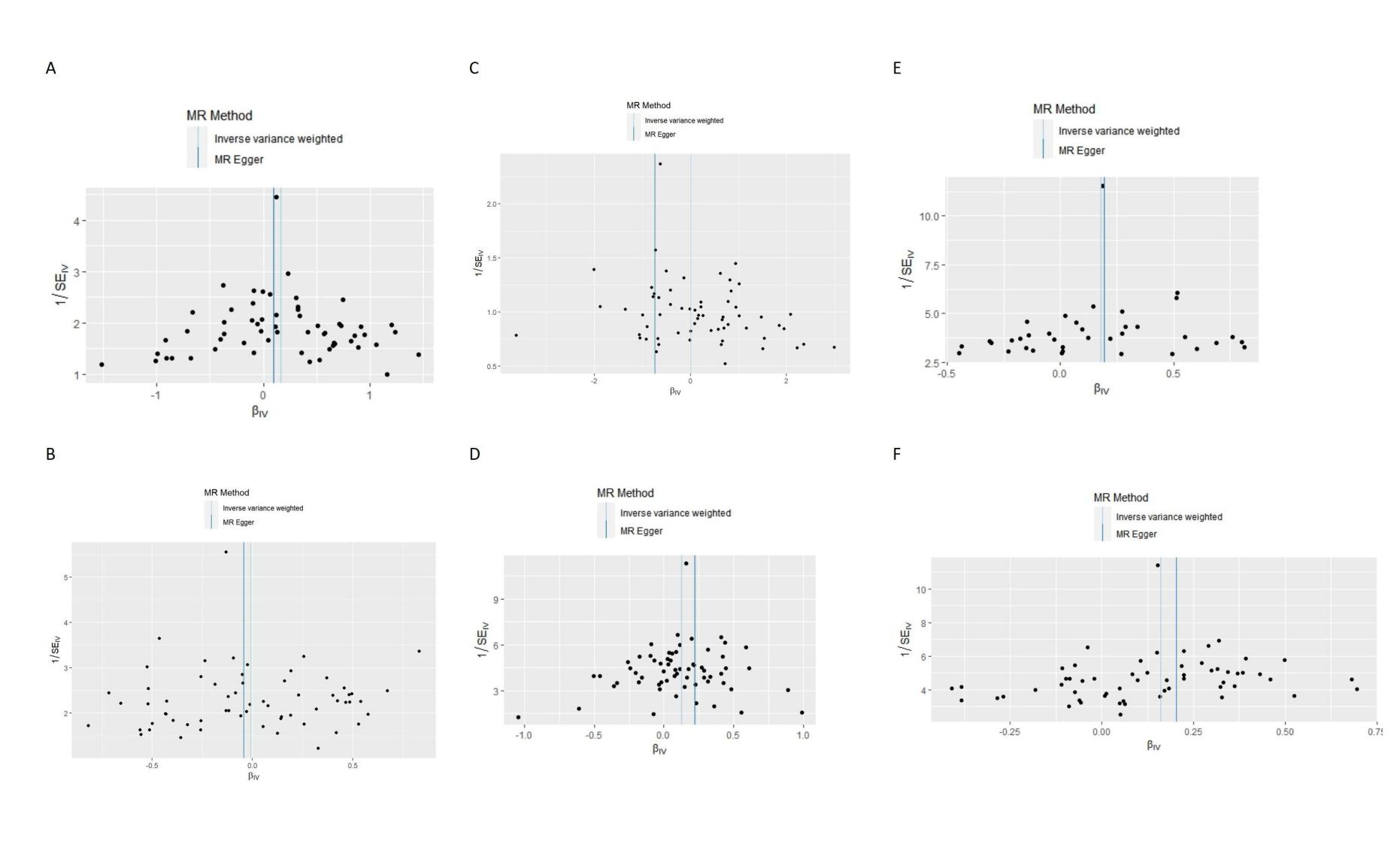


**Supplement Figure 3.** Leave-one-out plots for genetically predicted IBD on EN (A), episcleritis (B), scleritis (C), uveitis (D), PSC (E),

and spondyloarthritis (F) in the initial practice.


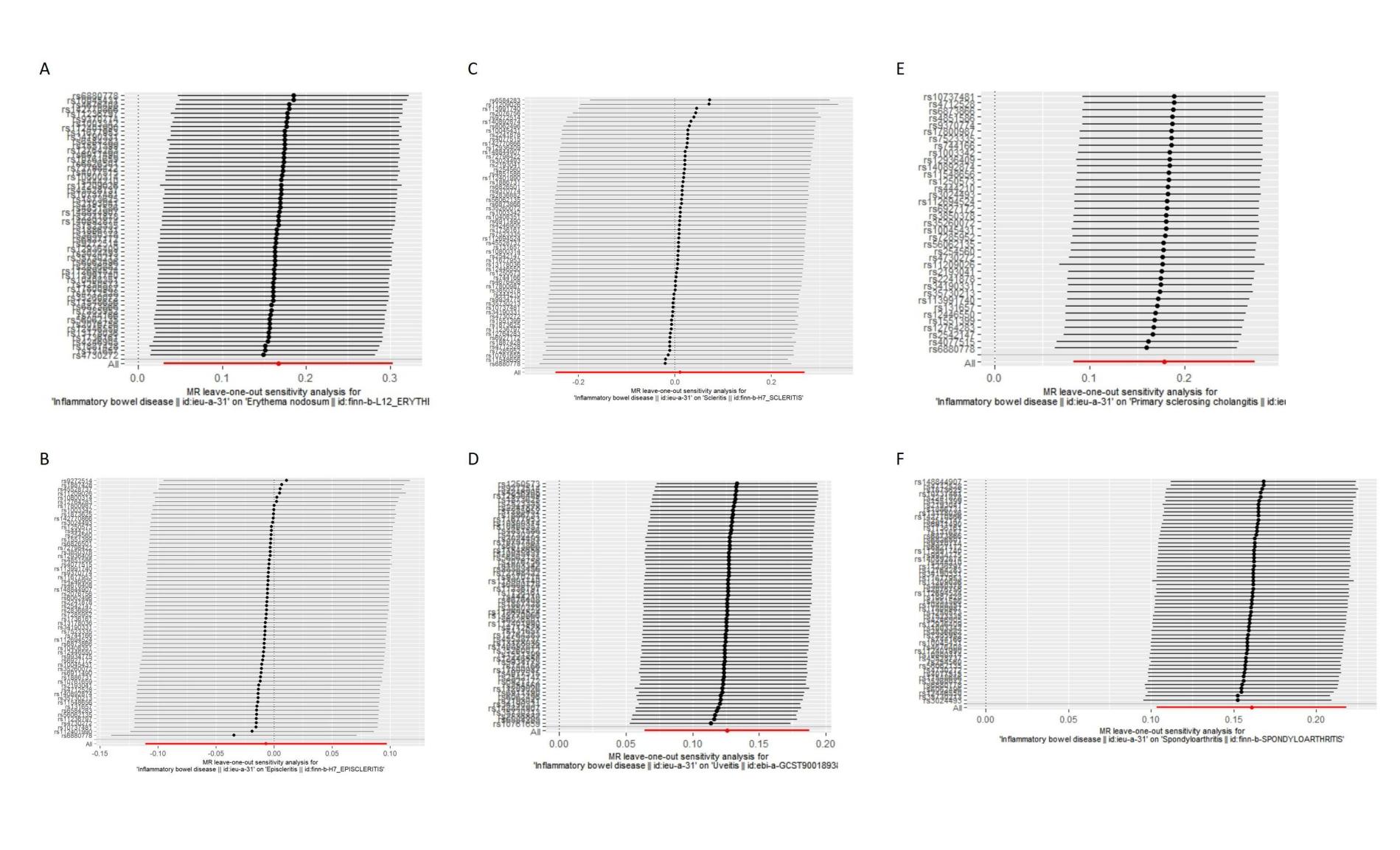


**Supplement Figure 4**. Scatter plots for genetically predicted IBD on EN (A), episcleritis (B), scleritis (C), uveitis (D), PSC (E),

and spondyloarthritis (F) in the replication practice.


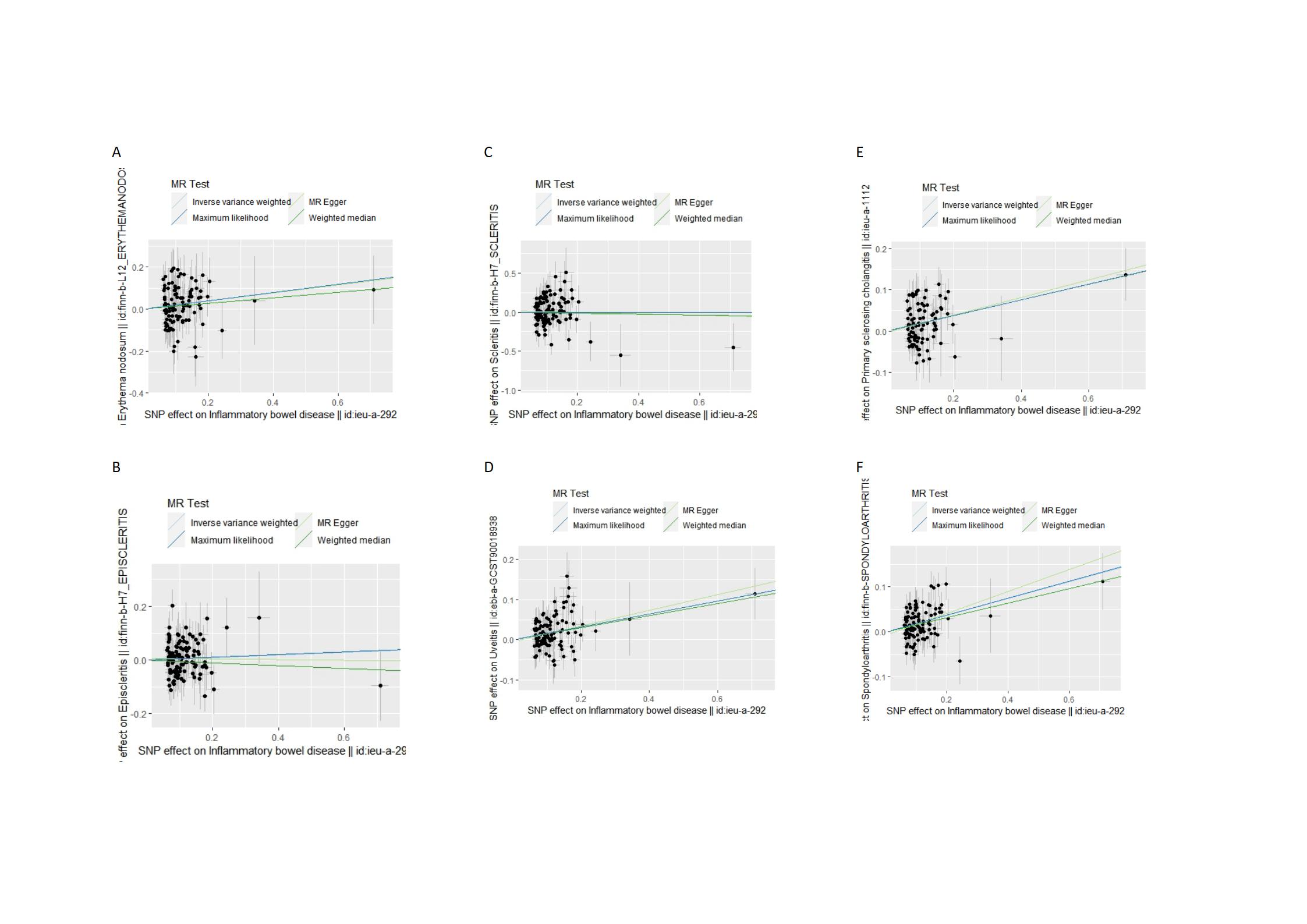


**Supplement Figure 5.** Funnel plots for genetically predicted IBD on EN (A), episcleritis (B), scleritis (C), uveitis (D), PSC (E),

and spondyloarthritis (F) in the replication practice.


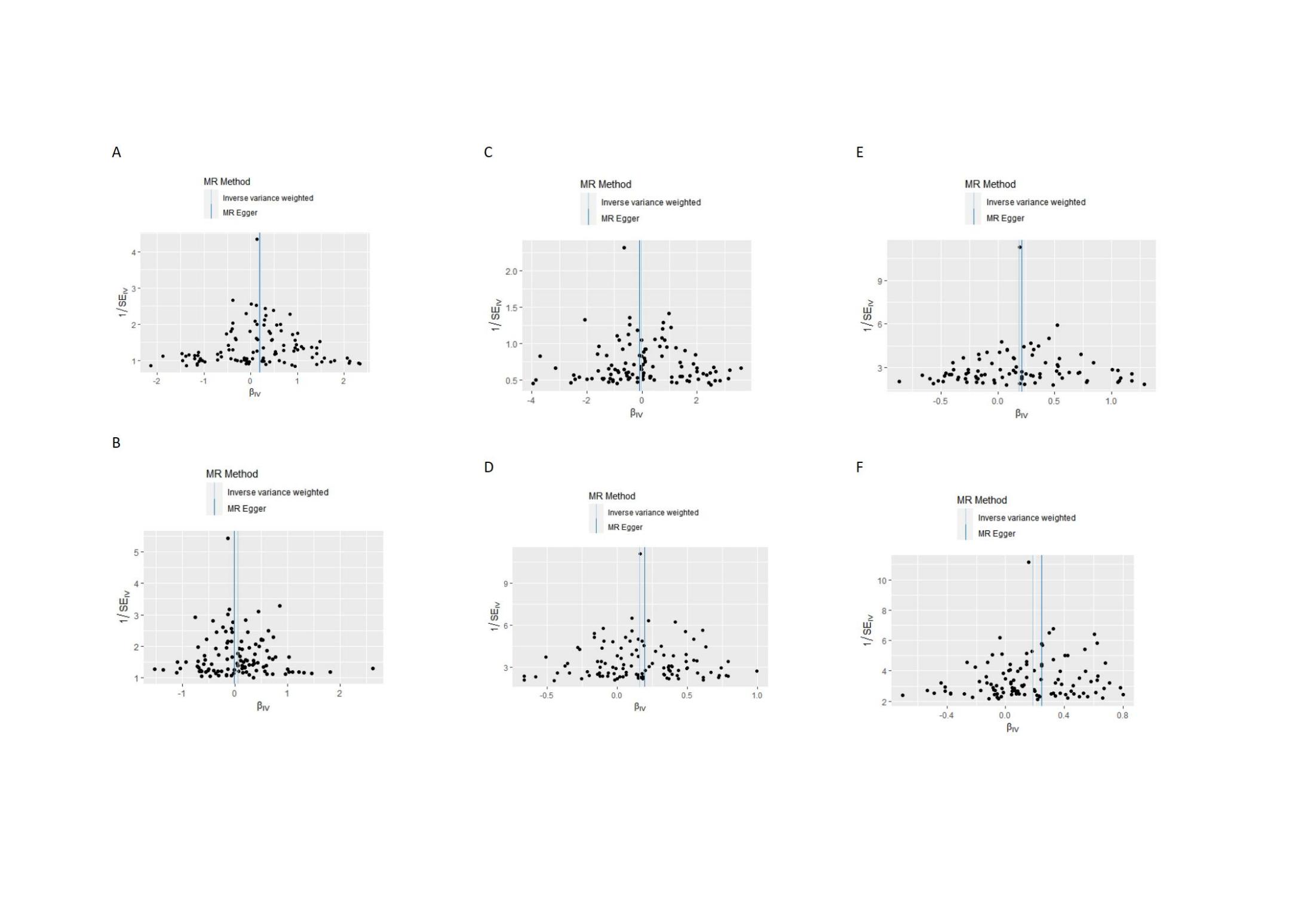


**Supplement Figure 6.** Leave-one-out plots for genetically predicted IBD on EN (A), episcleritis (B), scleritis (C), uveitis (D), PSC (E),

and spondyloarthritis (F) in the replication practice.


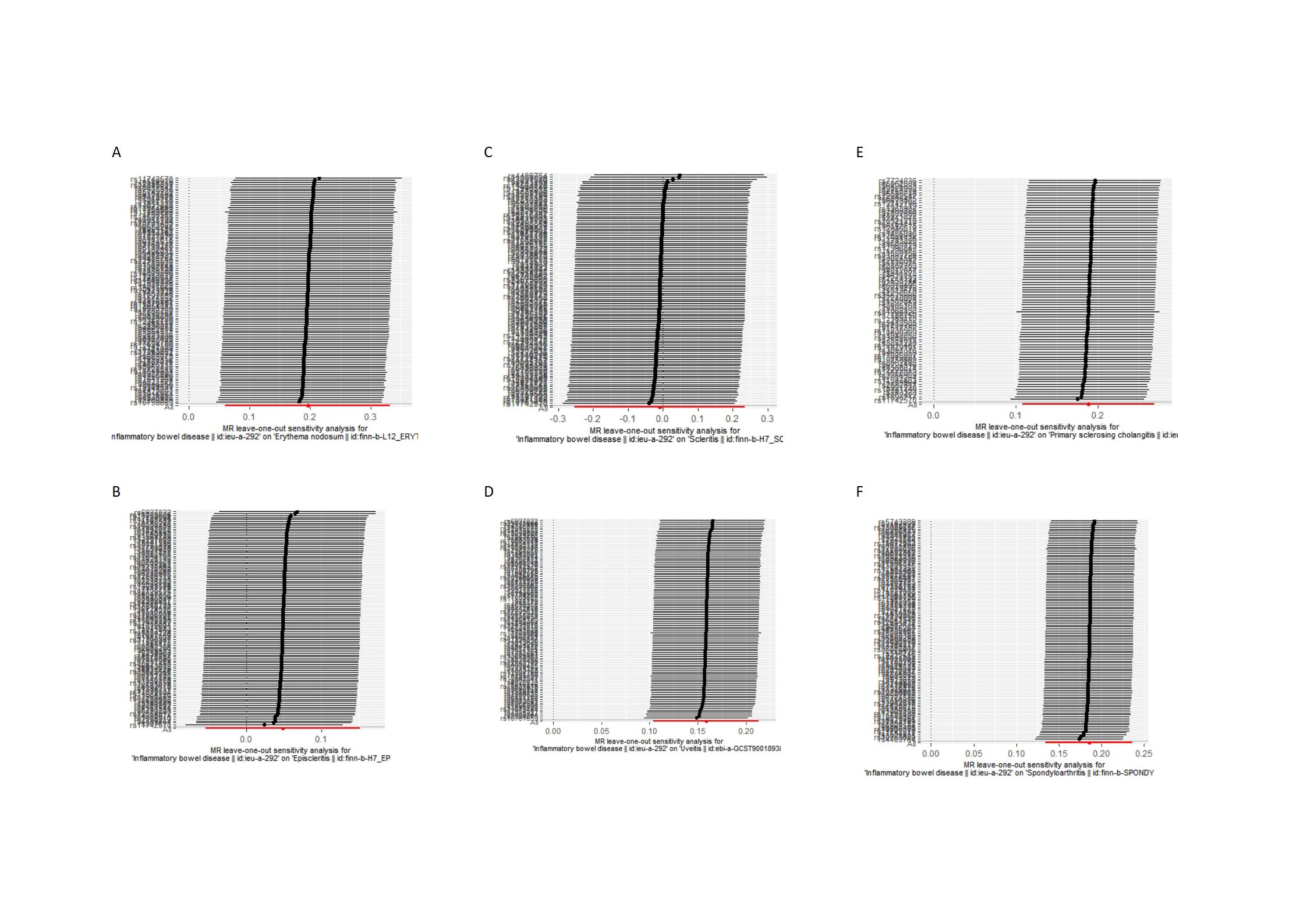


**Supplement Figure 7**. Scatter plots for genetically predicted UC on EN (A), episcleritis (B), scleritis (C), uveitis (D), PSC (E),

and spondyloarthritis (F) in the initial practice.


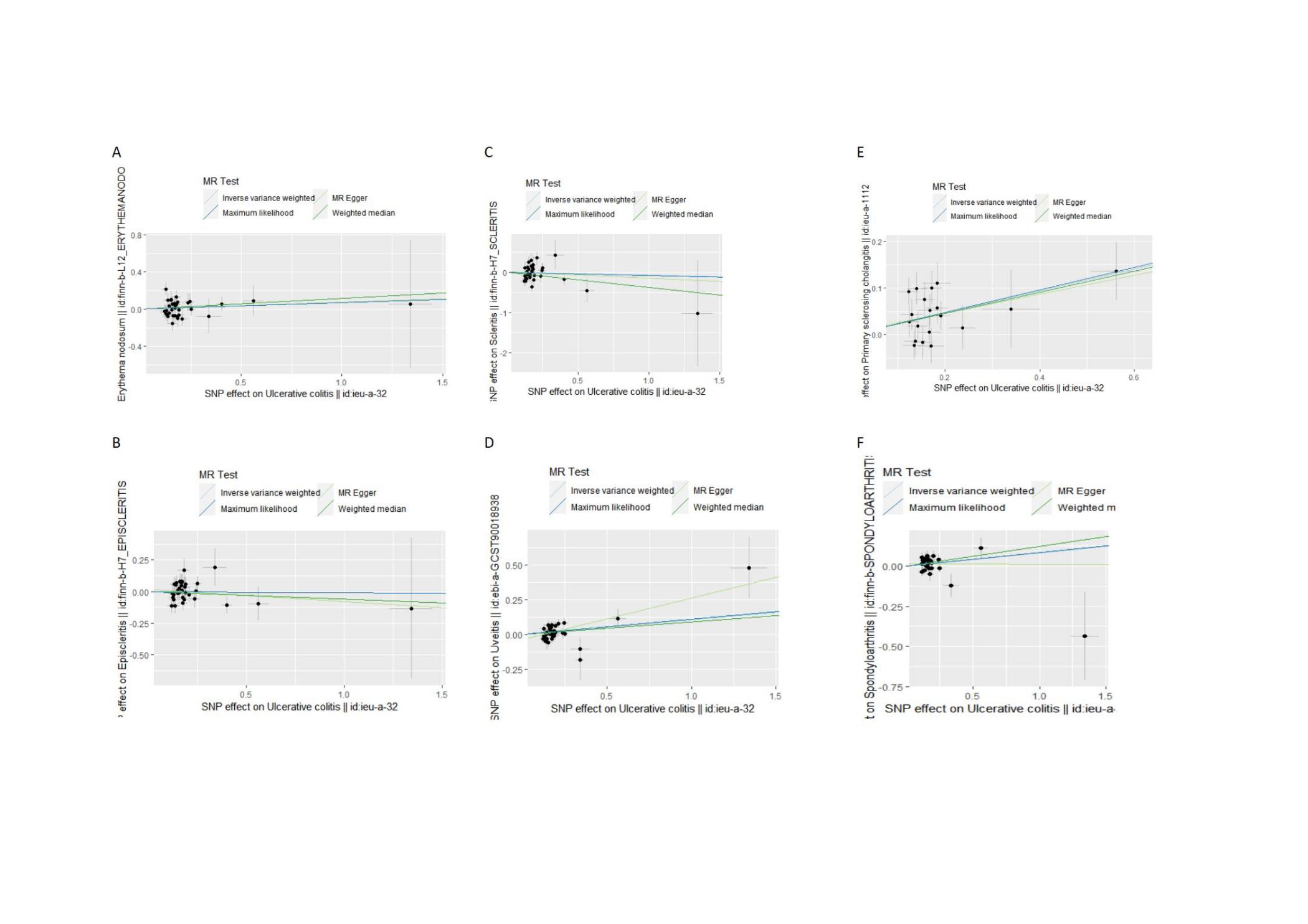


**Supplement Figure 8.** Funnel plots for genetically predicted UC on EN (A), episcleritis (B), scleritis (C), uveitis (D), PSC (E),

and spondyloarthritis (F) in the initial practice.


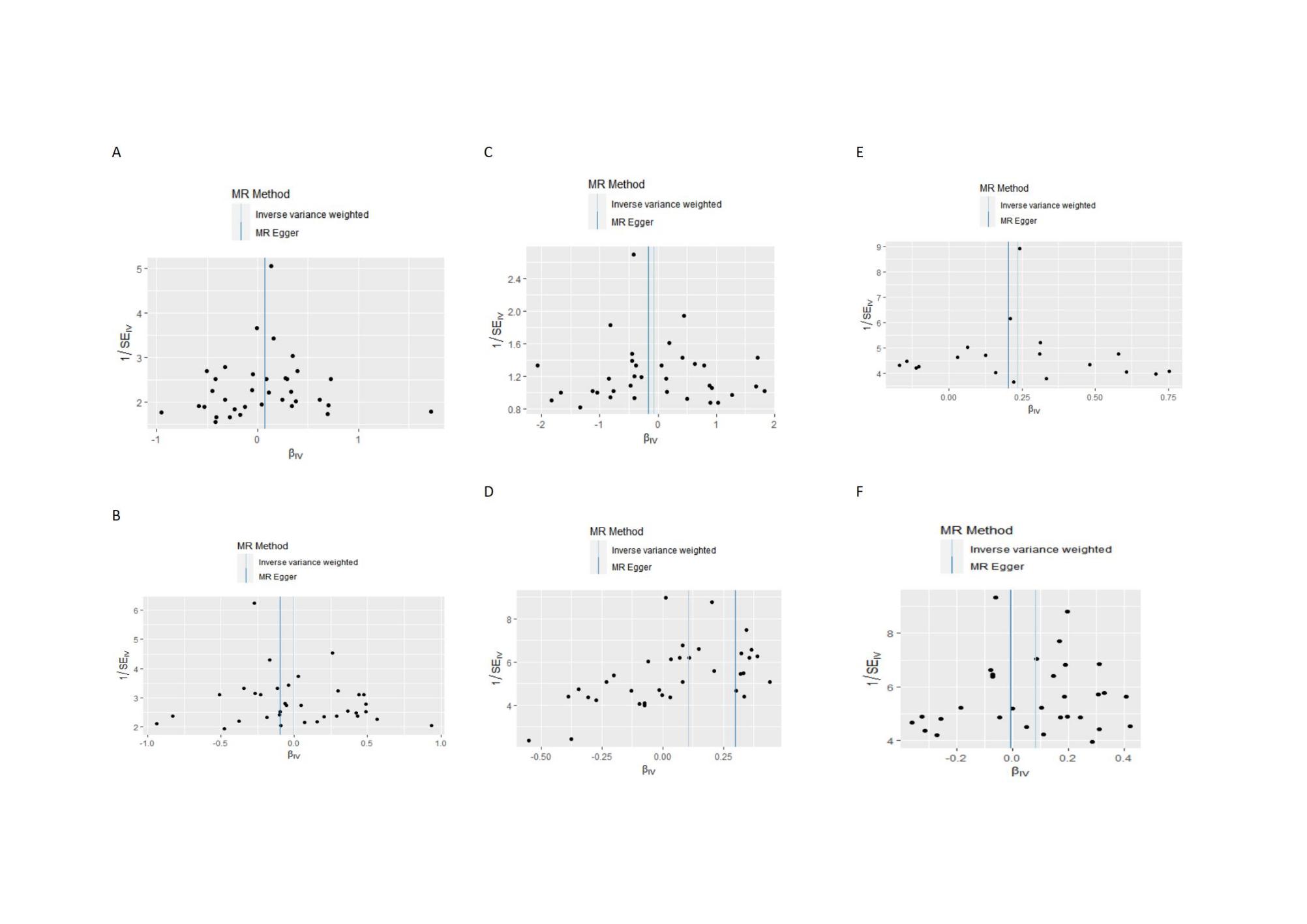


**Supplement Figure 9.** Leave-one-out plots for genetically predicted UC on EN (A), episcleritis (B), scleritis (C), uveitis (D), PSC (E),

and spondyloarthritis (F) in the initial practice.


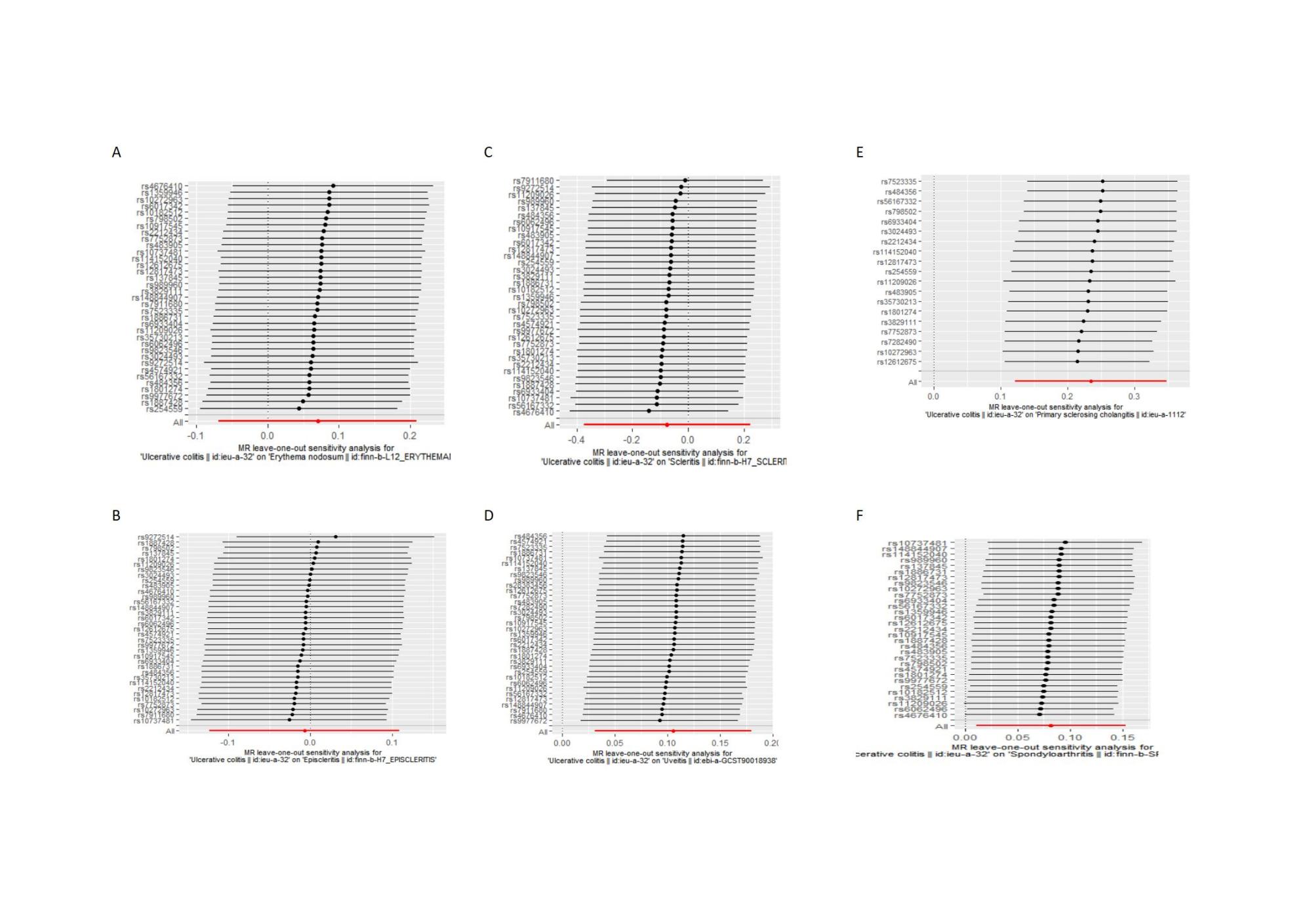


**Supplement Figure 10**. Scatter plots for genetically predicted UC on EN (A), episcleritis (B), scleritis (C), uveitis (D), PSC (E),

and spondyloarthritis (F) in the replication practice.

**
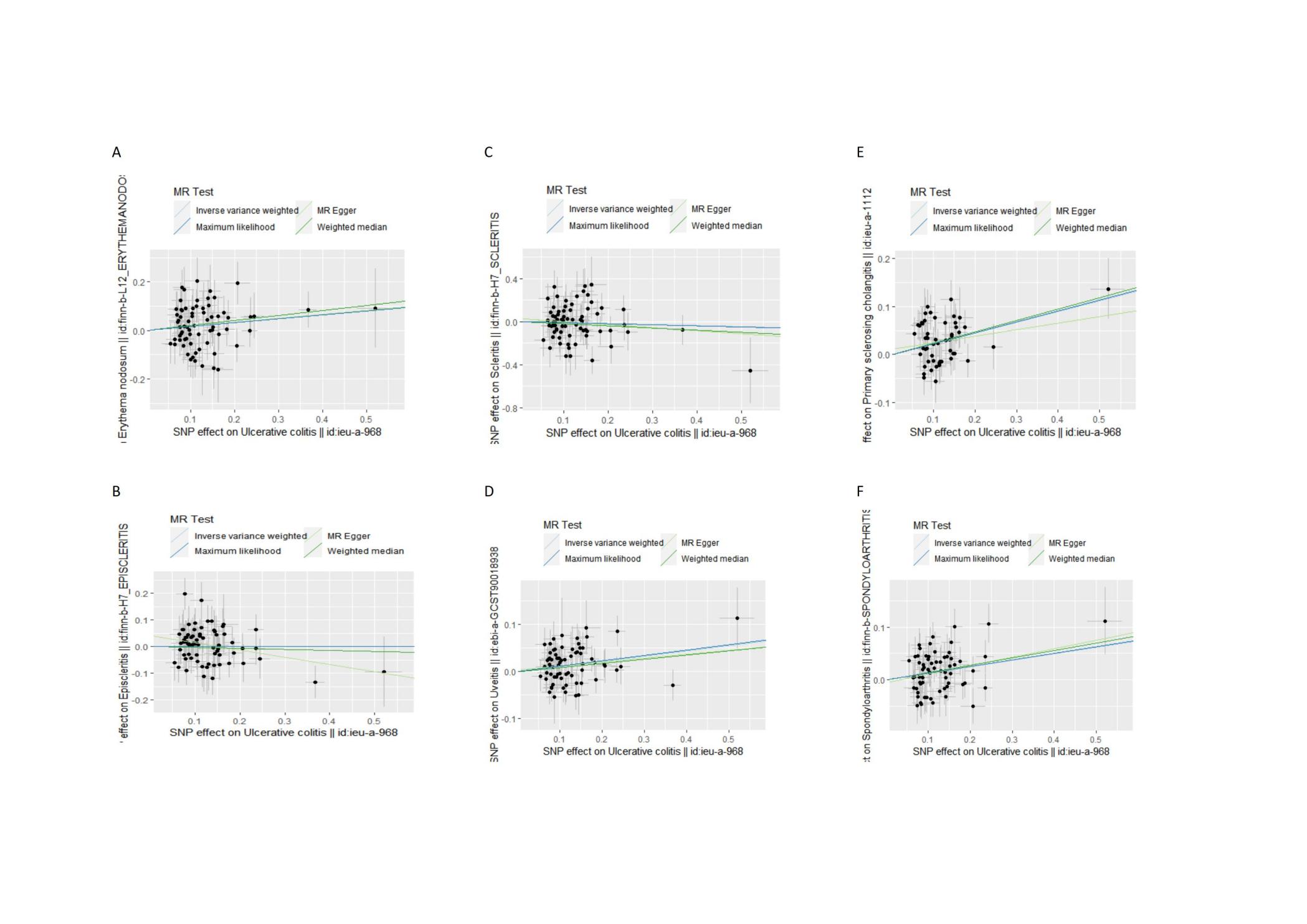
**

**Supplement Figure 11.** Funnel plots for genetically predicted UC on EN (A), episcleritis (B), scleritis (C), uveitis (D), PSC (E),

and spondyloarthritis (F) in the replication practice.

**
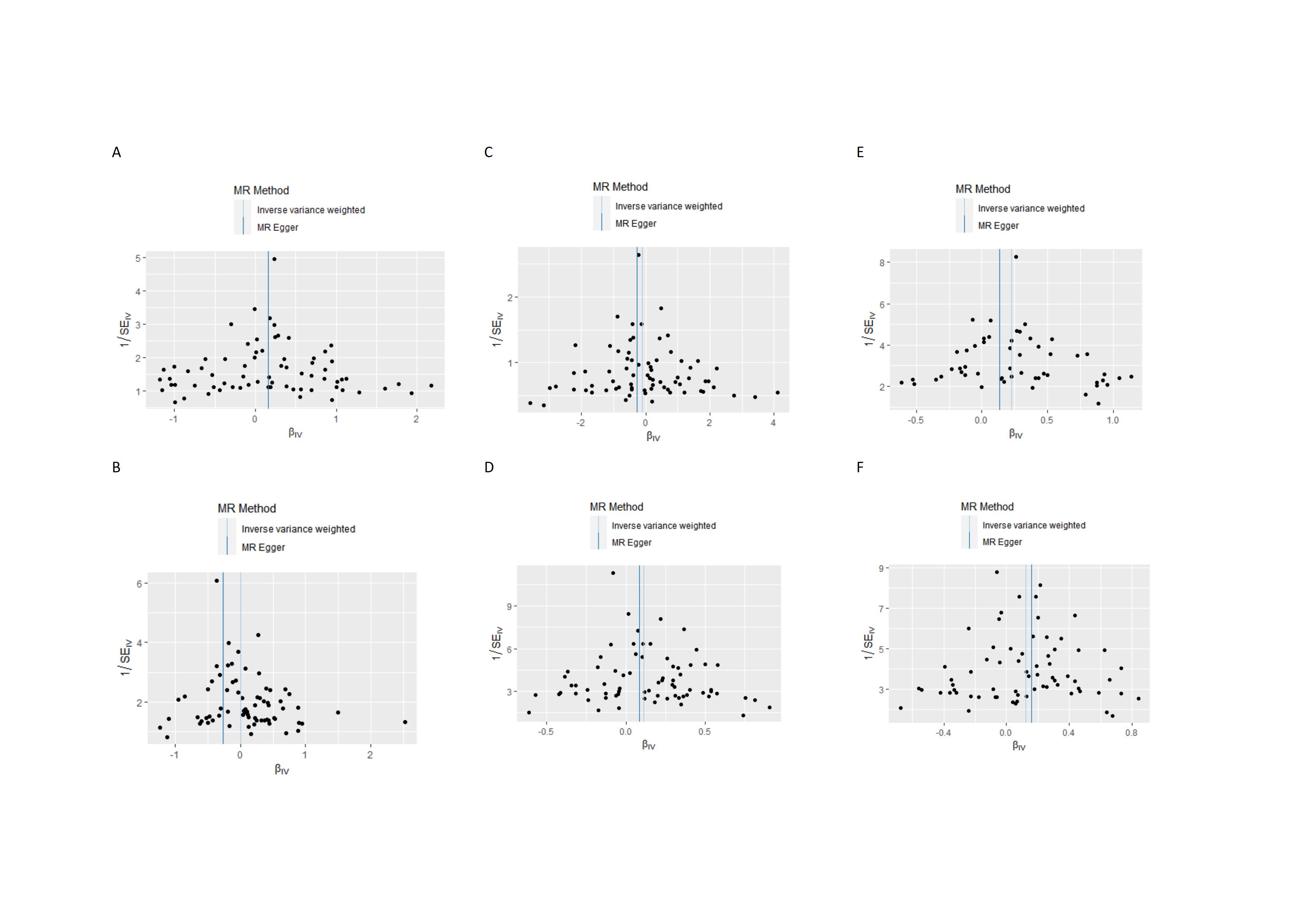
**

**Supplement Figure 12.** Leave-one-out plots for genetically predicted UC on EN (A), episcleritis (B), scleritis (C), uveitis (D), PSC (E),

and spondyloarthritis (F) in the replication practice.


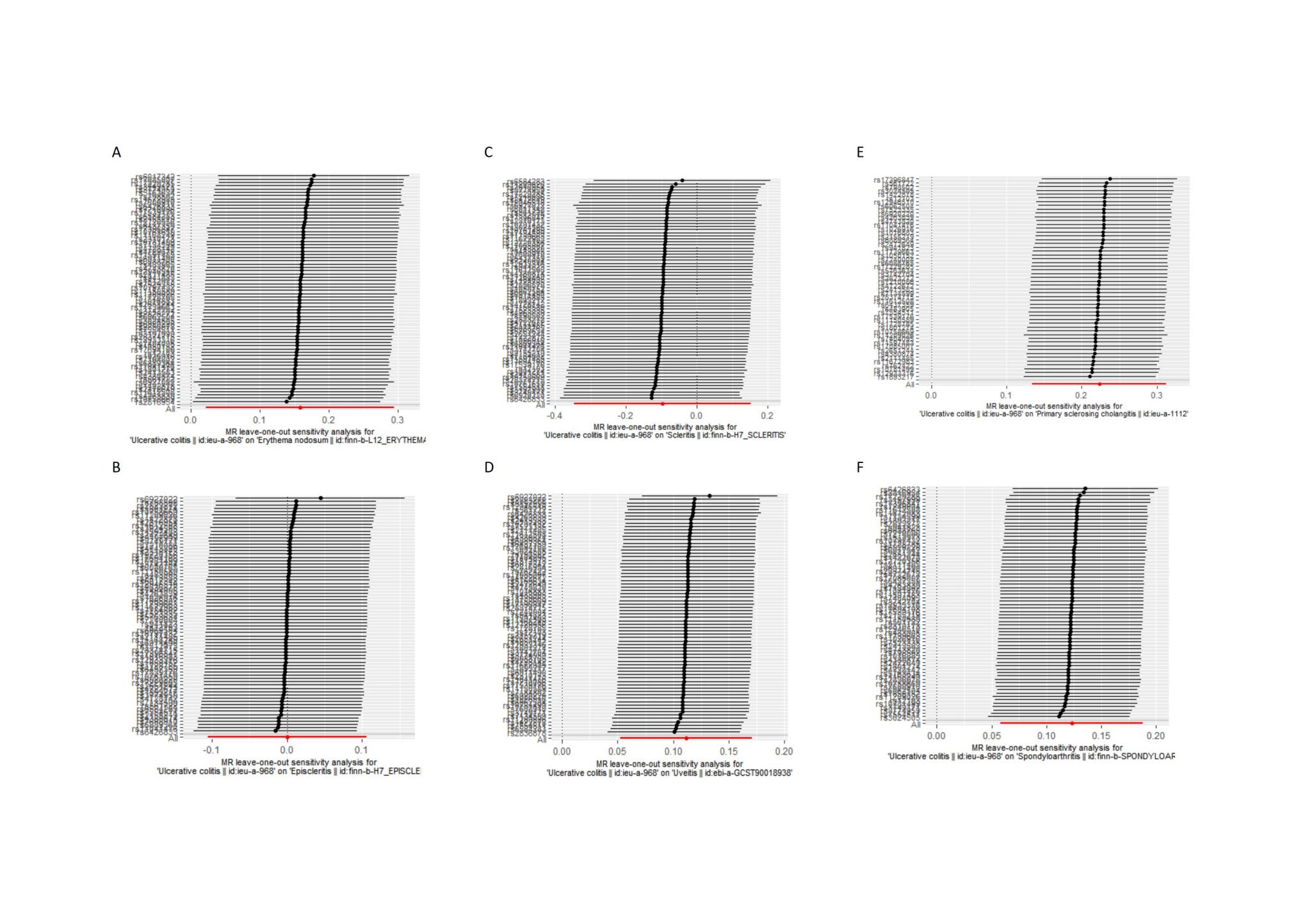


**Supplement Figure 13**. Scatter plots for genetically predicted CD on EN (A), episcleritis (B), scleritis (C), uveitis (D), PSC (E),

and spondyloarthritis (F) in the initial practice.


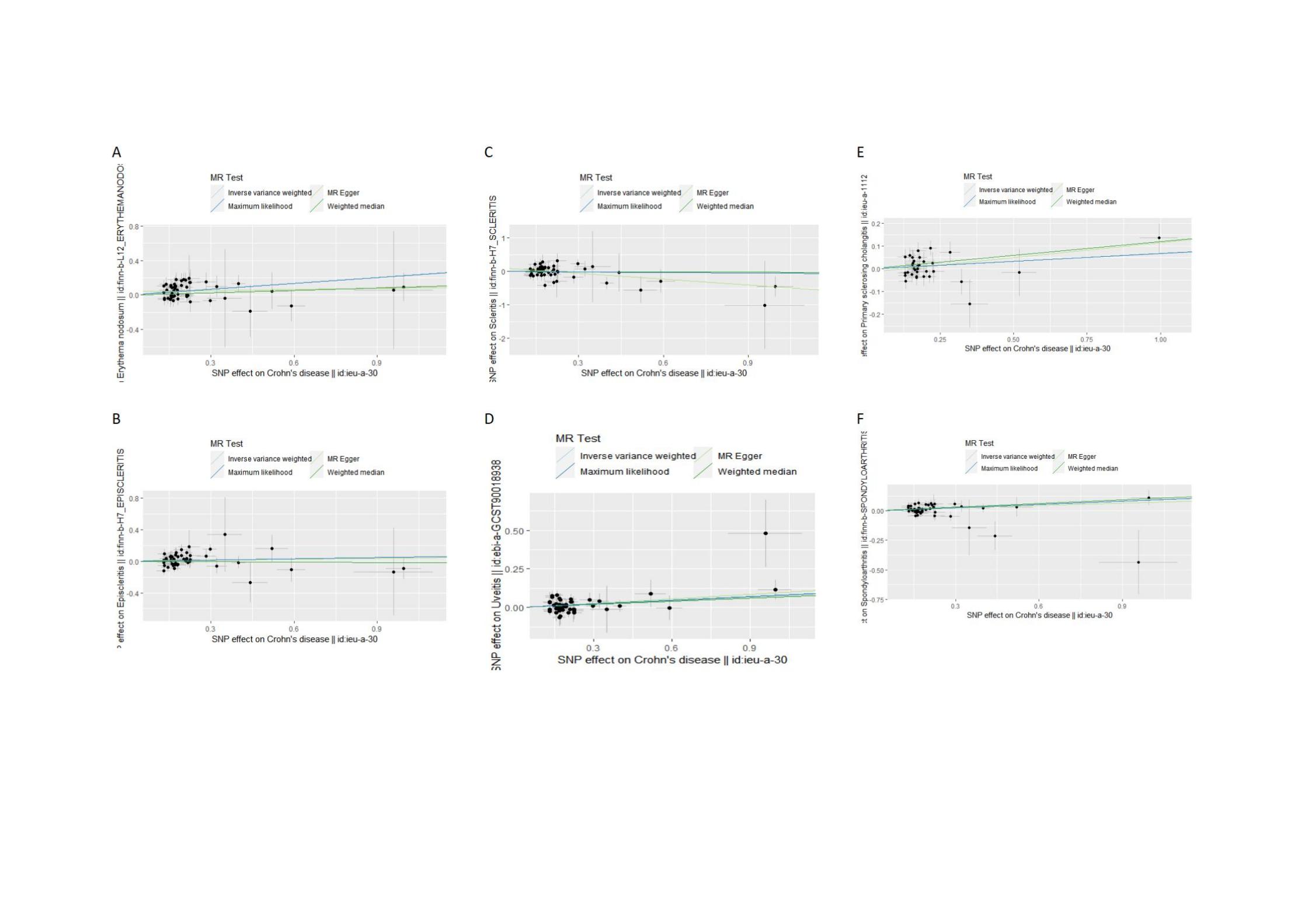


**Supplement Figure 14.** Funnel plots for genetically predicted CD on EN (A), episcleritis (B), scleritis (C), uveitis (D), PSC (E),

and spondyloarthritis (F) in the initial practice.


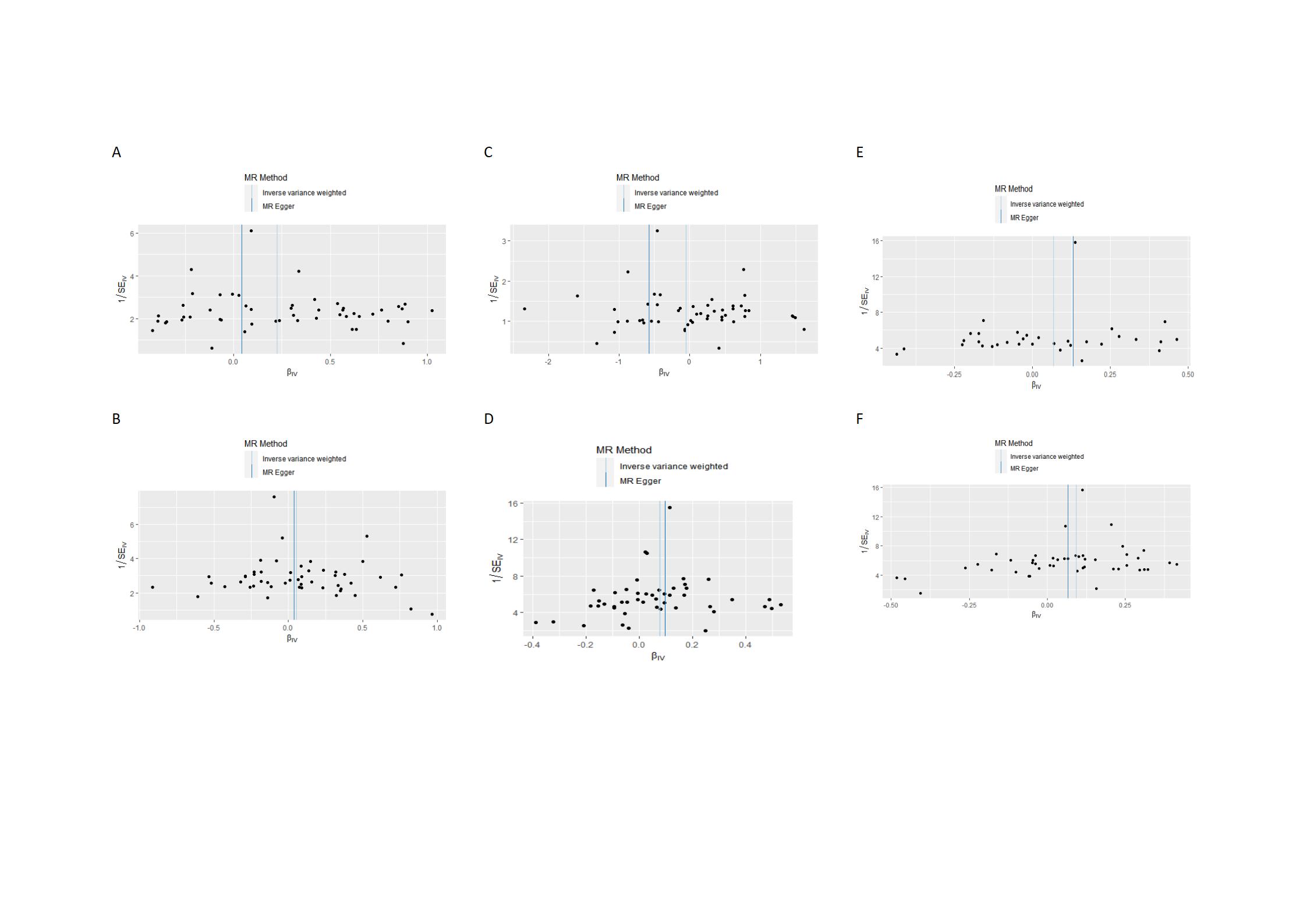


**Supplement Figure 15.** Leave-one-out plots for genetically predicted CD on EN (A), episcleritis (B), scleritis (C), uveitis (D), PSC (E),

and spondyloarthritis (F) in the initial practice.


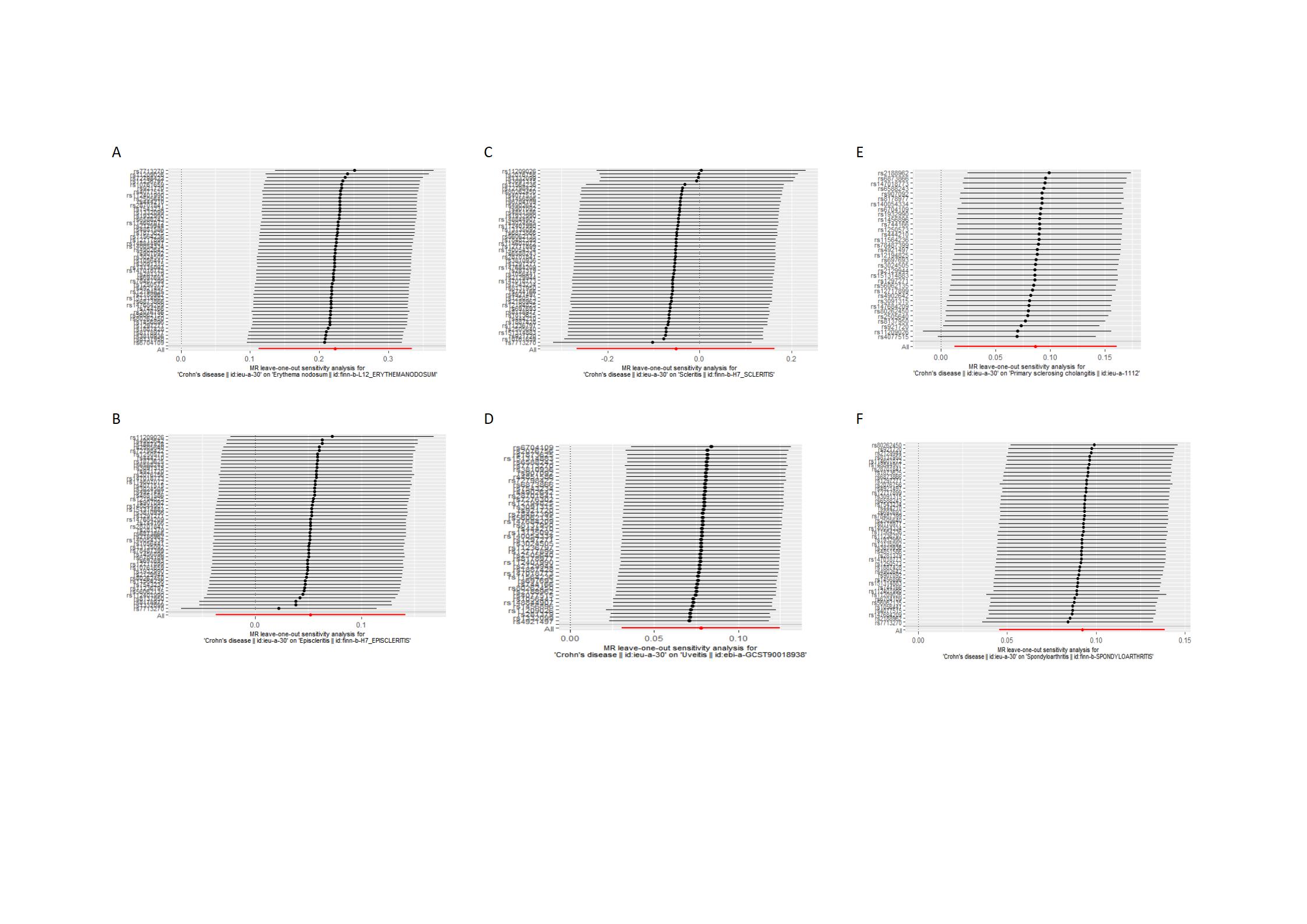


**Supplement Figure 16**. Scatter plots for genetically predicted CD on EN (A), episcleritis (B), scleritis (C), uveitis (D), PSC (E),

and spondyloarthritis (F) in the replication practice.


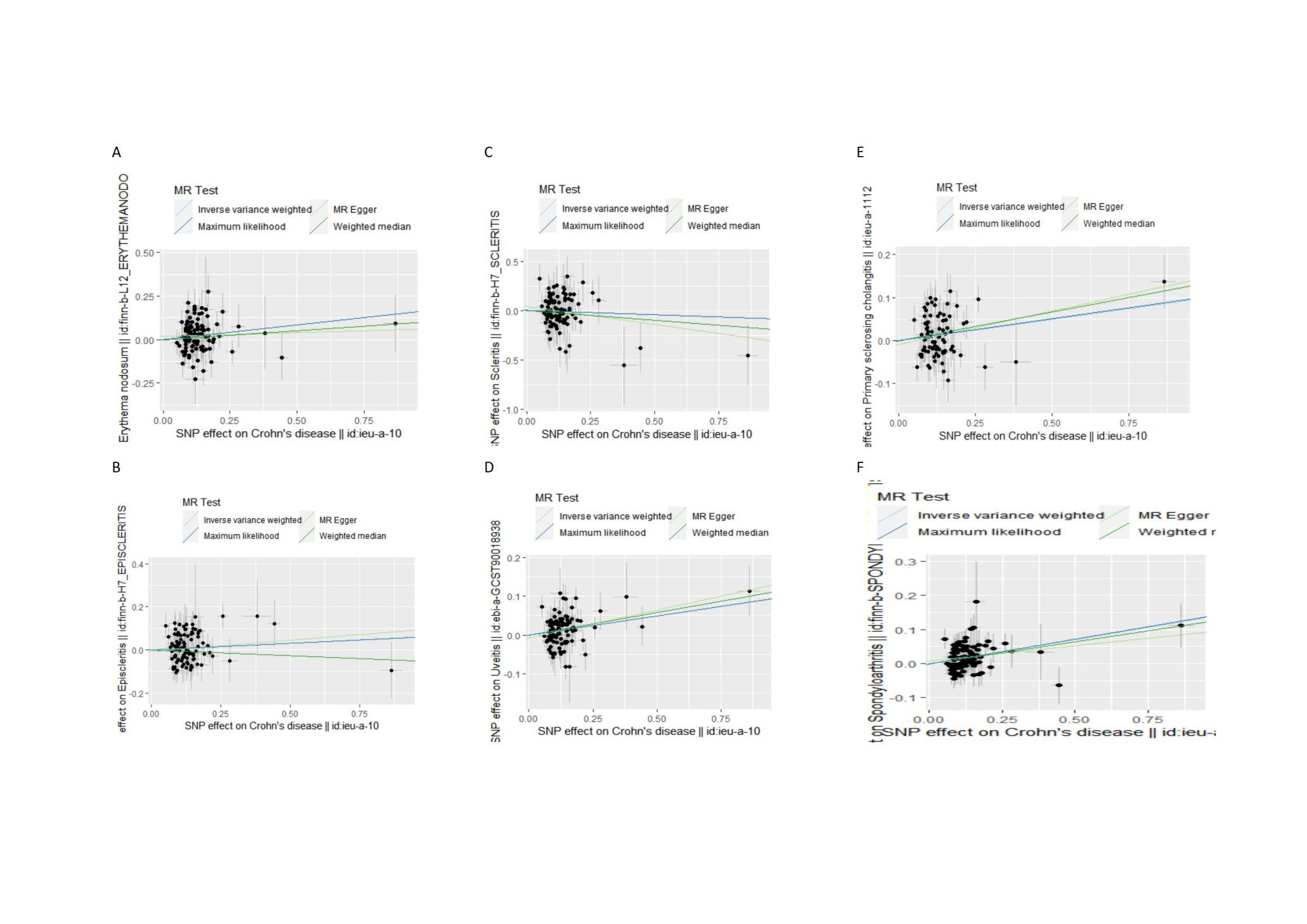


**Supplement Figure 17.** Funnel plots for genetically predicted CD on EN (A), episcleritis (B), scleritis (C), uveitis (D), PSC (E),

and spondyloarthritis (F) in the replication practice.


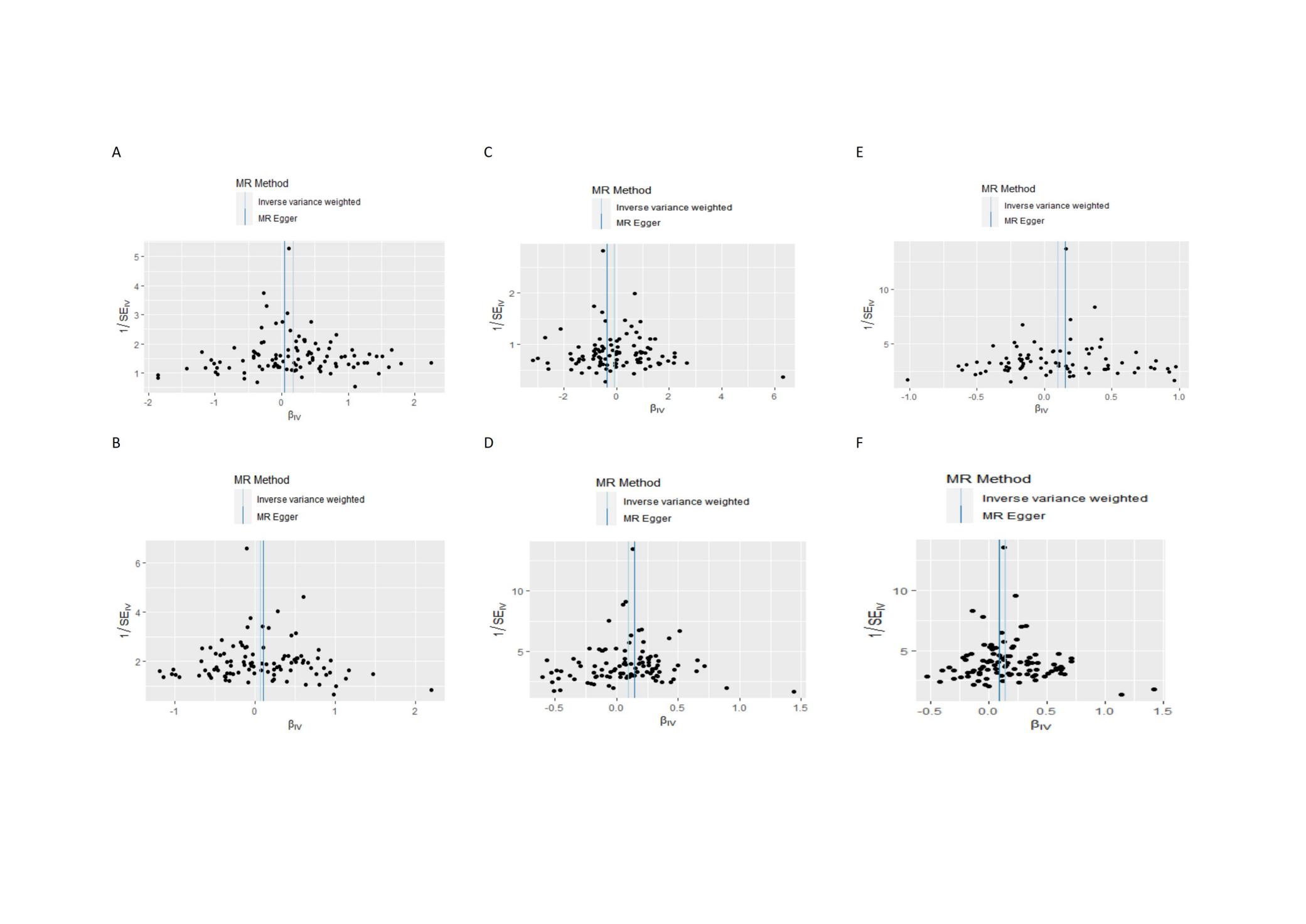


**Supplement Figure 18.** Leave-one-out plots for genetically predicted CD on EN (A), episcleritis (B), scleritis (C), uveitis (D), PSC (E),

and spondyloarthritis (F) in the replication practice.


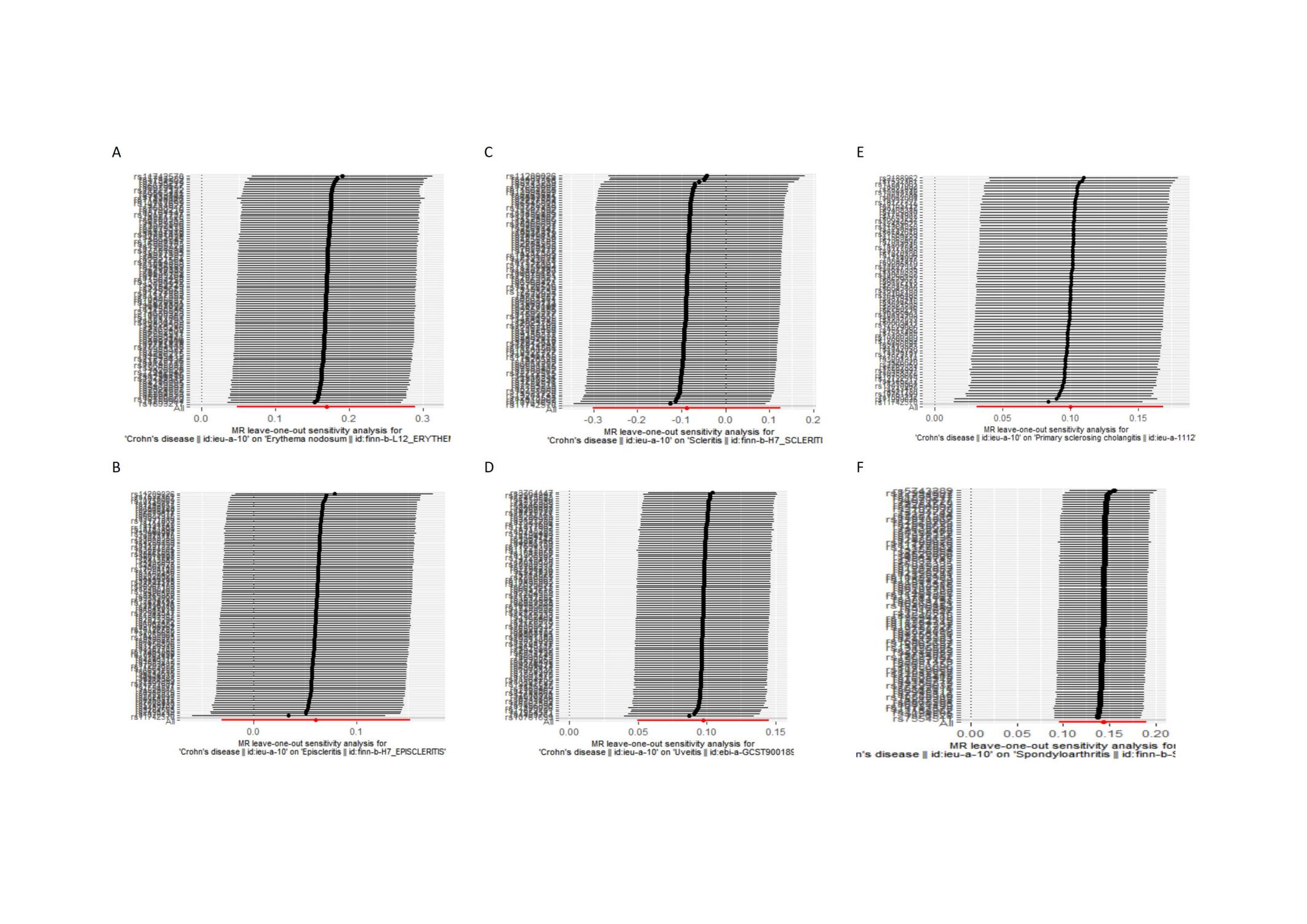


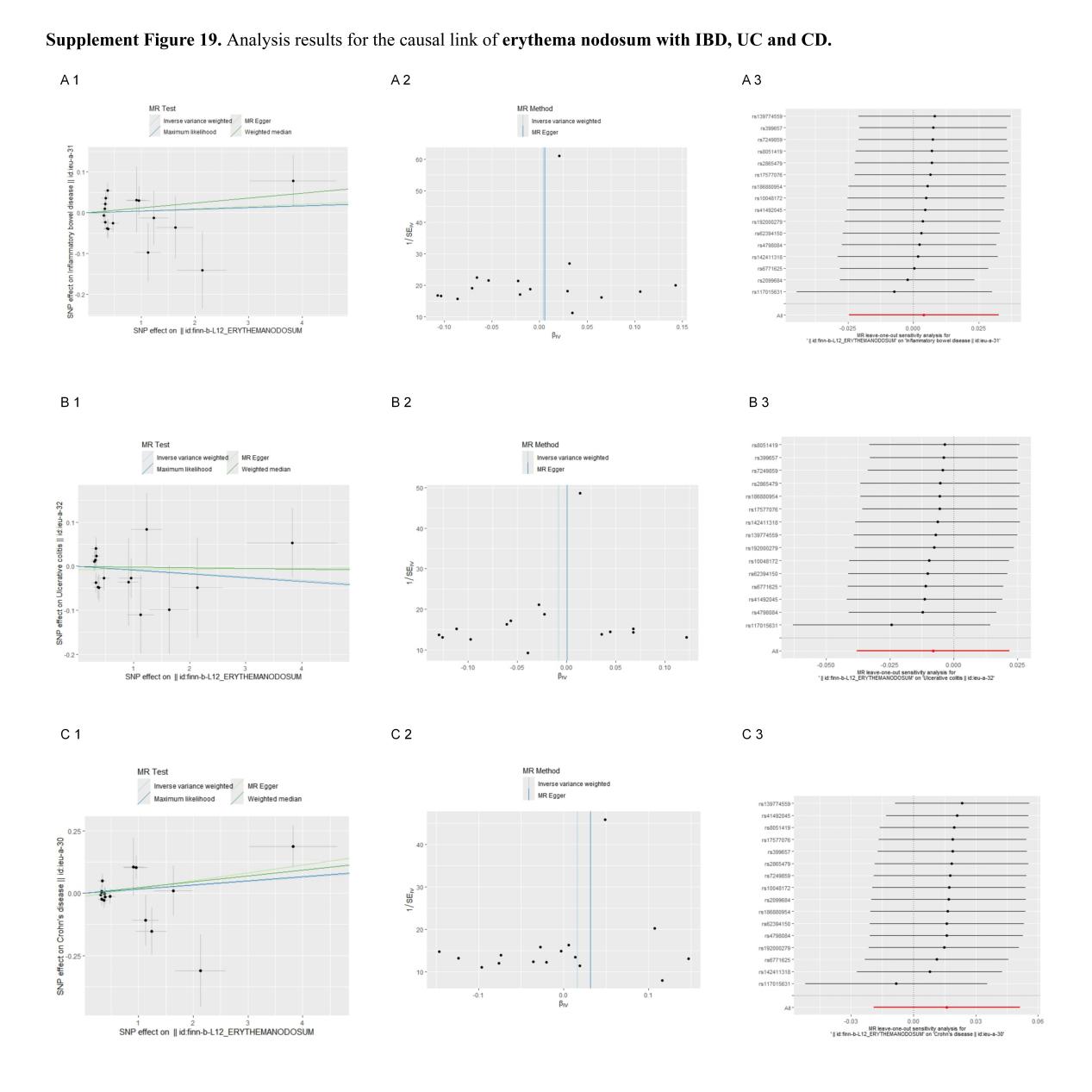


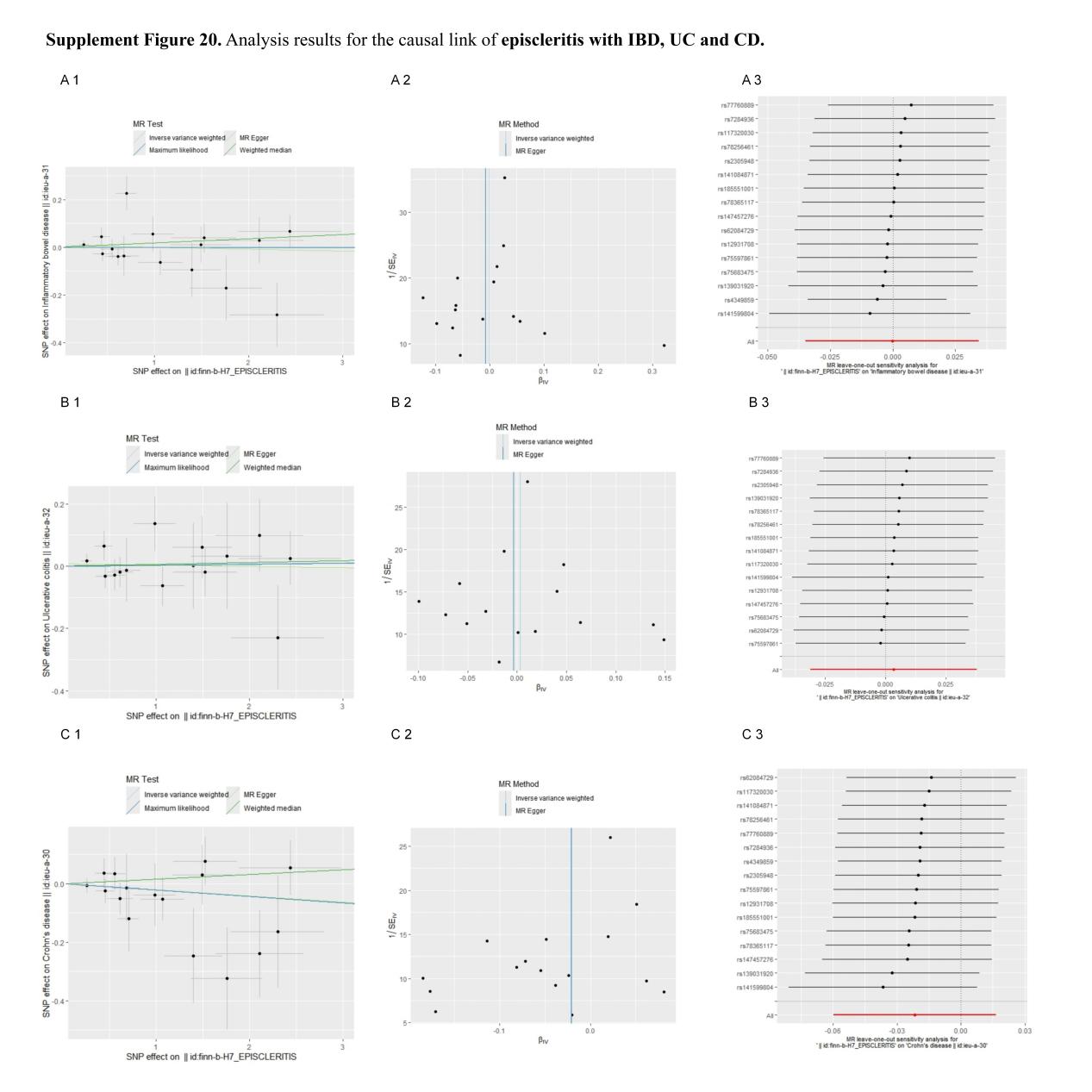


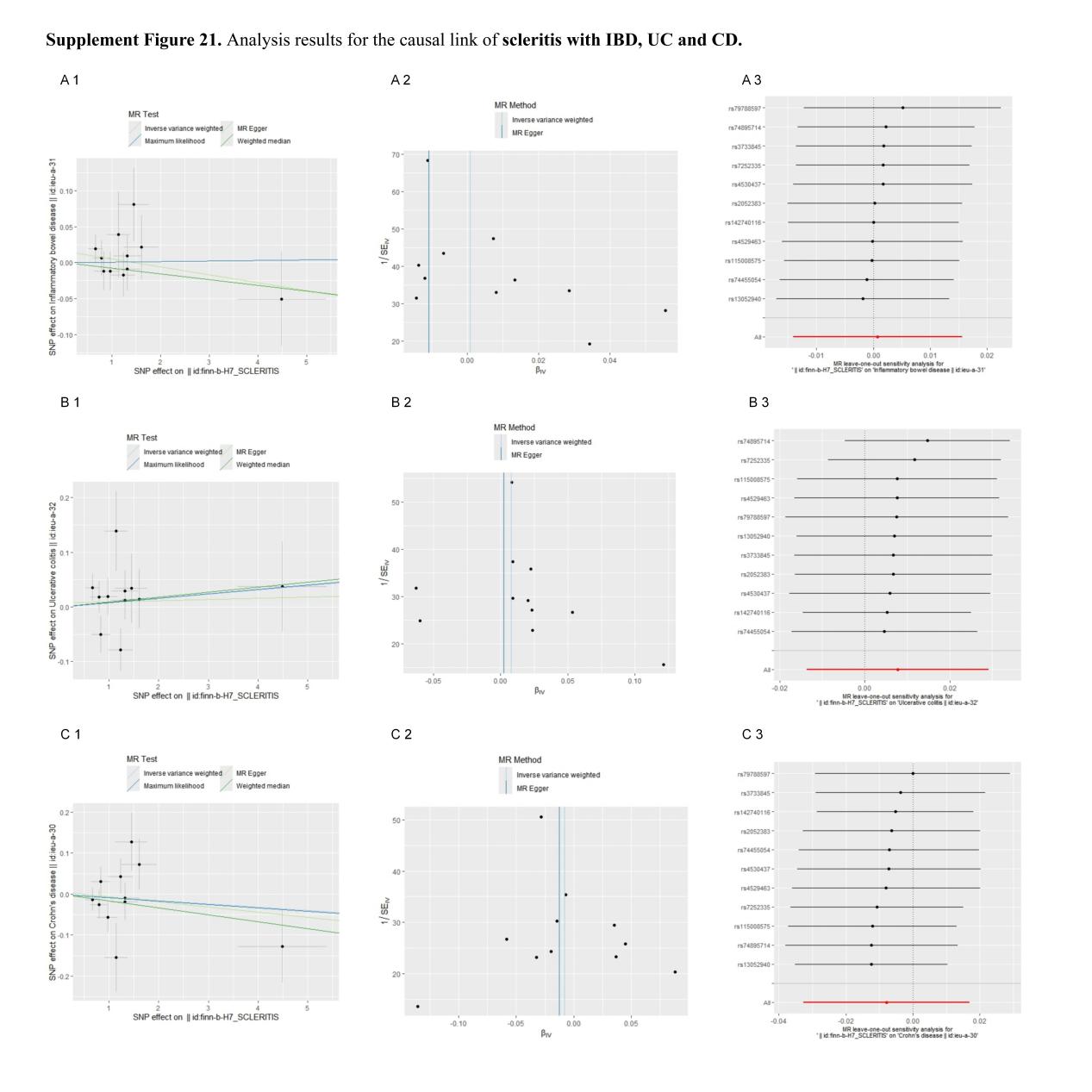


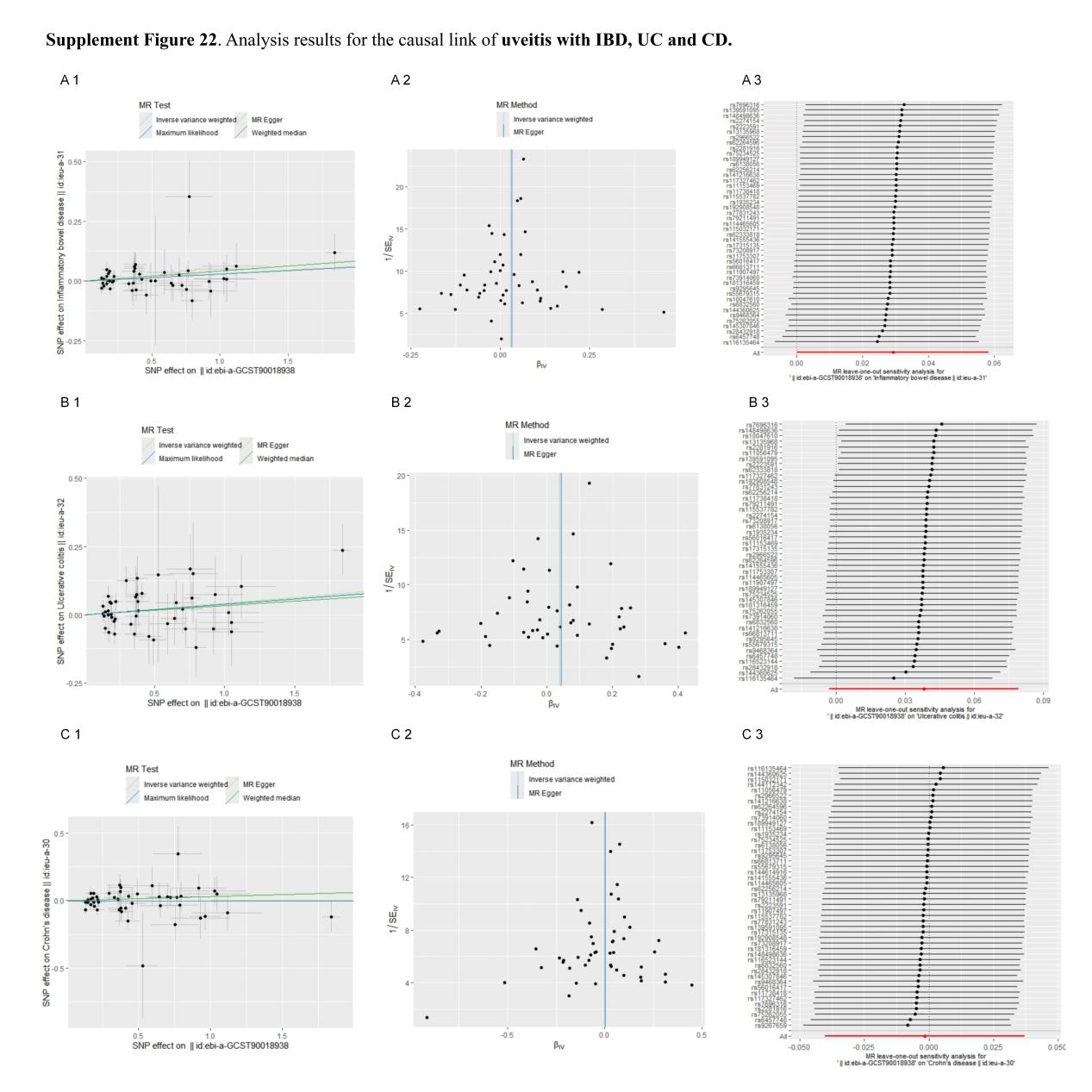

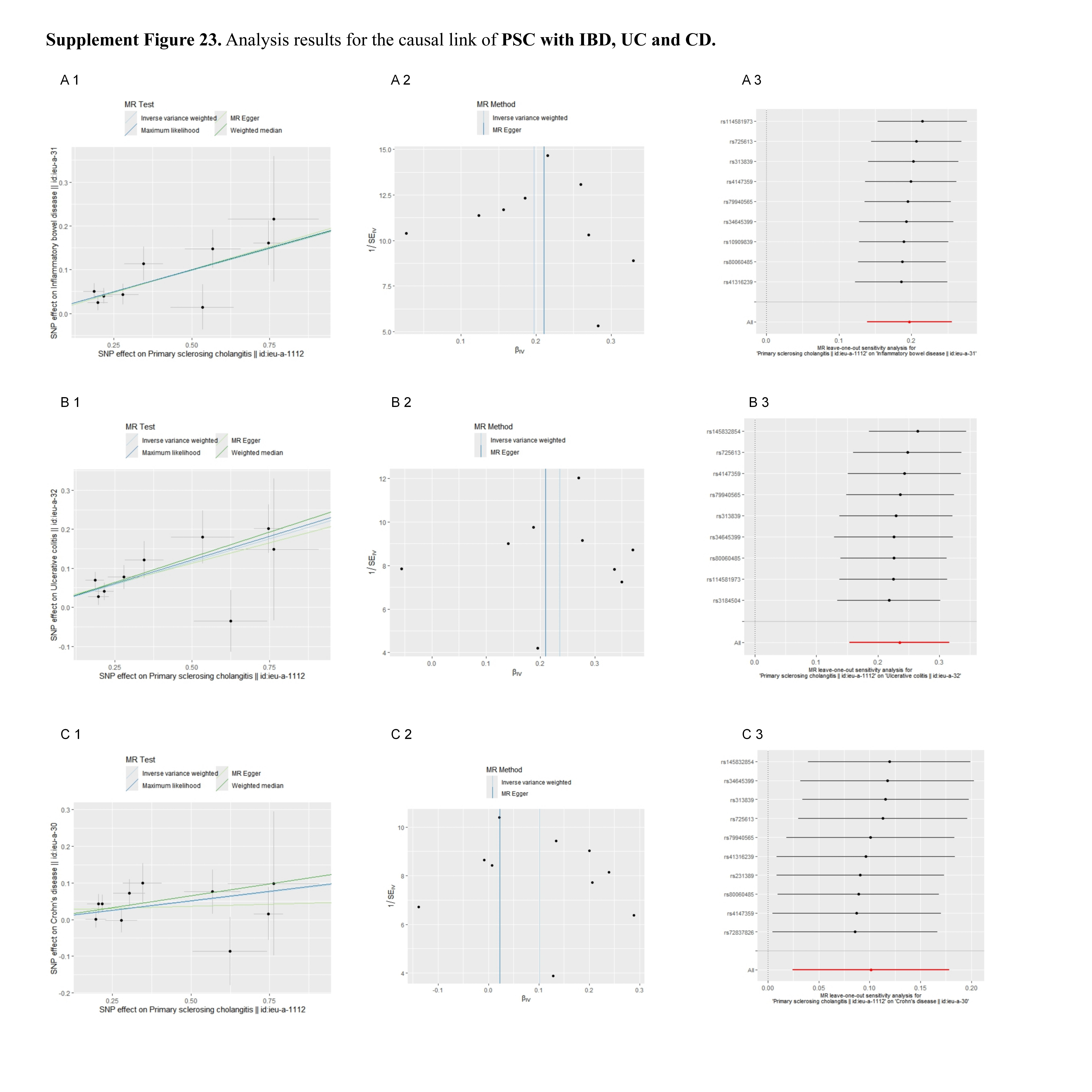

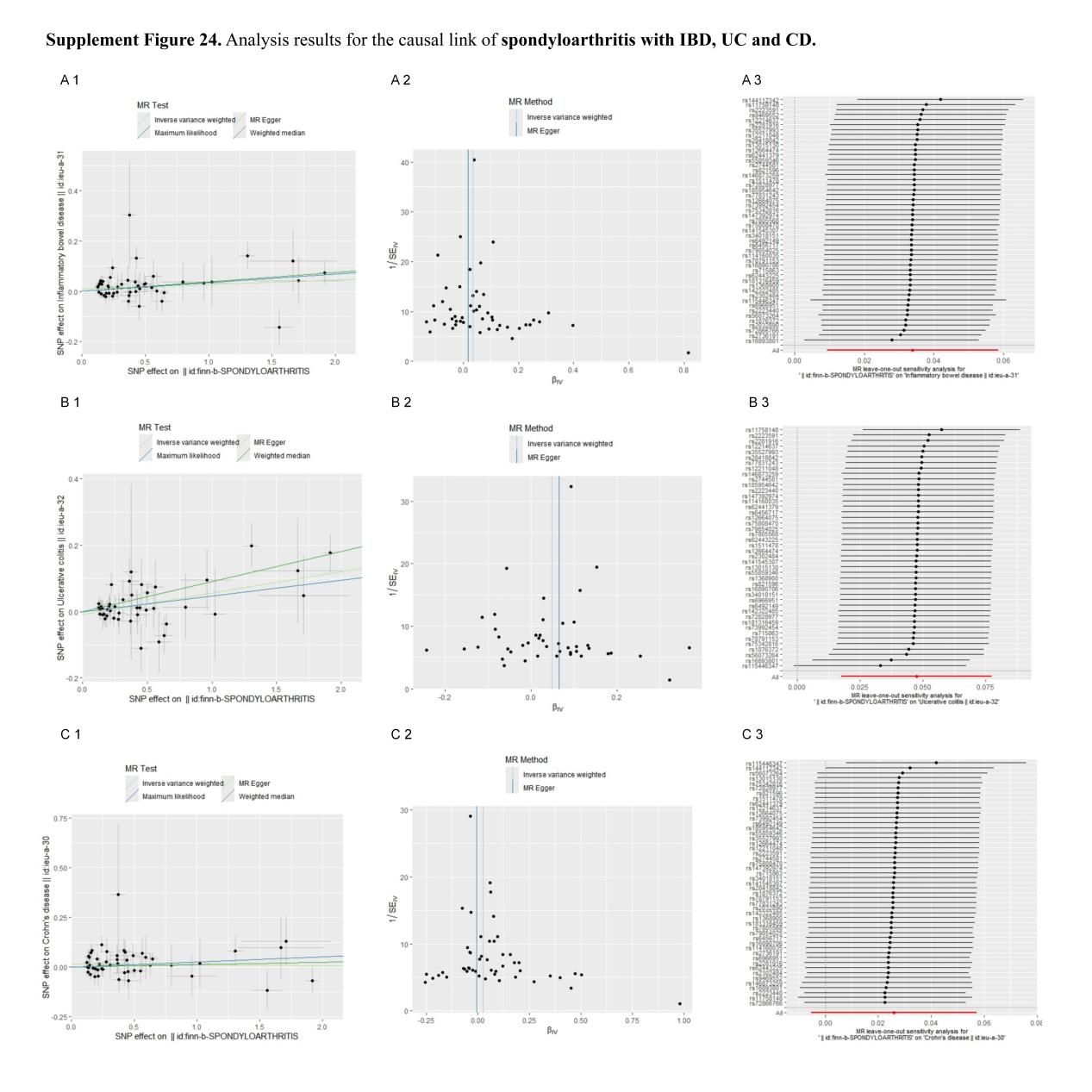

Supplement: Supplementary file 2 — Supplementary Material 2 [file 12876_2024_3566_MOESM2_ESM.docx]
