# Supplementary material for: The causal impact of genetically predicted inflammatory bowel disease on extraintestinal manifestations: a mendelian randomization study
Source: BMC Gastroenterol. 2025 Mar 4;25:135. doi: 10.1186/s12876-024-03566-4 (PMC11881308; doi:10.1186/s12876-024-03566-4)

Summary statistics of Episcleritis:


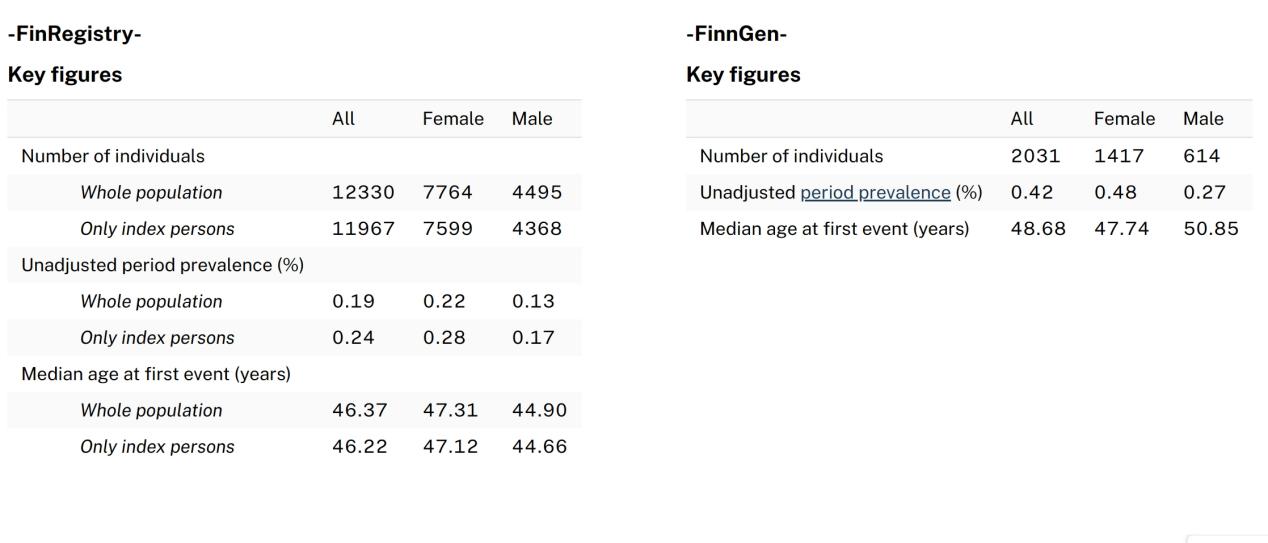


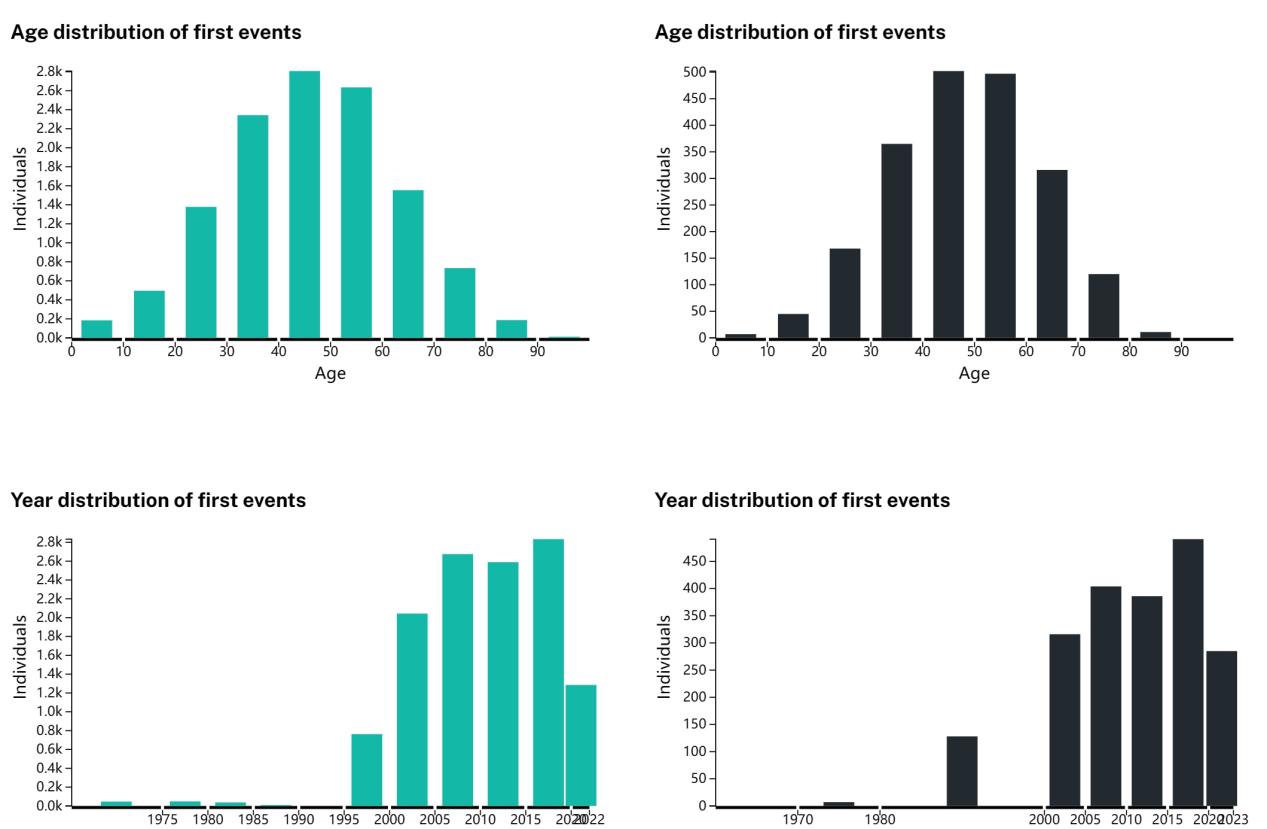


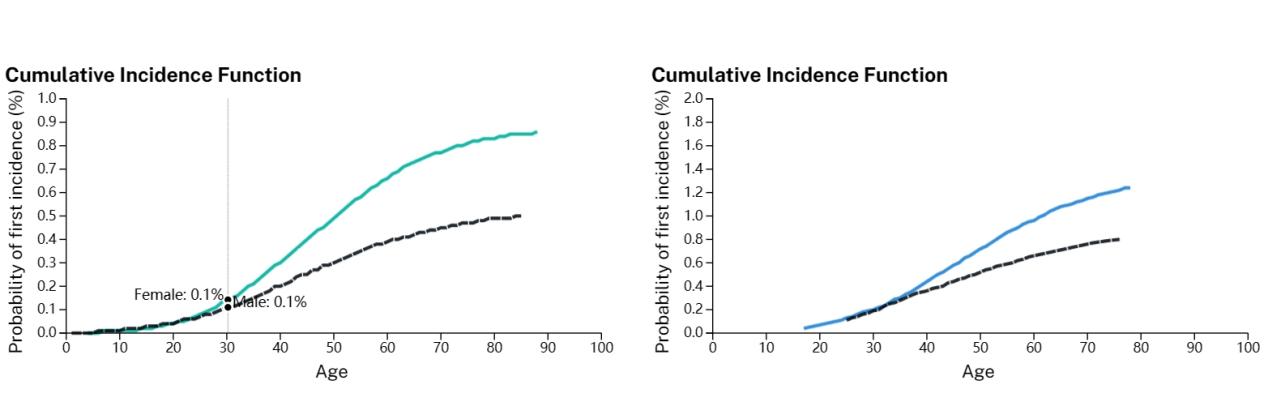


Summary statistics of Scleritis:


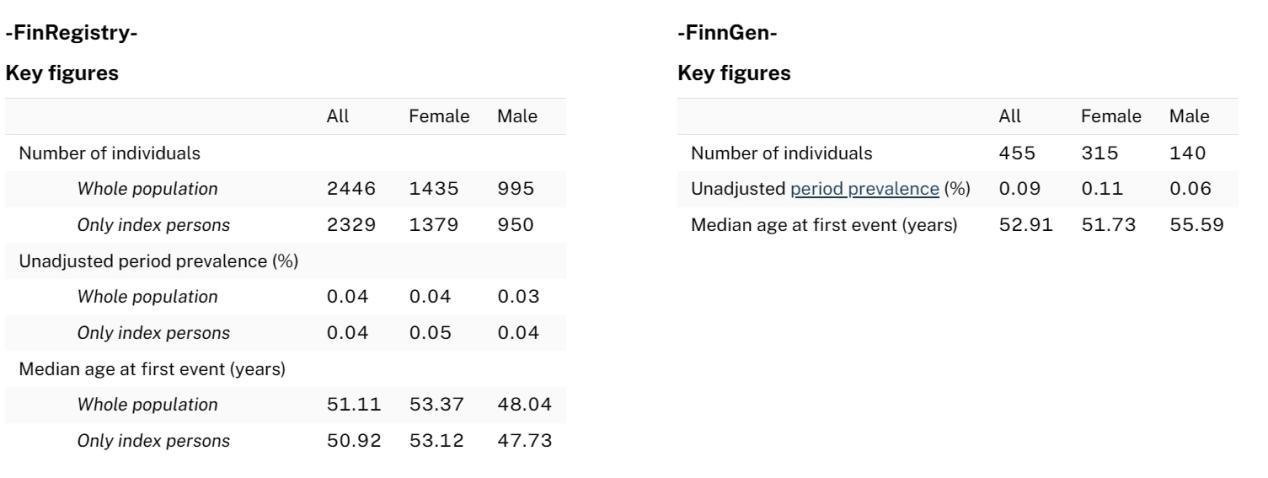


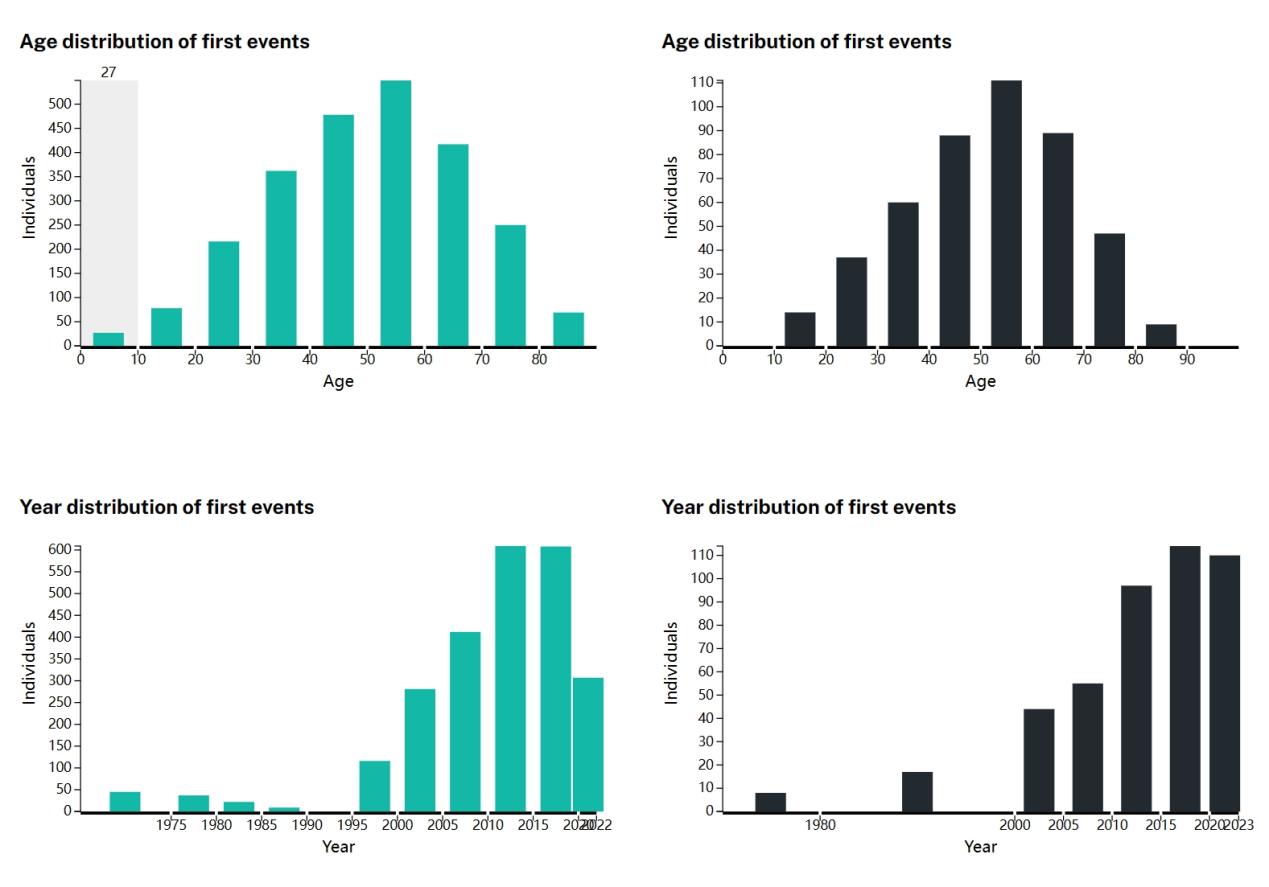


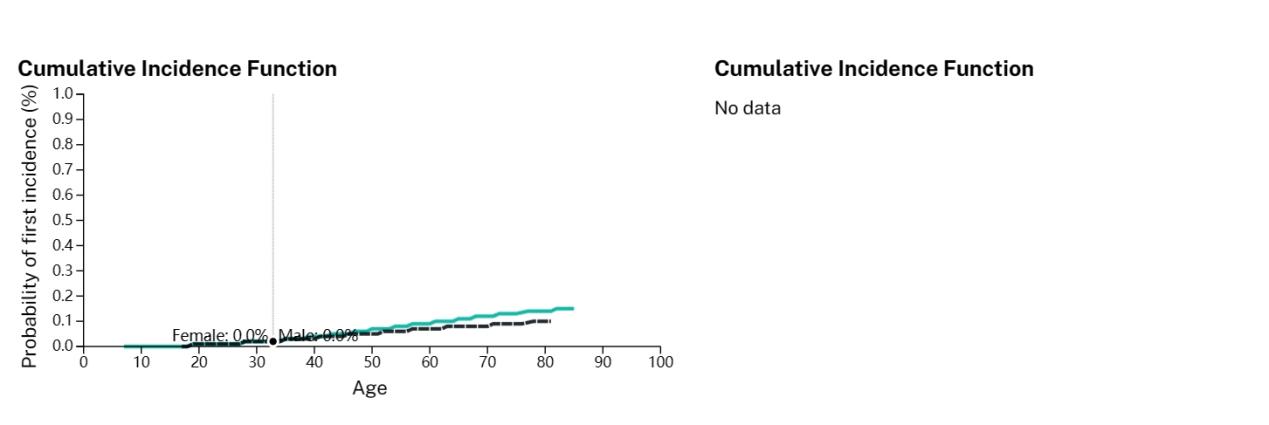


Summary statistics of Episcleritis:


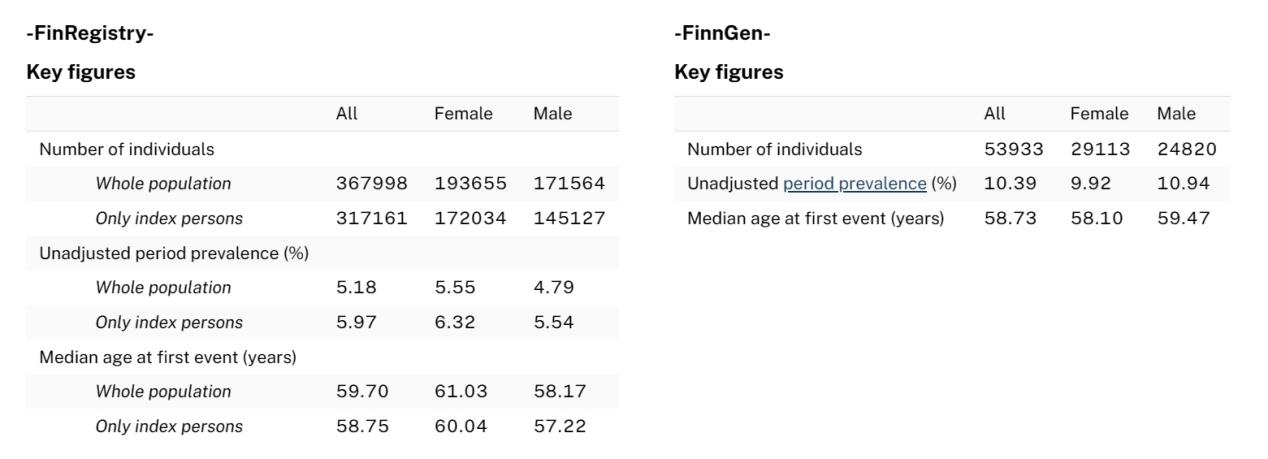


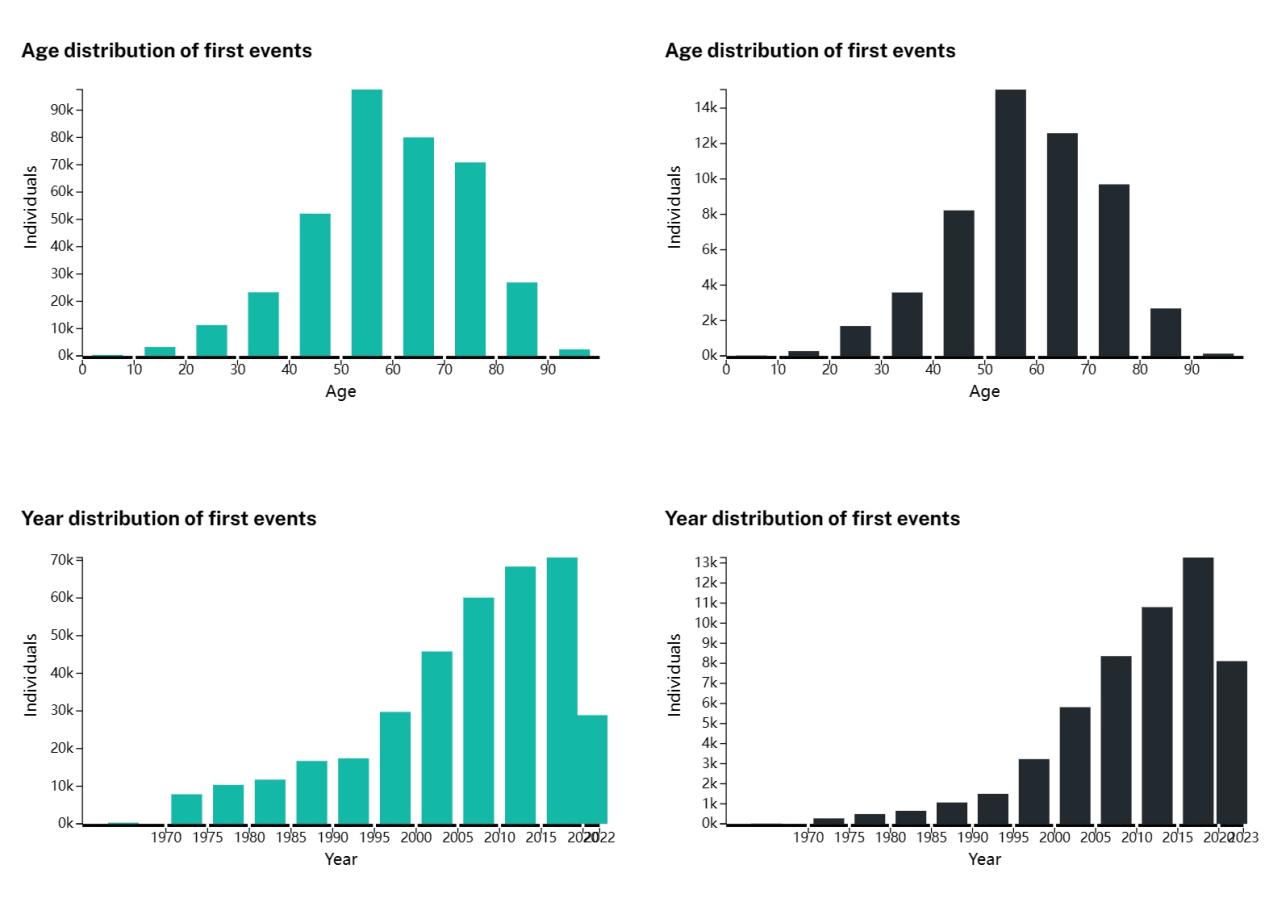


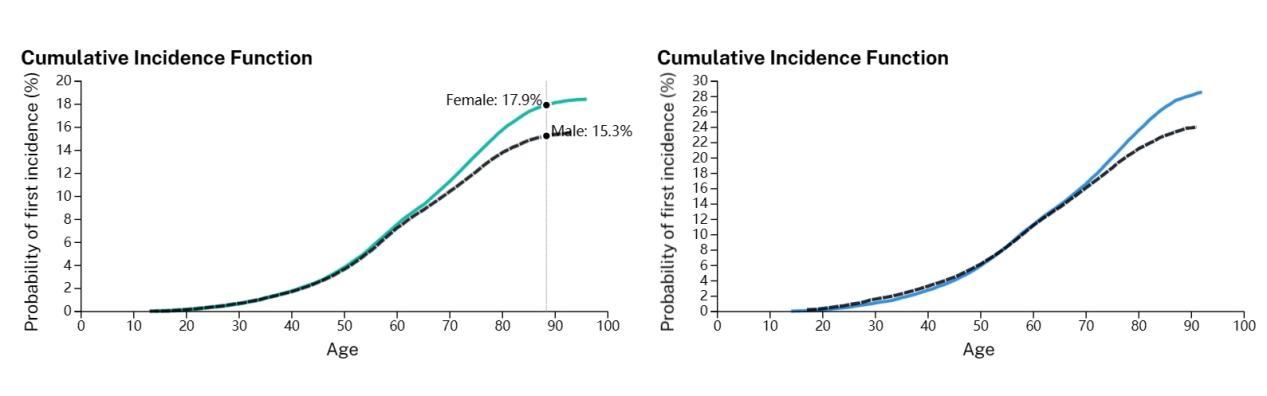


Summary statistics of Erythema nodosum:


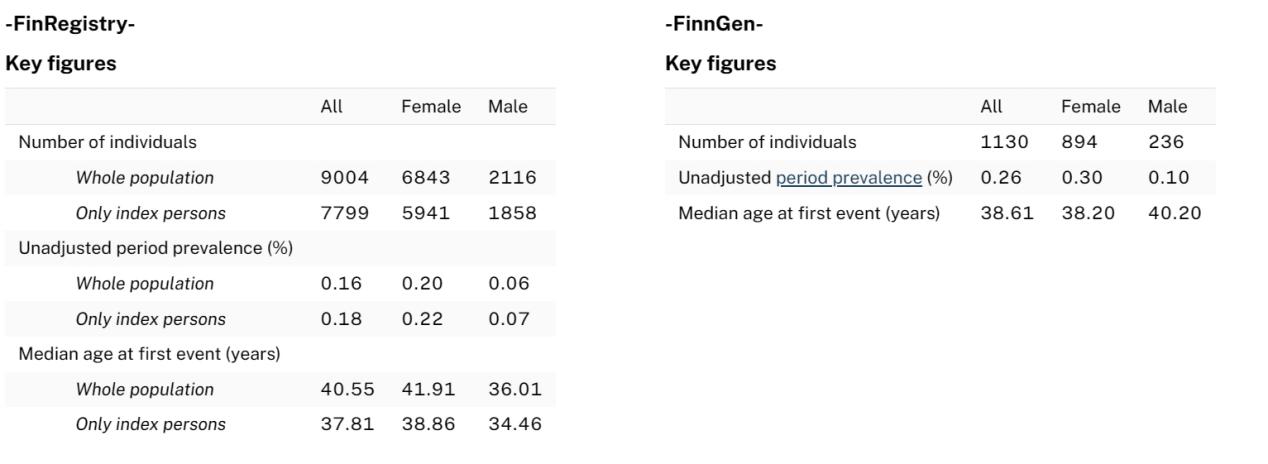


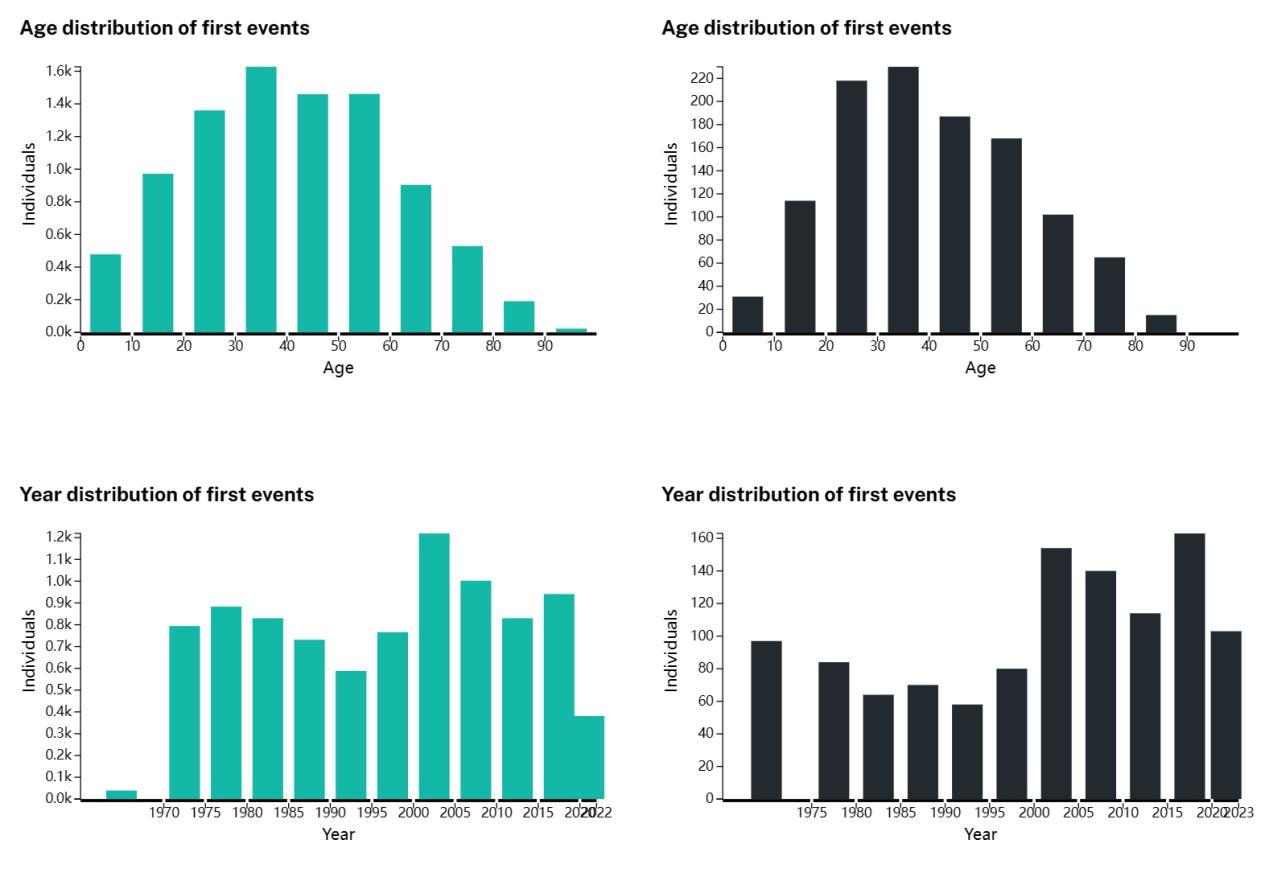


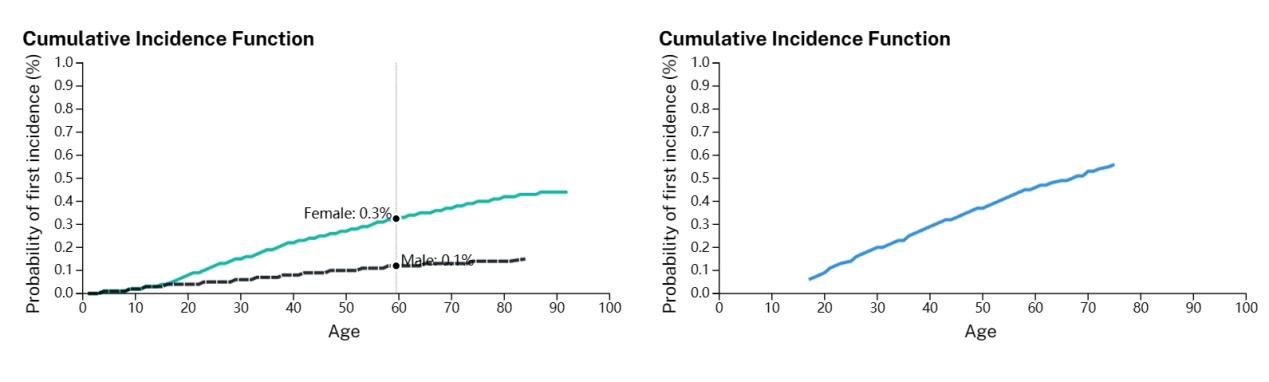

Supplement: Supplementary file 3 — Supplementary Material 3 [file 12876_2024_3566_MOESM3_ESM.docx]
